# Altered hepatic lipid metabolism in mice lacking both the melanocortin type 4 receptor and low density lipoprotein receptor

**DOI:** 10.1371/journal.pone.0172000

**Published:** 2017-02-16

**Authors:** Vera Lede, Andrej Meusel, Antje Garten, Yulia Popkova, Melanie Penke, Christin Franke, Albert Ricken, Angela Schulz, Wieland Kiess, Daniel Huster, Torsten Schöneberg, Jürgen Schiller

**Affiliations:** 1 Molecular Biochemistry, Rudolf-Schönheimer-Institute of Biochemistry, University of Leipzig, Leipzig, Germany; 2 Institute of Medical Physics and Biophysics, University of Leipzig, Leipzig, Germany; 3 Hospital for Children & Adolescents, Department of Women and Child Health, Center for Pediatric Research Leipzig, University of Leipzig, Leipzig, Germany; 4 Heart and Vascular Center, Bad Bevensen, Germany; 5 Institute of Anatomy, Medical Faculty, University of Leipzig, Leipzig, Germany; Northeastern Ohio Medical University, UNITED STATES

## Abstract

Obesity is often associated with dyslipidemia and hepatosteatosis. A number of animal models of non-alcoholic fatty liver disease (NAFLD) are established but they significantly differ in the molecular and biochemical changes depending on the genetic modification and diet used. Mice deficient for melanocortin type 4 receptor (*Mc4r*^*mut*^) develop hyperphagia, obesity, and subsequently NAFLD already under regular chow and resemble more closely the energy supply-driven obesity found in humans. This animal model was used to assess the molecular and biochemical consequences of hyperphagia-induced obesity on hepatic lipid metabolism. We analyzed transcriptome changes in *Mc4r*^*mut*^ mice by RNA sequencing and used high resolution ^1^H magic angle spinning NMR spectroscopy and MALDI-TOF mass spectrometry to assess changes in the lipid composition. On the transcriptomic level we found significant changes in components of the triacylglycerol metabolism, unsaturated fatty acids biosynthesis, peroxisome proliferator-activated receptor signaling pathways, and lipid transport and storage compared to the wild-type. These findings were supported by increases in triacylglycerol, monounsaturated fatty acid, and arachidonic acid levels. The transcriptome signatures significantly differ from those of other NAFLD mouse models supporting the concept of hepatic subphenotypes depending on the genetic background and diet. Comparative analyses of our data with previous studies allowed for the identification of common changes and genotype-specific components and pathways involved in obesity-associated NAFLD.

## Introduction

The non-alcoholic fatty liver disease (NAFLD) is the most common fatty liver disease in western countries and closely associated with obesity [1]. It is considered the hepatic manifestation of the metabolic syndrome and often accompanied by symptoms like hypertension, dyslipidemia and atherosclerosis. NAFLD covers a wide range of conditions varying from simple steatosis to inflammatory non-alcoholic steatohepatitis (NASH), which is a risk factor for cirrhosis with a significant liver-related mortality and a median survival of 6 years [2].

There is still limited knowledge about potential genetic predispositions and this, in combination with potential changes of the nutrition habits of the affected patients, makes it difficult to identify the factors which finally lead to the development of NAFLD [3]. Because of the manifold factors that may cause and influence subphenotypes in NAFLD, well-defined studies of human subjects are difficult to perform. Therefore, the development of suitable animal models is extremely important to obtain detailed insights into the pathogenesis of the disease. Animal models of NAFLD are divided into genetic, dietary, and combination models [4]. Classically, animals are fed a diet lacking choline and methionine [5] or enriched with up to 75% of fat (high fat diet, HFD) [6]. There are also diets enriched with compounds with known liver toxicity such as cholate [7, 8]. However, these diets have a serious impact on the liver metabolism which complicates the comparison with NAFLD and the related pathologic processes in humans.

Established genetic mouse models for NAFLD are ob/ob- and db/db mice which are both characterized by changes of the leptin signaling pathway. Ob/ob- and db/db mice lacking leptin and the leptin receptor, respectively, are hyperphagic and consequently develop all symptoms of the metabolic syndrome and steatosis already with regular chow diet [9, 10]. Although leptin levels correlate with obesity and steatosis, the influence of leptin in NAFLD is still under debate [11]. Furthermore, obesity-associated polymorphisms in the leptin or leptin-receptor do only rarely occur in humans [12]. Loss-of-function mutations in the human melanocortin type 4 receptor (*MC4R*) are associated with hyperphagia, severe early-onset obesity, increased longitudinal growth, fasting hyperinsulinemia, NAFLD and increased lean body mass. In humans, mutations of *MC4R* are the most frequent monogenic cause of severe early-onset obesity [13, 14]. Similar phenotypes found in mice [15, 16] and rats [17] harboring *Mc4r* defects, support an essential role for the melanocortin system in energy homeostasis across mammalian species [18]. Interestingly, the level of central nervous *Mc4r* activity potently determines the balance among hepatocellular glucose uptake, triacylglycerol (TAG) synthesis, lipid deposition, and lipid mobilization [19]. This is most probably because hyperphagia causes excessive energy supply already under regular chow. Therefore, the *Mc4r*-deficient mice more closely reflect energy supply-driven obesity and allow studying hepatic changes found in NAFLD but leaving the leptin system genetically intact.

Existing models of *Mc4r* null-mutant mice and rats [20, 21] as well as mice with a partial loss-function mutation [22] both develop the symptoms of the metabolic syndrome including significantly increased body weight, hyperlipidemia and hepatic steatosis. However, complete loss of the *Mc4r* occurs rarely in humans. In contrast, most mutations have been found to occur heterozygous and thus, lead to *MC4R* haploinsufficiency [23, 24] which is more closely resembled by the partial loss-of-function mutation [22]. Thus, we used mouse models with a partial loss-of-function mutation in the *Mc4r* which mimics mutations found in humans and consequently lead to the development of the *Mc4r*-associated symptoms of the metabolic syndrome including NAFLD. To study the impact of a proatherogenic lipoprotein profile or "lipid triad" frequently associated with obesity [25] we introduced a low density lipoprotein receptor knockout. This *Ldlr* deficiency partially blocks hepatic LDL reuptake subsequently causing dyslipidemia and a severe atherosclerosis already under regular chow [25]. In-depth analysis of the transcriptome of steatotic livers identified changes in metabolic pathways that most probably contribute to the pathogenesis of NAFLD. These findings were supported by comprehensive measurement of the lipid composition using mass spectrometric (MS) and nuclear magnetic resonance (NMR) spectroscopic methods. Altogether, we fine-characterized the hepatic lipid composition both, under native condition and in the diseased state of NAFLD, and and identified genotype-specific and common components related to pathologic storage of lipids in the liver.

## Materials and methods

### Mice and knockout models

The mouse strain used for this study carries an Ile^194^Phe mutation in the *Mc4r* (refer to as *Mc4r*^*mut*^), that leads to a partial loss-of-function *in vitro*, but causes the full obese phenotype *in vivo* and, therefore, closely mimics the receptor dysfunction most frequently found in humans [22]. *Mc4r*^*mut*^ mice were crossed onto a homozygous B6.*Ldlr*^*-/-*^ background (The Jackson Laboratory, Bar Harbor, Maine, stock no. 002207) to generate doubly mutant mice. Mice were bred and maintained under specific-pathogen-free conditions at the centralized animal care facility, where lights were automatically controlled (12 h light/12 h dark). Mice were fed with a regular chow or cholesterol-containing (0.02%) semisynthetic diet (composition given in S1 Table). Further details on genetic mouse characterization are given in the S1 Methods. All animal experiments were conducted in accordance with the European Directive 2010/63/EU on the protection of animals used for scientific purposes and were performed with permission of the Animal Care and Use Committee (ACUC #TVV 43/07) and the Government of the State of Saxony, Germany.

### RNA sequencing of liver transcripts

Total RNA from liver was extracted and indexed cDNA libraries were generated using TruSeq RNA Sample Preparation Kits v2 (Illumina, San Diego, CA, USA). Libraries with an average size of 300 bp were sequenced on Illumina HiScanSQ Sequencing System, performing ten biological replicates for each genotype. Processing and analyses of paired-end reads were performed using an established standard protocol (for details see S1 Methods).

### Measurements of NAD(H) and NADP(H) and Nampt enzyme activity

Nicotinamide adenine dinucleotide (NAD(H)) and nicotinamide adenine dinucleotide phosphate (NADP(H)) were measured using reversed-phase high performance liquid chromatography (HPLC) using the Chromaster Purospher STAR RP-18 endcapped 3 μm Hibar RT 150–3 HPLC column (Merck). 10 mg of frozen liver tissue was sonicated in 100 μl of 1 M perchloric acid. After a 10-min incubation period on ice samples were centrifuged and the supernatant was neutralized with 3 M potassium carbonate. After repeated centrifugation samples were loaded onto the column as described before [26]. Standard curves of NAD and NADP (10 μM to 200 μM) were used for quantification.

### ^1^H HR MAS NMR spectroscopy and MALDI-TOF mass spectrometry

For liver fat quantification, untreated liver tissue was subjected to ^1^H HR MAS NMR Spectroscopy using a Bruker Avance III 600 MHz NMR spectrometer (details see S1 Methods). A typical ^1^H HR MAS NMR spectrum of a liver sample and the assignments of most prominent signals is shown in S1 Fig. For MALDI-TOF mass spectrometry frozen liver tissue was extracted with chloroform/methanol (details see S1 Methods). MALDI-TOF mass spectra were recorded from lipid extract by using an Autoflex I mass spectrometer (Bruker Daltonics, Bremen, Germany). Mass spectra were analyzed using FlexAnalysis software version 2.2 (Bruker Daltonics).

### Statistical analysis

Statistical analyses for RNA sequencing and lipid composition were performed using open source R software (Version 2.15.2, The R Foundation). Statistical analyses of NAD metabolite assays were performed with GraphPad Prism^®^ software (5.03). The used tests are given in the table and figure legends.

## Results

### Effects of *Mc4r*- and *Ldlr* deficiency on hepatic lipid content

**Fig 1:**
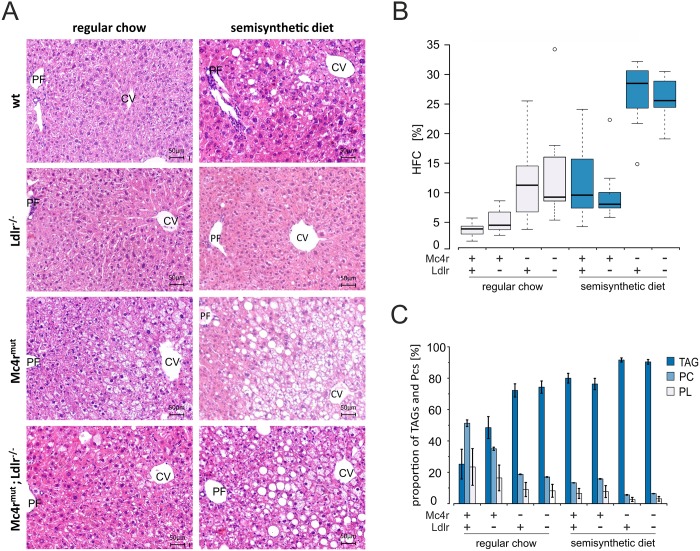
Hepatosteatosis and hepatic fat content assessed in wild-type (wt) and genetically modified mice under regular chow and semisynthetic diet. (**A**) Liver sections of wild-type (wt) and gene-deficient mice were HE stained (for details see S1 Methods). Fat vacuoles accumulated in the pericentral region when mice were lacking *Mc4r* alone or in combination with *Ldlr* or when fed a semisynthetic cholesterol-containing diet. CV central vein, PF portal field. Scale bars as indicated. (**B**) Hepatic fat content (HFC) was determined by quantitative ^1^H HR MAS NMR spectroscopy and calculated for the indicated mouse strains on the basis of the corresponding NMR spectra. Hepatic fat content data are reported as box-and-whisker plots. (**C**) Relative proportion of TAG, PC and PL of the total hepatic fat content. Means ± SD are given. For all statistical comparisons, a Kruskal-Wallis test was performed. Detailed information on p-values are given in S2 Table (n = 8–9).

As previously shown by us [22] and other groups [21, 27, 28], *Mc4r* deficiency leads to the metabolic syndrome accompanied by significantly increased body weight, hyperlipidemia and hepatic steatosis. Clear histological evidence for hepatic lipid accumulation was also present in livers of 6 months old *Mc4r*^*mut*^ and *Mc4r*^*mut*^;*Ldlr*^*-/-*^ mice fed with regular chow, whereas it was absent in their wild-type and *Ldlr*^*-/*-^ littermates (Fig 1A). Feeding a cholesterol-enriched semisynthetic diet led to hepatic steatosis in all genetic backgrounds, but was more prominent in *Mc4r*^*mut*^ and *Mc4r*^*mut*^;*Ldlr*^*-/-*^ mice (Fig 1A). To confirm these histological findings, direct quantitative information about the lipid content was obtained by ^1^H HR MAS NMR spectroscopy (Fig 1B). Under regular chow both, *Mc4r*^*mut*^ and *Mc4r*^*mut*^;*Ldlr*^*-/-*^ mice, had an approximately 3-fold higher proportion of lipids compared to the wild-type. Interestingly, semisynthetic diet *per se* led to a similar hepatic fat content in the wild-type (9%), whereas in combination with the *Mc4r*^*mut*^ or *Mc4r*^*mut*^;*Ldlr*^*-/-*^ hepatic lipid content again was increased by a factor of ~3 and reached 28% and 25% respectively. However, the proportion of hepatic lipids in mice deficient for *Ldlr* remained at the respective wild-type levels under both diets. Together with the very similar hepatic fat content in *Mc4r*^*mut*^ and *Mc4r*^*mut*^;*Ldlr*^*-/-*^, this all suggest that the increased hepatic lipid accumulation is independent of the *Ldlr* deficiency.

Although the liver fat content was increased in both, *Mc4r*^*mut*^ and *Mc4r*^*mut*^;*Ldlr*^*-/-*^ the lipid composition could be different depending on the genotype and diet. Therefore, we calculated the content of storage lipids from the ^1^H NMR spectra. As shown in Fig 1C, livers of wt mice under regular chow contain only 25% TAG and 51% PC. Significant higher TAG content accompanied by reduced PC and PL content were obvious in all mutant mice. The amount of TAG increased up to 48% in *Ldlr*^*-/-*^, whereas it reached over 70% in *Mc4r*^*mut*^ and *Mc4r*^*mut*^;*Ldlr*^*-/-*^ mice under regular chow. The only insignificant differences in the lipid composition between *Mc4r*^*mut*^ and *Mc4r*^*mut*^;*Ldlr* suggest, that most of the hepatic lipid accumulation in the obese mice are due to *Mc4r*^*mut*^. A very similar composition with over 75% TAGs and ~14% PCs was found in wt and *Ldlr*^*-/-*^ mice fed the semisynthetic diet, whereas *Mc4r*-deficient littermates showed even higher accumulation of storing lipids (> 90%). On the basis of our data, phosphatidylethanolamines were identified as the most abundant phospholipid class after PC. However, a more detailed evaluation of the phospholipid composition was beyond the scope of this study.

### Transcriptional changes in mice with hepatic steatosis

Genome-wide transcript levels were determined by RNA sequencing of livers of 6-month-old mice to further dissect the molecular phenotypes. Interestingly, livers of *Mc4r*^*mut*^, *Mc4r*^*mut*^;*Ldlr*^*-/-*^ and wild-type mice fed a semisynthetic cholesterol-containing diet showed a similar pattern in hepatic fat content and composition, despite clear differences in the peripheral phenotype of atherosclerosis and the trigger leading to steatosis (genetic vs. dietary). Thus, genome-wide expression analysis was used to compare all mouse strains and diet groups using wild-type animals under regular chow as reference data set.

**Fig 2:**
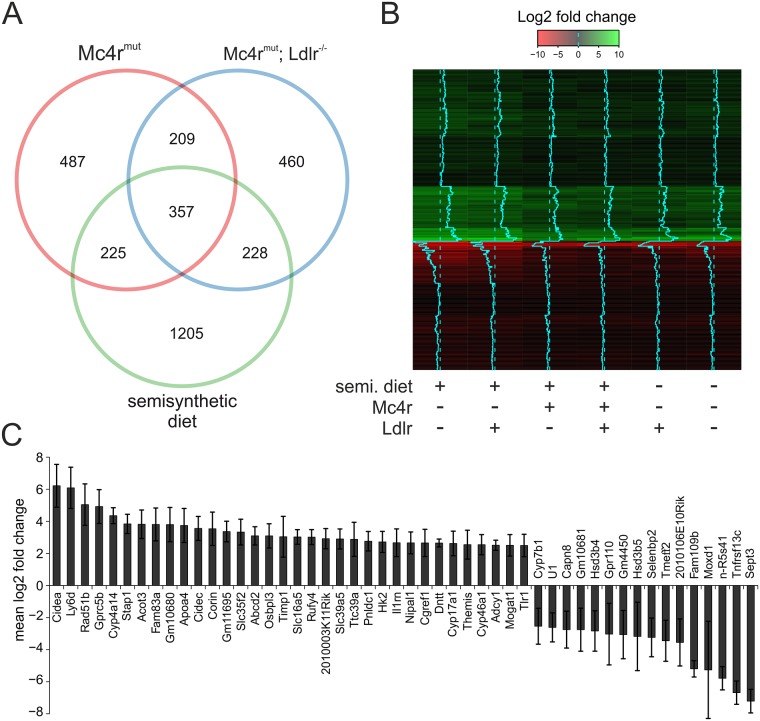
Common indicators of genetically and diet-induced NAFLD. (**A**) The numbers of differentially expressed genes in the liver of *Mc4r*^*mut*^ *(red circle)*, *Mc4r*^*mut*^;*Ldlr*^*-/-*^ (blue circle) mice under regular chow and of wt mice under semisynthetic diet (green circle) were compared to the wt data set under regular chow (served as reference) and plotted as Venn diagram. The total numbers of significantly up- and down-regulated genes (p < 0.05) are given in S3 Table. (**B**) The expression profiles of the 357 overlapping genes from the Venn diagram (Fig 2A) compared to the wt under regular chow (served as reference) for all mouse groups investigated are given as log2 fold change. The log2 fold change is color-coded with up- and down regulation of genes in green and red, respectively. Additionally, the blue trace in the middle of each lane gives the direction of quantitative changes by deviation from the dotted line (wt level under regular chow) to the right (up regulation) or to the left (down regulation). The genotypes and diets are given below the heat map (+ with, — without). (**C**) Average log2 fold change of the 50 strongest regulated genes that reached statistical significance across all differential expression analyses (p < 0.05).

We first ask the question whether there is subset of differentially expressed genes found in three hepatosteatotic mouse groups with the very similar lipid profile (*Mc4r*^*mut*^ regular chow, *Mc4r*^*mut*^;*Ldlr*^*-/-*^ both diets). Among the large number of differentially expressed genes between each group (complete list upon request) there were 357 genes that were significantly different expressed in this three groups compared to wt mouse fed with regular chow (Fig 2A). To validate these candidates as potential common changes related to NAFLD, the expression differences (direction and fold change) compared to the wt under regular chow were calculated. As visualized in Fig 2B, the expression profile of all 6 hepatosteatotic groups showed a very similar pattern of changes compared to the non-hepatosteatotic wild-type under regular chow. 256 of those genes revealed statistical significance across all hepatosteatotic groups. Since this set of differentially expressed genes overlapped regardless diets and genetic alterations but shares the phenotype of hepatosteatosis, we refer this as to common indicators of NAFLD. The 50 strongest regulated genes are shown in Fig 2C.

**Fig 3:**
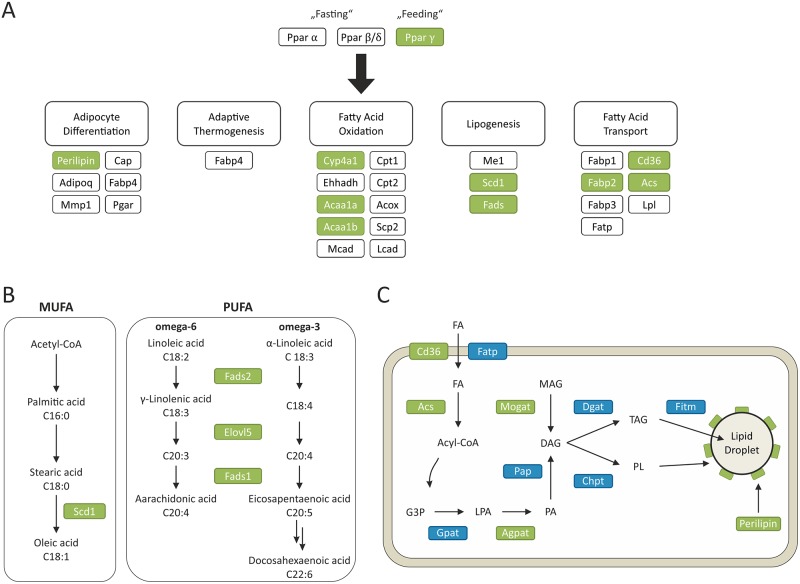
Differential regulation in hepatic lipid metabolism-related genes determined by RNA-sequencing. (**A**) Peroxisome proliferator-activated receptors (*Ppar*) regulate a number of lipid-related metabolic and cellular pathways. Genes which are differentially regulated in all hepatosteatotic mice (regular chow and semisynthetic diet fed *Mc4r*^*mut*^, *Mc4r*^*mut*^;*Ldlr*^*-/-*^, the semisynthetic diet fed wild-type (wt)) compared to wt under regular chow, are depicted in green and key components which are not differentially expressed are in blue. (**B**) Specifically, components involved in biosynthesis of unsaturated free fatty acids (FFAs) were regulated. (**C**) Differentially regulated genes which are involved in lipid transport and storage were significantly higher expressed in *Mc4r*-deficient mouse strains and the wt under semisynthetic diet. Detailed data are given in S5 Table.

To further categorize these molecular phenotypes enrichment analyses of the Kyoto Encyclopedia of Genes and Genomes (KEGG) pathways [29] was conducted using the Correlation Adjusted Mean Rank gene set test (CAMERA) from the R-package ‘limma’ [30]. Sixteen significantly (FDR < 0.05) enriched KEGG pathways were found in the liver transcriptome of *Mc4r*^*mut*^ and *Mc4r*^*mut*^;*Ldlr*^*-/-*^ under both diets and wild-type mice fed with the semisynthetic cholesterol-containing diet (S4 Table). Among those pathways present in all hepatosteatotic groups, several were closely linked to the lipid and energy metabolism as “Peroxisome proliferator-activated receptor (*Ppar*) signaling pathway”, “Biosynthesis of unsaturated fatty acids”, “Linoleic acid metabolism”, and “Citrate cycle”. *Ppars* are well known master regulators of lipid metabolism and influence a variety of processes such as synthesis, degradation and storage of lipids as well as adipogenesis and maintenance of metabolic homeostasis, all depending on the state of nutrition [31]. We found an upregulation of *Ppar γ* in both *Mc4r*^*mut*^ and *Mc4r*^*mut*^;*Ldlr*^*-/-*^ as well as in the semisynthetic-fed groups, whereas the other members of the receptor family *Ppar α* and *Ppar δ* remain unchanged (S5 Table). As illustrated in Fig 3, a number of genes involved in lipogenesis, fatty acid oxidation, transport, and storage were upregulated in the *Mc4r*^*mut*^ background and in the wild-type when fed a semisynthetic diet compared to livers from wt mice under regular chow. These transcriptional data indicate a massive *de novo* synthesis of unsaturated storage lipids in *Mc4r*^*mut*^. The lack of *Ldlr*, and, therefore, a reduced lipid reuptake from the periphery, appears to be less relevant for hepatosteatosis. However, some additive effects of both, *Mc4r*- and *Ldlr* deficiencies on expression levels (e.g.: *Acot9*, *Apoc2*, *Cd36*, *Egfr*, *Lipo1*, *Morc4*, *Plin4*, *Ppap2c*) but also “neutralizing” effects (e.g. *Chpt1*, *Enc1*, *Ermp1*, *Fabp2*, *Hmgcl*, *Gpr98*, *Mogat1*, *S100a11*) were observed (S5 Table).

Next we asked whether there is a specific subset of differentially expressed genes only found in mice fed on the semisynthetic diet. As summarized in S6 Table, 110 genes were differentially expressed between the two diets. However, analysis revealed only two KEGG pathways (“cytosolic DNA sensing pathway”, “steroid hormone biosynthesis”) regulated category related to the semisynthetic diet regardless the genetic alteration.

*Ldlr* deficiency was introduced to evaluate the impact of a proatherogenic lipoprotein profile frequently associated with obesity. Thus, the RNA sequencing data were analysed with respect to significant changes private to *Ldlr* deficiency. However, there were only 30 transcripts differentially expressed exclusively in all *Ldlr*^*-/-*^ background. As stated above, the impact on *Mc4r* caused hepatic transcriptional changes was marginal.

We finally ask the question of whether the mouse strains show any signatures of hepatic inflammation. None of the mouse lines investigated showed significant hepatic leukocyte infiltrations as checked by anti-CD45 immunohistochemistry (data not shown). Also, KEGG pathway analysis revealed no results related to immune response consistently significant across all hepatosteatotic groups (S4 Table) and specific analysis of mRNA levels of markers for leukocytes (*Cd45*), T cells (*Cd28*, *Csf2*, *Ccr5*, *Cxcr4*) and B cells (*Pax5*, *Cd79b*) supported the absence of significant immune cell infiltration and NASH at this stage of NAFLD development. Interestingly, *Cd4* (T cell marker) was significantly downregulated in all steatotic liver samples (S5 Table).

### NAD levels in hepatic steatosis

**Fig 4:**
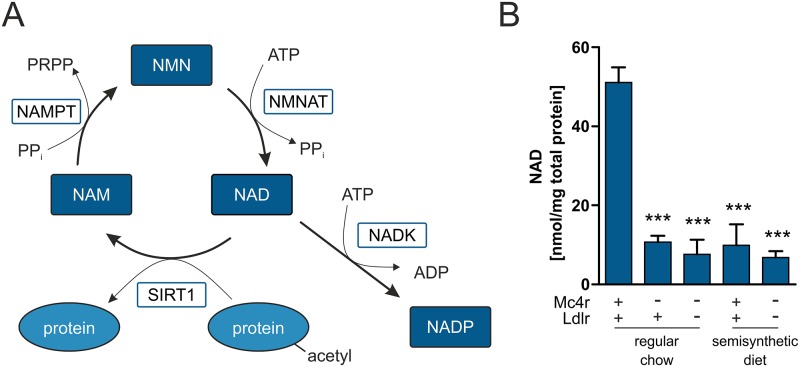
NAD is reduced in livers of genetically modified compared to wild-type mice. (**A**) Schematic overview of the *Sirt1*-NAD salvage pathway and involved components. (**B**) NAD was quantified using reversed-phase HPLC. Data represent means ± SEM. Significance levels were calculated by one way analysis of variance followed by Bonferroni post hoc test (n = 6).

Several studies already showed that the NAD metabolism plays a crucial role in all stages of the development of NAFLD by regulating energy homeostasis in the liver [32]. The expression of enzymes involved in NAD biosynthesis was found to be altered (S5 Table). Nicotinamide phosphoribosyltransferase (*Nampt*) and NAD kinase (*Nadk*) expression levels were increased up to 1.3 fold in both *Mc4r*^*mut*^ and *Mc4r*^*mut*^;*Ldlr*^*-/-*^ compared to the wt control. *Nampt* catalyzes NAD salvage from nicotinamide and *Nadk* phosphorylates NAD to NADP, an essential cofactor for fatty acid biosynthesis and cytochrome P450 enzymatic activity (Fig 4A). Therefore, NAD concentrations were measured in liver tissue. NAD was found to be decreased in all mouse strains with genetic and diet-induced hepatosteatosis compared to regular chow wt controls (Fig 4B).

Although the NAMPT protein levels remained unchanged (S3A Fig), NAMPT activity was 2.4-fold higher in *Mc4r*^*mut*^;*Ldlr*^*-/-*^ compared to wt controls (54.10 ± 15.33 cpm/μg total protein x h vs. 22.07 ± 1.79 cpm/μg total protein x h) (S3A Fig). NADK protein as well as NADP levels were unchanged in all mouse groups (S3B and S3C Fig). Interestingly, in *Mc4r*^*mut*^;*Ldlr*^*-/-*^ compared to the wt the amount of NAD dependent deacetylase SIRT1 was significantly higher (by 2.2-fold) while global lysine acetylation was decreased by 1.3-fold.

### Changes in hepatic lipid composition during NAFLD

**Fig 5:**
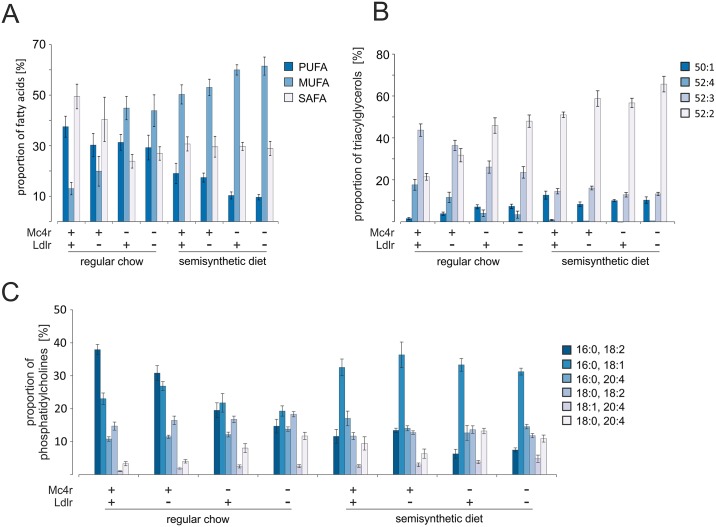
Contents of saturated and unsaturated fats in livers of wild-type and genetically modified mice. (**A**) The average percentage of PUFAs, MUFAs and SAFAs determined from the ^1^H HR MAS NMR spectra is shown for the indicated mouse strains. (**B**) The relative composition of TAGs was determined by positive ion MALDI-TOF MS. Minor TAGs, which are not included in this figure, are given in the S2 Table. (**C**) The relative PC composition was determined by MALDI-TOF mass spectrometry. Minor PC species not included in this chart are given in S2 Table. All data are given as mean ± SEM. For all statistical comparisons, a Kruskal-Wallis test was performed. Detailed information on p-values are given in S2 Table (n = 8–9).

The KEGG pathway analysis of hepatic transcriptomes from *Mc4r*^*mut*^, *Mc4r*^*mut*^;*Ldlr*^*-/-*^ and semisynthetic diet-fed wt mice suggested changes in the biosynthesis of unsaturated fatty acids. Therefore, the average double bond content per each of the most abundant lipids was calculated on the basis of the ^1^H HR MAS NMR spectra. For liver tissue of wt mice, the calculated average PUFA content was 37.5%, while the MUFA fraction was only about 13.1%. The inactivation of the *Mc4r* led to a slight reduction of PUFA content to 30%, but a 3-fold increase of MUFAs. Since the content of SAFAs was decreased by the factor of 2, MUFAs replaced SAFAs as most abundant lipid fraction (Fig 5A). *Ldlr* deficiency again had little to no effect since no significant differences were detected neither between wt and *Ldlr*^*-/-*^ nor between *Mc4r*^*mut*^ and *Mc4r*^*mut*^;*Ldlr*^*-/-*^ under both diets. Interestingly, when fed a semisynthetic cholesterol-containing diet already the wt revealed a similar increase of MUFAs and decrease of SAFAs as the *Mc4r*^*mut*^ littermates under regular chow, whereas the reduction of PUFAs was even stronger. The combination of *Mc4r* deficiency with the semisynthetic diet led to a further increase in MUFAs and decrease in PUFA, while the SAFA content remained at 30% (Fig 5A).

Differences in the lipid composition of the tissue samples as indicated by ^1^H HR MAS NMR spectroscopy were further analyzed by soft ionization MALDI-TOF mass spectrometry. A typical MALDI-TOF mass spectrum of a liver tissue sample is shown in S2 Fig. By mass spectrometry, highly abundant individual lipid species could be identified in the tissue extracts, which can be nearly exclusively assigned to TAGs [33]. The overall fatty acyl composition was determined by MALDI-TOF mass spectrometry and the relative concentration of characteristic TAGs in mouse liver tissue is shown in Fig 5B. Wild-type liver tissue was rich in polyunsaturated TAGs such as 52:3 and 52:4 (number of carbon atoms: number of double bonds) which together accounted for 60% of the total TAGs. In contrast, liver tissue of *Mc4r*-deficient mice contained predominantly TAG 52:2 and only 5% of 52:4. Although *Ldlr*^*-/-*^ showed a slightly altered lipid composition when compared to the wt under regular chow, additional inactivation of the *Ldlr* (*Mc4r*^*mut*^;*Ldlr*^*-/-*^) had no significant influence on the lipid and fatty acyl composition compared to *Mc4r*^*mut*^. Mice fed the semisynthetic diet had elevated levels of 52:2 and 50:1, whereas the proportion of highly unsaturated TAGs was considerably reduced compared to the wt under regular chow.

As already shown above for the TAG composition, the composition of the PCs was also influenced by the genetic background. Thus, tissue contents of the most abundant phospholipids POPC (16:0 / 18:1 PC), PLPC (16:0 / 18:2 PC), PAPC (16:0 / 20:4 PC), OAPC (18:1 / 20:4 PC), and SAPC (18:0 / 20:4 PC) were measured and are shown in Fig 5C. Tissue from wt mice under regular chow was characterized by a high amount of POPC and PLPC, accounting for about 60% of the total PC fraction. PC species with arachidonoyl residues were found only in small amounts with PAPC as the most abundant representative (10%) of total PCs. As for TAG, *Ldlr*^*-/-*^ had no significant influence on the PC composition, but the *Mc4r*^*mut*^ caused significant alterations. The PLPC content decreased strongly from about 38% to 19% and 14% for *Mc4r*^*mut*^ and *Mc4r*^*mut*^;*Ldlr*^*-/-*^, respectively, while the relative percentage of highly unsaturated PC species increased (Fig 5C). In contrast, the content of arachidonoyl residues doubled in the liver of *Mc4r*-deficient mice compared to the wt. Increased proportion of POPC was found independent of the genotype in all semisynthetic diet-fed groups, which accounted for > 30% of the total PCs. Although the strong decrease of the proportion of PLPC as well as the increased proportion of PC species with arachidonoyl residues was observed in all semisynthetic diet-fed groups, the changes were more pronounced in the *Mc4r*^*mut*^ mice.

### Upregulation of perilipins in steatotic livers

**Fig 6:**
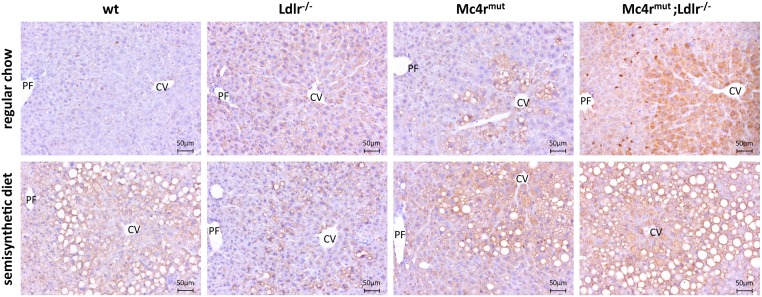
Increased perilipins in the hepatosteatotic liver tissue. Staining of *perilipin 2* as marker for lipid droplets in liver sections revealed increased expression around the central vein (CV). For experimental details see S1 Methods. PF portal field. Scale bars as indicated.

Energy storage at times of food excess is a carefully coordinated process, which depends on regulatory signals for storage (lipogenesis) of TAG in lipid droplets. Protein families such as fat-inducing transmembrane proteins 1 and 2 (FITM1/FIT1, FITM2/FIT2), the cell death-inducing DFF45-like effector (CIDE) protein family, and the perilipin family, are lipid droplet targeting protein families that promote association of lipid droplets with mitochondria [34]. RNA sequencing analysis revealed significant increase in transcripts of *perilipin 2*, *Cidea* and *Cidec* in all hepatosteatotic mice (S5 Table). Immunohistochemistry showed massive *perilipin 2* staining surrounding the central vein of *Mc4r*^*mut*^;*Ldlr*^*-/-*^ liver sections (Fig 6) and less intense also in *Mc4r*^*mut*^ mice under regular chow. Some immune-positive staining was also present in the *Ldlr*^*-/-*^, but showed a rather diffuse distribution across the hepatic lobule. For all genotypes, an increased accumulation of lipid droplets in combination with positive *perilipin 2* staining was present under the semisynthetic diet.

## Discussion

NAFLD represents a spectrum of diseases with increasing incidence in the industrialized countries but with limited treatment options. There is still a lack of knowledge of the pathogenic pathways leading from hepatic lipid accumulation to the progression to NASH, which then may lead to the development of liver cirrhosis [35]. Therefore, the elucidation of the molecular mechanisms of hepatic lipid accumulation merits increasing scientific interest. This is reflected by a number of animal models, in which the molecular development of hepatic steatosis and possible treatments are studied. It is beyond doubt that NAFLD is triggered by many factors including environmental and genetic factors. Most animal models that develop NAFLD require high caloric diets or carry defects in genes that directly (expressed in liver) or indirectly (non-hepatically expressed, but act on the liver) influence liver metabolism.

The *Mc4r* is almost exclusively expressed in the hypothalamus and, after inactivation, peripheral phenotypes are all linked to hyperphagia. Here, we studied *Mc4r*-deficient mice which developed NAFLD already under regular chow over a period of 6 months. As shown in Fig 1, this development of NAFLD in *Mc4r*-deficient mice does not require feeding a hypercaloric and/or HFD as it is mostly required for NAFLD in other animal models [36]. Interestingly, the cholesterol-containing semisynthetic diet led to NAFLD (Fig 1) already in the wt mice although the metabolizable energy was only moderately increased in this diet (16.2 MJ/kg vs. 12.8 MJ/kg). One can speculate that the significantly higher carbohydrate content (S1 Table) contributed to the same hepatosteatotic effects usually seen with hypercaloric and high fat diets. Although a more pronounced total hepatic lipid accumulation was seen in *Mc4r*^*mut*^ mouse strains under semisynthetic diet (Fig 1) mostly quantitative differences in the lipid composition were observed in all mouse strains under semisynthetic diet. This indicates that most probably the energy load but not a specific component was responsible for hepatosteatosis in these mouse groups (see Figs 1 and 5, S2 and S5 Tables).

Animal models with alterations in feeding behavior (e.g., hyperphagia in ob/ob mice), which results from defects in the leptin signaling pathways, are among the most frequently used genetic models to study NAFLD. However, direct hepatic effects of leptin are controversially discussed [12] and it remains open whether metabolic differences between hyperphagic mouse models depend on the genetic defect. For example, only the transcripts of *Ppar γ* and stearoyl-CoA desaturase-1 (*Scd1*) (see S5 Table) were significantly regulated in livers of both *Mc4r*^*mut*^ and ob/ob mice, whereas *Ppar α*, sterol regulatory element-binding protein-1c (*Srebp-1c*/ *Srebf1*), fatty acid synthase (*Fasn*), acetyl-CoA carboxylase (*Acac*), and mitochondrial transcription factor A (*Tfam*) were not significantly regulated in all hepatosteatotic mouse groups when compared to ob/ob mice under AIN-93G diet (very similar to our semisynthetic diet) [37]. This indicates distinct phenotypes between these hyperphagic mouse models of NAFLD.

The molecular changes in livers of *Mc4r*^*mut*^ mice are mainly characterized by significant changes in transcript levels of the KEGG pathways “biosynthesis of unsaturated fatty acids” and “PPAR signaling pathway” and components of lipid storage (see Fig 3). *Ppars* are ligand-activated transcription factors and function as regulators of energy homeostasis in liver and many other organs. They can activate (*Ppar γ*) and inactivate (*Ppar α*) cellular programs such as the biosynthesis of fatty acids. Consistent with an increase in *Ppar γ*, the relative hepatic content of TAG significantly increased compared to PC and PL in *Mc4r*^*mut*^ and *Mc4r*^*mut*^;*Ldlr*^*-/-*^ but also to some extent in *Ldlr*^*-/-*^ (see S2 Table).

Changes in NAD metabolism were shown to be implicated in all stages of the development of NAFLD by regulating specific NAD-dependent histone deacetylases called sirtuins (*Sirts*) and thus, central processes like energy homeostasis, inflammation, and apoptosis in the liver. In accordance with other studies in NAFLD mice [38, 39], lower hepatic NAD levels were measured in all mouse strains compared to wt mice (Fig 4B). The NAD biosynthetic enzyme *Nampt* is a crucial regulator of intracellular NAD levels. Unexpectedly, expression and activity of the NAD salvage enzyme *Nampt* was higher in *Mc4r*^*mut*^;*Ldlr*^*-/-*^ mice compared to controls (S5 Table, S3A Fig). Reasons for the detected decrease in NAD levels could be an enhanced degradation of NAD by poly(ADP-ribose) polymerase 1 (*Parp-1*) [40] or *Cd38* [41]. However, no evidence for an up-regulation of these NAD-consuming enzymes was found in the *Mc4r*^*mut*^ livers (not shown). Hepatic NADK protein and NADP levels were unchanged in all mouse strains indicating that there is no increased conversion from NAD to NADP. Interestingly, SIRT1 protein, a NAD-dependent deacetylase, was significantly higher in *Mc4r*^*mut*^;*Ldlr*^*-/-*^ mice compared to controls. Liver-specific knockout mice develop hepatic steatosis and inflammation under HFD [42], while global transgenic mice overexpressing *Sirt1* were protected against hepatic steatosis caused by HFD [43]. In another diet-induced mouse model of early hepatic steatosis *Sirt1* activity was increased [44]. This could indicate a compensatory mechanism to protect the liver against the negative impact of massive lipid storage on inflammation and apoptosis. One could assume that the NAD resulting from higher NAMPT activity in *Mc4r*^*mut*^;*Ldlr*^*-/-*^ mice is consumed by the increased NAD-dependent deacetylase SIRT1 as indicated by reduced global lysine acetylation.

In contrast to this finding, increased expression levels of *Sirt1* targets *Ucp2* and *Ppar γ* were detected in *Mc4r*^*mut*^ and *Mc4r*^*mut*^*/Ldlr*^*-/-*^ mice (S5 Table), suggesting a down regulation of SIRT1 activity. However, transcription of both, *Ucp2* and *Ppar γ*, is controlled by multiple factors besides *Sirt1* and both are known to be up regulated in NAFLD [45, 46]. *Ucp2*, a mitochondrial protein, is involved in the regulation of hepatic reactive oxygen species whereas *Ppar γ* plays a major role in the hepatic lipid homeostasis.

*Mc4r* deficiency under regular chow has a pronounced effect on the contents of PUFA, MUFA und SAFAs. Thus, livers from *Mc4r*^*mut*^ mice have a significant decrease of SAFA under a simultaneous increase of the MUFA in TAG, PC and PL (see Fig 5). The effect of an additional *Ldlr* deficiency in a *Mc4r*^*mut*^ background on the hepatic lipid composition is rather marginal. There was also no KEGG pathway which is different in the double KO mouse strain under both diets compared to single KOs and wt (see S4 Table) although almost 500 genes are differentially expressed in *Mc4r*^*mut*^;*Ldlr*^*-/-*^ when compared to the other mouse strains. Interestingly, the relative amount of arachidonoyl residues in PC was increased in the *Mc4r*^*mut*^ background (S2 Table). This is in line with the functional enrichment analysis indicating this KEGG pathway as regulated between the wild-type and *Mc4r*^*mut*^ or *Mc4r*^*mut*^; *Ldlr*^*-/-*^. One can speculate that the changes in arachidonic acid, prostaglandin and leukotriene biosynthesis (*Fads1*, *Fads2*, *Elovl5*; S5 Table) may contribute to the inflammatory response later in the pathogenesis of NASH.

In a recent paper several mouse strains with an alimentary NAFLD were compared at the transcriptomic levels and revealed a reasonable diversity among the mouse strains. However, a list of high-confidence candidate genes for hepatic steatosis, which significantly correlate with hepatic TAG levels, was extracted from the transcriptome data [47]. Comparing those TAG level-related transcripts with our data from *Mc4r*^*mut*^ and *Mc4r*^*mut*^;*Ldlr*^*-/-*^ liver transcripts only 50% und 56% (regular chow) and 54% and 54% (semisynthetic diet) of the transcripts, respectively, were found differentially expressed with statistical significance (see S7 Table). Again, this indicates different molecular phenotypes depending on the diet and genetic background of NAFLD. However, the fatty acid translocase *Cd36* seems to be a consistent marker being differentially regulated in many different models of NAFLD in animals and humans [47–49]. Perilipins are also constant markers of NAFLD as they were significantly upregulated in steatotic livers (see S5 Table, Fig 6) and also in other animal models of NAFLD and humans with hepatosteatosis [34, 50, 51]. Specifically, we found *perilipin 2* significantly increased in liver transcriptomes of hepatosteatotic mice when compared to livers from wt mice (p < 0.05).

An interesting finding is the significant downregulation of the *Avpr1a* mRNA expression in the livers of all hepatosteatotic groups (S5 Table). The *Avpr1a* is well known to be involved in the regulation of hepatic blood circulation [52], but there is also evidence for metabolic relevance of vasopressin function in liver including stimulation of hepatic glucose release, gluconeogenesis, ureagenesis, and fatty acid esterification [53]. Therefore, we consider reduction of *Avpr1a* mRNA expression as a constant indicator for NAFLD development. Besides the already named genes, there are many others that are shared among our NALFD-developing mouse strains and which may be common indicators of NAFLD.

Taken together, hyperphagia due to *Mc4r* deficiency causes NAFLD already under regular chow and induces a program designated to synthesize and store TAG in hepatocytes. The semisynthetic diet can result in metabolic differences between the different animal models for NAFLD, but components for fatty acid transport and storage seem to be always recruited. In-depth transcriptome analyses of animal models for NAFLD, either variable to well-defined diets or genetic modifications, may help to further sort out general components involved in hepatic lipid storage as well as specific components related to the diet or genetic background.

## Supporting information

S1 Fig High resolution ^1^H magic angle spinning nuclear magnetic resonance (^1^H HR MAS NMR) spectrum of liver tissue recorded at a MAS frequency of 9 kHz and a temperature of 30°C. Intensities of signals marked blue are independent of the fatty acid type. Glycogen signals resonate in the range of 3.4–4.2 ppm and cannot be individually resolved.
(PDF)

S2 Fig Representative matrix-assisted laser desorption ionization—time of flight (MALDI-TOF) mass spectra of triacylglycerols (TGAs, A) and phosphatidylcholines (PCs, B). TAGs detected in positive ion modus form exclusively sodium adducts, while PC show both proton and sodium adducts. Proton adducts were largely suppressed by sodium acetate addition. Intensities are given in arbitrary units.
(PDF)

S3 Fig Changes in components of the NAD metabolism in wild-type and genetically modified mice. (**A**) NAMPT protein expression and activity was determined in a Western blot and functional assay (see S1 Methods). In Western blots GAPDH expression served as control (n = 3). (**B**) NAD kinase (NADK) protein levels were quantified in Western blots and referred to GAPDH expression. (**C**) NADP levels (n = 5) were quantified using reversed-phase HPLC (see Materials and methods). (**D,E**) SIRT1 protein and global lysine acetylation was measured by Western blot analyses. Data represent means ± SEM. For Western blots one representative blot is shown. Significance levels were calculated by one way analysis of variance followed by Bonferroni post hoc test.
(PDF)

S1 Table Composition of the diet used. Both diets were purchased from Ssniff GmbH (Soest, Germany). Composition is listed in the table. ^#^Metabolizable Energy calculated according to the pig formula, Annex 4 of the German feed regulation
(PDF)

S2 Table Lipid parameters measured by ^1^H HR MAS NMR spectroscopy and MALDI-TOF mass spectrometry of the liver tissue of mice with the indicated genotype and diet. PL comprises all phospholipids (with the exception of phosphatidylcholines (PC)) with phosphatidylethanolamine as the second most common phospholipid. Data are given as mean ± standard error of mean except for the total hepatic fat content, for which the median is given.
(PDF)

S3 Table Summary of differentially expressed genes in liver derived from RNA sequence data. RNA-sequencing was performed from liver mRNA of the different mouse lines. Approximately 14 million reads per animal (10 mice per group) were analyzed. The total number of differentially expressed genes (as determined with DEseq) depending on the genotype and diet fed compared to the wild-type (wt) under regular chow is given. The overlap of differentially expressed genes between the groups is also shown in Fig 2.
(PDF)

S4 Table Functional enrichment analysis of differentially expressed compared to wild-type mice under regular chow. All KEGG pathways which showed a FDR < 0.05 (Correlation Adjusted Mean Rank Gene Set Test (CAMERA) from the R-package ‘limma’) were considered statistically significant.
(PDF)

S5 Table Expression changes of transcripts referred in text and figures. Differentially expression analysis results for all transcripts that were mentioned in the manuscript. The log2 fold expression change and the p-value for the comparison with the wt under regular chow is given.
(PDF)

S6 Table Genes specifically regulated in either the *Mc4r*^*mut*^ or the *Ldlr*^*-/-*^ background or under semisynthetic diet. Gene lists were generated comprising only genes that were significantly regulated in all groups fed with semisynthetic diet, but in none of the regular chow fed mice. Similar lists for *Mc4r*^*mut*^ and *Ldlr*^*-/-*^ background were created respectively. Genes with a p-value < 0.05 were considered statistically significant. Log2 fold changes and p-values are given.
(PDF)

S7 Table Genes expressed in liver known to correlate with hepatic triacylglycerol (TAG) levels. 50 genes whose expression correlates with hepatic TAG levels in different diet-induced non-alcoholic fatty liver disease (NAFLD) mouse models [47] are compared with the results of the differential expression analysis in our mouse strains. + p value < 0.05, — p > 0.05.
(PDF)

S1 Methods Supplementary Methods. (PDF)
