# Supplementary material for: Altered hepatic lipid metabolism in mice lacking both the melanocortin type 4 receptor and low density lipoprotein receptor
Source: PLoS One. 2017 Feb 16;12(2):e0172000. doi: 10.1371/journal.pone.0172000 (PMC5313158; doi:10.1371/journal.pone.0172000)
Supplement: S1 Fig — Intensities of signals marked blue are independent of the fatty acid type. Glycogen signals resonate in the range of 3.4–4.2 ppm and cannot be individually resolved. (PDF) [file pone.0172000.s001.pdf]

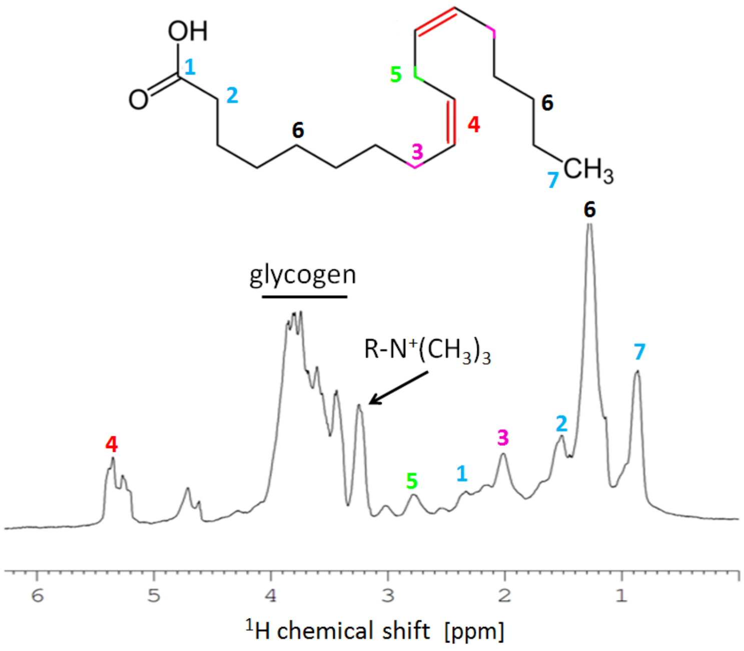

S1 Fig. High resolution ^{1}H magic angle spinning nuclear magnetic resonance (^{1}H HR MAS NMR) spectrum of liver tissue recorded at a MAS frequency of 9 kHz and a temperature of 30°C.

Intensities of signals marked blue are independent of the fatty acid type. Glycogen signals resonate in the range of 3.4 – 4.2 ppm and cannot be individually resolved**.**
