# Supplementary material for: Altered hepatic lipid metabolism in mice lacking both the melanocortin type 4 receptor and low density lipoprotein receptor
Source: PLoS One. 2017 Feb 16;12(2):e0172000. doi: 10.1371/journal.pone.0172000 (PMC5313158; doi:10.1371/journal.pone.0172000)
Supplement: S2 Fig — TAGs detected in positive ion modus form exclusively sodium adducts, while PC show both proton and sodium adducts. Proton adducts were largely suppressed by sodium acetate addition. Intensities are given in arbitrary units. (PDF) [file pone.0172000.s002.pdf]

A

B

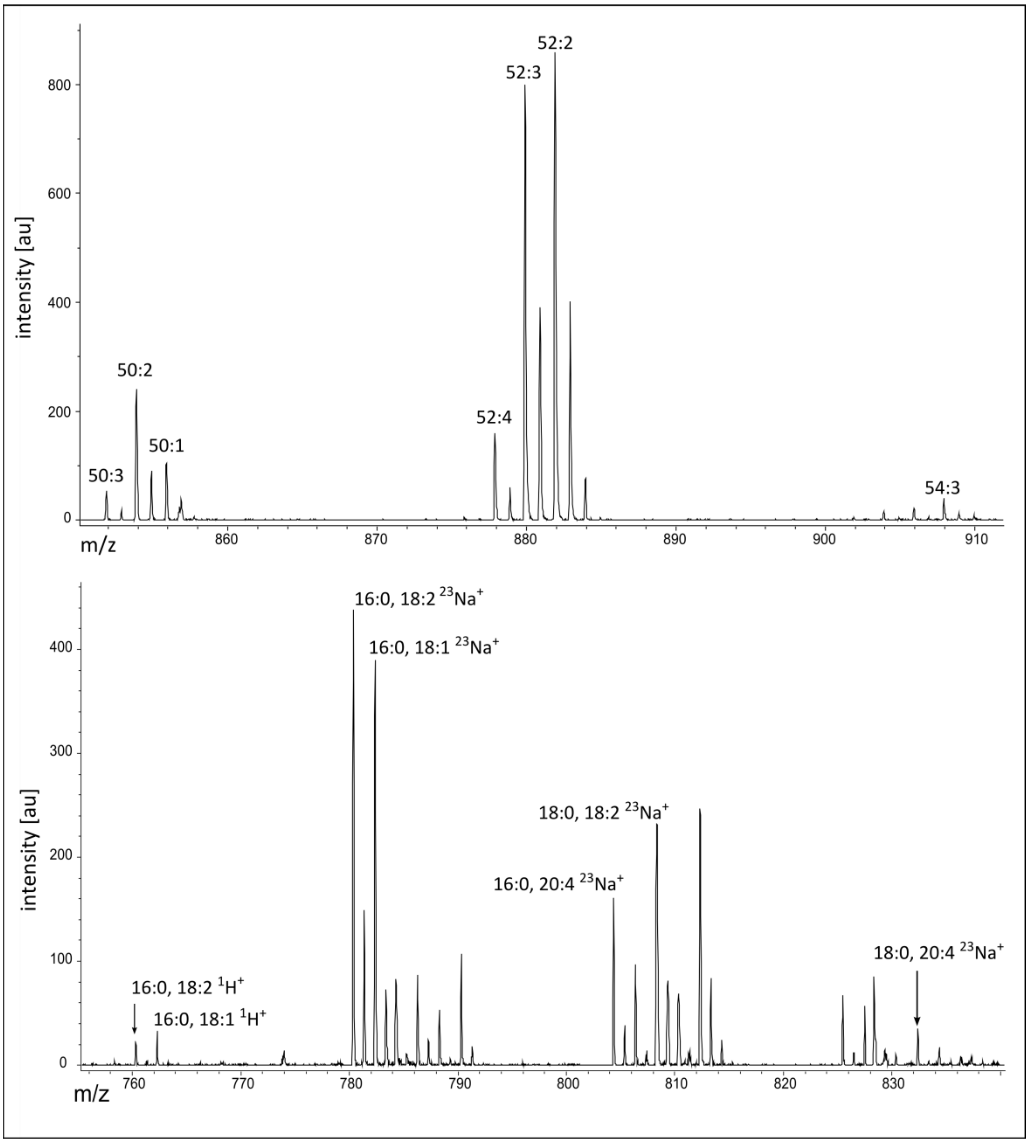

S2 Fig. Representative matrix-assisted laser desorption ionization — time of flight (MALDI-TOF) mass spectra of triacylglycerols (TGAs, A) and phosphatidylcholines (PCs, B).

TAGs detected in positive ion modus form exclusively sodium adducts, while PC show both proton and sodium adducts. Proton adducts were largely suppressed by sodium acetate addition. Intensities are given in arbitrary units.
