# Supplementary material for: Altered hepatic lipid metabolism in mice lacking both the melanocortin type 4 receptor and low density lipoprotein receptor
Source: PLoS One. 2017 Feb 16;12(2):e0172000. doi: 10.1371/journal.pone.0172000 (PMC5313158; doi:10.1371/journal.pone.0172000)
Supplement: S3 Fig — (A) NAMPT protein expression and activity was determined in a Western blot and functional assay (see S1 Methods). In Western blots GAPDH expression served as control (n = 3). (B) NAD kinase (NADK) protein levels were quantified in Western blots and referred to GAPDH expression. (C) NADP levels (n = 5) were quantified using reversed-phase HPLC (see Materials and methods). (D,E) SIRT1 protein and global lysine acetylation was measured by Western blot analyses. Data represent means ± SEM. For Western blots one representative blot is shown. Significance levels were calculated by one way analysis of variance followed by Bonferroni post hoc test. (PDF) [file pone.0172000.s003.pdf]

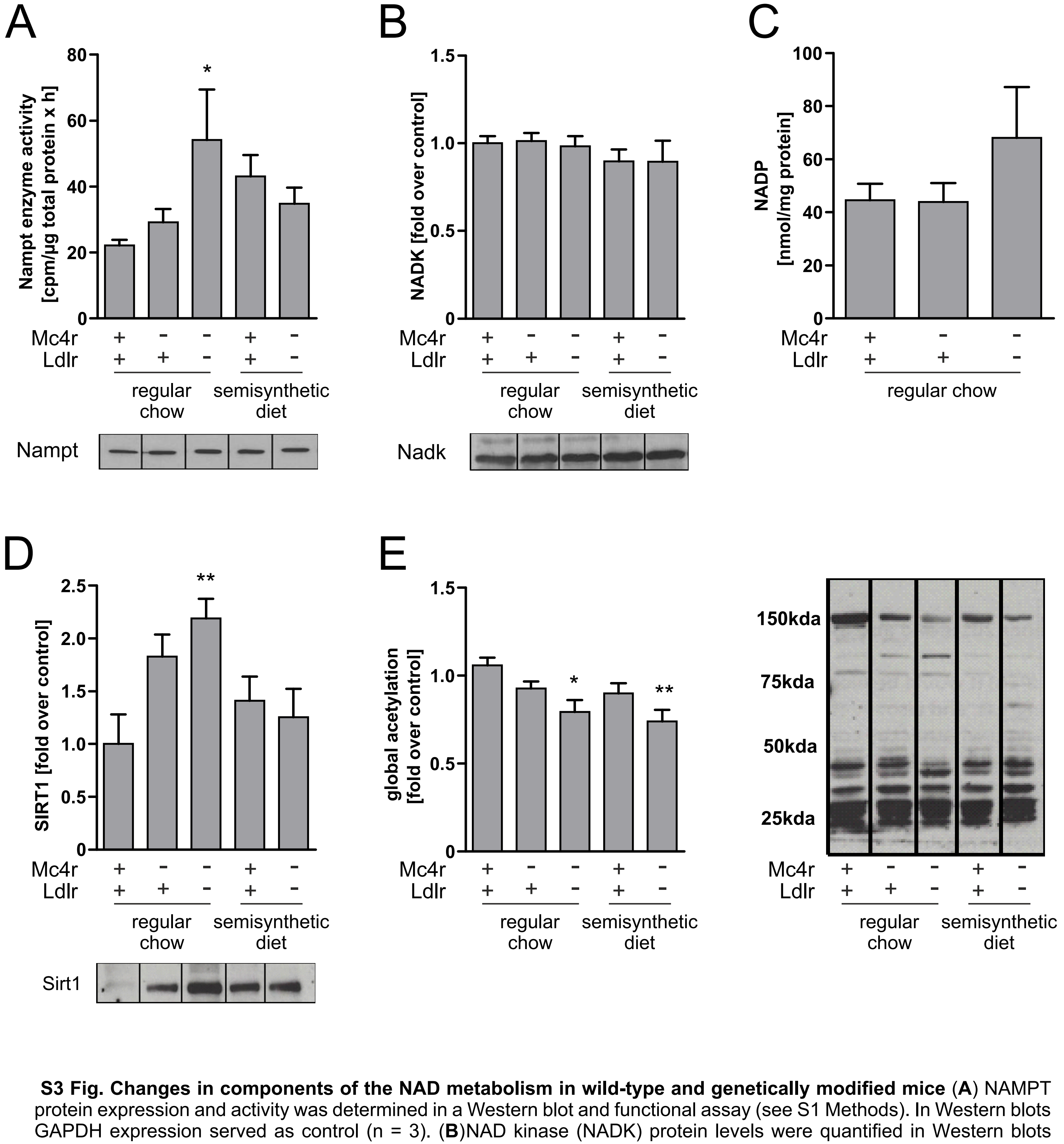

S3 Fig. Changes in components of the NAD metabolism in wild-type and genetically modified mice (**A**) NAMPT protein expression and activity was determined in a Western blot and functional assay (see S1 Methods). In Western blots GAPDH expression served as control (n = 3). (**B**)NAD kinase (NADK) protein levels were quantified in Western blots and referred to GAPDH expression. (**C**) NADP levels (n = 5) were quantified using reversed-phase HPLC (see *Materials and Methods*) (**D,E**) SIRT1 protein and global lysine acetylation was measured by Western blot analyses. Data represent means ± SEM. For Western blots one representative blot is shown. Significance levels were calculated by one way analysis of variance followed by Bonferroni post hoc test.
