# Supplementary material for: Altered hepatic lipid metabolism in mice lacking both the melanocortin type 4 receptor and low density lipoprotein receptor
Source: PLoS One. 2017 Feb 16;12(2):e0172000. doi: 10.1371/journal.pone.0172000 (PMC5313158; doi:10.1371/journal.pone.0172000)
Supplement: S1 Table — Both diets were purchased from Ssniff GmbH (Soest, Germany). Composition is listed in the table. #Metabolizable Energy calculated according to the pig formula, Annex 4 of the German feed regulation (PDF) [file pone.0172000.s004.pdf]

S1 Table. Composition of the diets used.

Regular chow and cholesterol-containing diet was purchased from Ssniff GmbH (Soest, Germany). Ingredients for each diet are listed in the table. #Metabolizable Energy calculated according to the pig formula (Annex 4 of the German feed regulation).

Component	Regular chow diet R/M H	Semisynthetic diet (AIN76A)
**Grossenergy**	**16.3 MJ/kg**	**19.0 MJ/kg**
**Metabolizable Energy**	**12.8 MJ/kg#**	**16.2 MJ/kg#**
**Crude nutrients in %**		
Dry matter	87.7	96.8
Crude protein	19.0	17.4
Crude fat	3.3	5.1
Crude fibre	4.9	5.0
Crude ash	6.4	2.0
N freeextracts	54.1	67.4
Starch	13.9	13.9
Sugar	4.7	51.0
**Cholesterol**	**0%**	**0.02%**
**Minerals in %**		
Calcium	1.0	0.63
Phosphorus	0.7	0.55
Sodium	0.24	0.11
Magnesium	0.22	0.05
Potassium	0.91	0.36
Ca:P		1.14
**Fattyacids in %**		
C 14:0	0.01	0.01
C 16:0	0.47	0.57
C 16:1	0.01	0.01
C 18:0	0.08	0.10
C 18:1	0.62	1.31
C 18:2	1.80	2.77
C 18:3	0.23	0.05
C 20:0	0.01	0.02
C 20:1	0.02	-
**Aminoacids in %**		
Lysine	1.00	1.43
Methionine	0.30	0.82
Cystine	not specified	0.08
Met+Cys	0.65	0.90
Threonine	0.68	0.77
Tryptophan	0.25	0.22
Agrinine	1.14	0.63
Histidine	0.44	0.55
Valine	0.88	1.18
Isoleucine	0.76	0.90
Leucine	1.30	1.70
Phenylalanine	0.85	0.93
Phe+Tyr	1.43	1.85
Glycine	0.80	0.36
Glutamicacid	3.90	3.90
Asparticacid	1.61	1.29
Proline	1.25	2.00
Alanine	0.79	0.57
Serine	0.89	1.03
**Vitamins per kg**		
Vitamin A	15,000 IU	4,000 IU
Vitamin D3	1,000 IU	1,000 IU
Vitamin E	110 mg	57 mg

Vitamin K (asmenadione)	5 mg	5 mg
Thiamin (B1)	18 mg	3 mg
Riboflavin (B2)	23 mg	5 mg
Pyridoxine (B6)	21 mg	6 mg
Cobalamin (B12)	100 µg	10 µg
Nicotinicacid	135 mg	29 mg
Pantothenicacid	43 mg	15 mg
Folicacid	7 mg	2 mg
Biotin	525 µg	20 µg
Choline-Chloride	2.990 mg	1.040 mg
Inositol	100 mg	not specified
**Trace elements per kg**		
Iron	179 mg	46 mg
Manganese	69 mg	59 mg
Zinc	94 mg	35 mg
Copper	16 mg	7 mg
Iodine	2.2 mg	0.22 mg
Selenium	0.3 mg	0.14 mg
Cobalt	2.1 mg	0.02 mg
