# Supplementary material for: Altered hepatic lipid metabolism in mice lacking both the melanocortin type 4 receptor and low density lipoprotein receptor
Source: PLoS One. 2017 Feb 16;12(2):e0172000. doi: 10.1371/journal.pone.0172000 (PMC5313158; doi:10.1371/journal.pone.0172000)
Supplement: S2 Table — PL comprises all phospholipids (with the exception of phosphatidylcholines (PC)) with phosphatidylethanolamine as the second most common phospholipid. Data are given as mean ± standard error of mean except for the total hepatic fat content, for which the median is given. (PDF) [file pone.0172000.s005.pdf]

S2 Table. Lipid parameters measured by 1H HR MAS NMR spectroscopy and MALDI-TOF mass spectrometry of the liver tissue of mice with the indicated genotype and diet.

	regular chow							semisynthetic diet							
	wt	$Ldlr^{-/-}$		$Mc4r^{mut}$		$Mc4r^{mut}/Ldlr^{-/-}$		wt		$Ldlr^{-/-}$		$Mc4r^{mut}$		$Mc4r^{mut}/Ldlr^{-/-}$	
	mean ± SEM	mean ± SEM	sign.	mean ± SEM	sign.	mean ± SEM	sign.	mean ± SEM	sign.	mean ± SEM	sign.	mean ± SEM	sign.	mean ± SEM	sign.
**Total hepatic fat content**															
**HFC**	3.3%	3.9%	/	10.8%	*	8.6%	*	8.9%	**	7.5%	/	28.2%	**	25.3%	**
**Total hepatic lipid composition**															
**TAG**	25.2 ± 9.5	48.5 ± 7.2	/	72.2 ± 4.2	***	74.3 ± 3.9	***	80.0 ± 3.1	**	76.3 ± 3.6	/	91.6 ± 1.3	*	90.4 ± 1.5	*
**PC**	51.3 ± 2.1	35.0 ± 1.0	/	18.7 ± 0.2	***	17.0 ± 0.2	***	13.3 ± 01.	**	15.8 ± 0.2	/	5.60 ± 0.2	*	6.40 ± 0.1	*
**PL**	23.4 ± 11.7	16.4 ± 8.2	/	9.0 ± 4.5	/	8.2 ± 4.1	/	6.5 ± 3.2	**	7.7 ± 3.8	/	2.7 ± 1.3	*	3.1 ± 1.5	*
**Saturation of hepatic fatty acids**															
**PUFA**	37.5 ± 4.1	31.0 ± 4.4	/	31.3 ± 3.1	/	29.3 ± 4.8	/	19.2 ± 3.9	**	17.4 ± 1.8	/	10.3 ± 1.3	/	9.6 ± 1.0	/
**MUFA**	13.1 ± 2.4	27.6 ± 5.3	/	44.8 ± 4.6	***	43.9 ± 6.2	***	50.3 ± 3.7	**	53.0 ± 3.2	/	59.9 ± 2.0	/	61.6 ± 3.5	/
**SAFA**	49.4 ± 4.8	40.8 ± 6.6	/	23.8 ± 2.6	/	26.9 ± 2.7	/	30.6 ± 2.8	**	29.6 ± 4.0	/	29.7 ± 1.5	/	28.9 ± 2.7	/
**Fatty acyl composition of liver phosphatidylcholines**															
**16:0,18:2**	37.9 ± 1.5	30.8 ± 2.2	/	19.5 ± 2.2	**	14.6 ± 2.0	***	11.5 ± 2.0	**	13.3 ± 0.6	/	6.2 ± 1.3	*	7.4 ± 0.6	/
**16:0,18:1**	23.0 ± 1.7	26.8 ± 1.3	/	21.2 ± 2.8	/	19.2 ± 1.5	/	32.5 ± 2.5	**	36.3 ± 3.8	/	33.2 ± 1.9	/	31.2 ± 1.0	/
**16:0,20:4**	10.7 ± 0.6	11.4 ± 0.4	/	12.1 ± 0.7	/	13.7 ± 0.6	*	17.0 ± 2.2	**	14.0 ± 0.7	/	12.6 ± 2.2	/	14.5 ± 0.6	/
**18:0,18:2**	14.6 ± 1.2	16.4 ± 1.2	/	16.7 ± .09	/	18.2 ± 0.7	*	11.6 ± 1.0	**	12.7 ± 0.5	/	13.6 ± 1.1	/	11.7 ± 0.6	/
**18:0,18:1**	4.4 ± 0.4	6.8 ± 1.4	/	7.2 ± 1.0	/	8.1 ± 0.5	*	5.4 ± 0.7	**	6.0 ± 0.8	/	7.1± 1.4	/	8.04 ± 0.4	/
**16:0,22:6**	5.9 ± 0.7	6.7 ± 0.3	/	8.8 ± 1.2	*	7.4 ± 0.3	*	7.2 ± 0.4	**	6.3 ± 0.4	/	6.1 ± 0.6	/	7.3 ± 0.9	/
**18:1,20:4**	1.0 ± 0.1	1.8 ± 0.3	/	2.4 ± 0.4	*	2.5 ± 0.4	*	2.5 ± 0.3	**	2.9 ± 0.5	/	3.7 ± 0.4	/	4.7 ± 1.1	/
**18:0,20:4**	3.2 ± 0.6	4.0 ± 0.5	/	8.0 ± 1.3	*	11.7 ± 1.1	**	9.4 ± 2.0	**	6.3 ± 1.4	/	13.2 ± 0.8	/	10.9 ± 1.0	/
**18:0,22:6**	1.1 ± 0.1	1.8 ± 0.3	/	3.3 ± 0.6	*	4.1 ± 0.4	**	2.60 ± 0.4	**	1.9 ± 0.4	/	3.8 ± 0.6	/	3.8 ± 0.4	/

	regular chow							semisynthetic diet							
	wt	$Ldlr^{-/-}$		$Mc4r^{mut}$		$Mc4r^{mut}/Ldlr^{-/-}$		wt		$Ldlr^{-/-}$		$Mc4r^{mut}$		$Mc4r^{mut}/Ldlr^{-/-}$	
	mean ± SEM	mean ± SEM	sign.	mean ± SEM	sign.	mean ± SEM	sign.	mean ± SEM	sign.	mean ± SEM	sign.	mean ± SEM	sign.	mean ± SEM	sign.
**Fatty acyl composition of liver triacylglycerols**															
**50:2**	6.6 ± 1.5	11.3 ± 1.7	/	11.6 ± 1.1	/	9.6 ± 0.5	/	12.9 ± 0.7	**	13.3 ± 1.2	/	10.6 ± 0.6	/	10.3 ± 1.4	/
**50:1**	1.5 ± 0.4	3.8 ± 0.7	/	7.0 ± 0.9	***	7.3 ± 1.01	***	12.6 ± 1.9	**	8.3 ± 1.0	/	10.0 ± 0.4	/	10.3 ± 1.5	/
**52:4**	17.6 ± 2.5	11.5 ± 2.4	/	4.0 ± 1.5	***	3.3 ± 1.6	***	0.8 ± 0.2	**	0.3 ± 0.1	/	0.2 ± 0.1	/	0.4 ± 0.1	/
**52:3**	43.7 ± 2.9	36.3 ± 2.4	/	26.0 ± 2.9	***	23.5 ± 2.7	***	14.5 ± 1.2	**	16.0 ± 0.9	/	12.8 ± 1.0	/	13.2 ± 0.7	/
**52:2**	21.4 ± 1.6	31.7 ± 3.1	/	45.8 ± 3.7	***	47.9 ± 2.9	***	51.0 ± 1.3	**	58.7 ± 3.3	/	56.7 ± 2.2	/	65.6 ± 3.7	/
**54:3**	9.1 ± 2.6	5.1 ± 1.5	/	5.2 ± 0.8	/	8.1 ± 2.2	/	8.0 ± 1.6	**	6.7 ± 1.1	/	9.4 ± 0.6	/	11.5 ± 0.9	/

For the total hepatic fat content median percentage is shown. All other Data are given as mean percentage ± SEM.

Significance with respect to wild-type mice of the respective diet was tested using a Kruskal-Wallis test. Additionally, the wild-type mice fed semisynthetic diet were compared to the regular chow fed wild-type littermates. (*) with p > 0,05 /, p <0.05 *, < 0.01 **, < 0.001 ***.

HFC - hepatic fat content
PC - phosphatidylcholine
PL - all phospholipids except phosphatidylcholine
PUFA - polyunsaturated fatty acid
MUFA - monounsaturated fatty acid
SAFA - saturated fatty acid
