# Supplementary material for: Altered hepatic lipid metabolism in mice lacking both the melanocortin type 4 receptor and low density lipoprotein receptor
Source: PLoS One. 2017 Feb 16;12(2):e0172000. doi: 10.1371/journal.pone.0172000 (PMC5313158; doi:10.1371/journal.pone.0172000)
Supplement: S3 Table — RNA-sequencing was performed from liver mRNA of the different mouse lines. Approximately 14 million reads per animal (10 mice per group) were analyzed. The total number of differentially expressed genes (as determined with DEseq) depending on the genotype and diet fed compared to the wild-type (wt) under regular chow is given. The overlap of differentially expressed genes between the groups is also shown in Fig 2. (PDF) [file pone.0172000.s006.pdf]

S3 Table. Summary of differentially expressed genes in liver derived from RNA sequence data.

	regular chow			semisynthetic diet			
	$Ldlr^{-/-}$	$Mc4r^{mut}$	$Mc4r^{mut};Ldlr^{-/-}$	wt	$Ldlr^{-/-}$	$Mc4r^{mut}$	$Mc4r^{mut};Ldlr^{-/-}$
# genes	19,883	20,010	20,515	20,278	20,263	20,257	20,478
# DE genes	868	1,278	1,254	2,015	1,905	2,649	2,853
# upregulated	377	640	821	869	1,125	1,435	2,101
# downregulated	491	638	433	1,146	780	1,214	752

DE = differentially expressed

p-value < 0.05 considered statistically significant
