# Supplementary material for: Altered hepatic lipid metabolism in mice lacking both the melanocortin type 4 receptor and low density lipoprotein receptor
Source: PLoS One. 2017 Feb 16;12(2):e0172000. doi: 10.1371/journal.pone.0172000 (PMC5313158; doi:10.1371/journal.pone.0172000)
Supplement: S4 Table — All KEGG pathways which showed a FDR < 0.05 (Correlation Adjusted Mean Rank Gene Set Test (CAMERA) from the R-package ‘limma’) were considered statistically significant. (PDF) [file pone.0172000.s007.pdf]

		regular chow									semisynthetic diet											
		$Ldlr^{-/-}$			$Mc4r^{mut}$			$Mc4r^{mut}$; $Ldlr^{-/-}$			wt			$Ldlr^{-/-}$			$Mc4r^{mut}$			$Mc4r^{mut}$; $Ldlr^{-/-}$		
**KEGG pathway**	**Ngenes**	**Direction**	**p-value**	**FDR**	**Direction**	**p-value**	**FDR**	**Direction**	**p-value**	**FDR**	**Direction**	**p-value**	**FDR**	**Direction**	**p-value**	**FDR**	**Direction**	**p-value**	**FDR**	**Direction**	**p-value**	**FDR**
CYTOSOLIC DNA SENSING PATHWAY	44	Up	9.89E-02	2.33E-01	Up	8.06E-02	1.90E-01	Up	4.49E-02	1.47E-01	Up	5.96E-03	3.58E-02	Up	7.90E-03	3.50E-02	Up	2.60E-03	1.03E-02	Up	1.38E-03	7.77E-03
STEROID HORMONE BIOSYNTHESIS	78	Down	8.38E-01	9.01E-01	Down	1.40E-02	6.04E-02	Down	1.43E-02	6.91E-02	Down	1.65E-06	3.07E-05	Down	1.77E-04	2.31E-03	Down	1.88E-03	7.76E-03	Down	7.42E-04	5.72E-03
GLYCEROLIPID METABOLISM	57	Up	1.83E-01	3.18E-01	Up	8.93E-04	7.55E-03	Up	1.72E-02	7.82E-02	Up	5.23E-03	3.48E-02	Up	4.77E-03	2.57E-02	Up	3.03E-04	2.56E-03	Up	6.21E-03	2.26E-02
LEISHMANIA INFECTION	99	Up	1.48E-01	2.96E-01	Up	3.12E-02	1.02E-01	Up	3.92E-03	3.64E-02	Up	6.16E-03	3.58E-02	Up	1.84E-04	2.31E-03	Up	2.42E-03	9.80E-03	Up	7.04E-04	5.72E-03
TOLL LIKE RECEPTOR SIGNALING PATHWAY	115	Up	7.67E-02	2.04E-01	Up	2.71E-02	9.52E-02	Up	6.40E-03	4.47E-02	Up	1.00E-03	9.79E-03	Up	9.73E-05	1.51E-03	Up	8.71E-04	5.05E-03	Up	3.76E-04	4.67E-03
LINOLEIC ACID METABOLISM	48	Down	2.13E-01	3.53E-01	Down	1.20E-03	9.69E-03	Down	6.48E-03	4.47E-02	Down	1.26E-12	2.34E-10	Down	3.42E-08	1.27E-06	Down	3.72E-07	1.38E-05	Down	3.54E-09	6.58E-07
PANTOTHENATE AND COA BIOSYNTHESIS	18	Up	1.29E-02	6.39E-02	Up	6.74E-03	4.32E-02	Up	5.53E-03	4.29E-02	Up	7.18E-03	3.82E-02	Up	3.08E-03	1.92E-02	Up	1.17E-03	5.89E-03	Up	1.55E-03	7.99E-03
PYRUVATE METABOLISM	61	Up	1.78E-02	7.10E-02	Up	1.21E-06	3.23E-05	Up	3.31E-03	3.64E-02	Up	8.31E-04	9.09E-03	Up	9.31E-04	7.53E-03	Up	8.40E-06	1.74E-04	Up	7.99E-04	5.72E-03
UBIQUITIN MEDIATED PROTEOLYSIS	142	Up	2.75E-04	3.66E-03	Up	1.17E-02	5.48E-02	Up	4.71E-03	3.98E-02	Up	8.82E-03	4.44E-02	Up	8.87E-04	7.50E-03	Up	7.82E-05	1.04E-03	Up	6.17E-05	1.64E-03
ALZHEIMERS DISEASE	181	Up	3.65E-06	9.71E-05	Up	3.60E-04	3.35E-03	Up	2.99E-05	7.94E-04	Up	2.56E-04	3.40E-03	Up	9.77E-05	1.51E-03	Up	1.01E-04	1.17E-03	Up	9.71E-04	6.01E-03
BIOSYNTHESIS OF UNSATURATED FATTY ACIDS	43	Up	3.45E-05	6.55E-04	Up	1.73E-10	3.22E-08	Up	7.09E-06	2.20E-04	Up	2.74E-07	1.02E-05	Up	7.37E-09	4.57E-07	Up	2.28E-08	1.42E-06	Up	1.44E-05	4.47E-04
CITRATE CYCLE TCA CYCLE	42	Up	3.52E-05	6.55E-04	Up	2.47E-05	3.53E-04	Up	9.79E-04	1.40E-02	Up	3.17E-03	2.27E-02	Up	1.99E-04	2.31E-03	Up	1.01E-05	1.89E-04	Up	1.29E-04	2.19E-03
GLYCEROPHOSPHOLIPID METABOLISM	63	Up	8.40E-03	4.59E-02	Up	8.02E-05	9.94E-04	Up	8.45E-04	1.31E-02	Up	8.73E-03	4.44E-02	Up	5.56E-03	2.80E-02	Up	9.88E-04	5.40E-03	Up	3.20E-03	1.42E-02
HUNTINGTONS DISEASE	189	Up	8.23E-09	3.83E-07	Up	6.48E-06	1.34E-04	Up	8.97E-08	4.17E-06	Up	4.49E-06	6.96E-05	Up	8.39E-06	1.95E-04	Up	2.36E-06	6.27E-05	Up	1.13E-05	4.21E-04
LYSOSOME	128	Up	2.86E-05	6.55E-04	Up	2.11E-05	3.45E-04	Up	4.84E-06	1.80E-04	Up	2.96E-03	2.20E-02	Up	3.93E-06	1.05E-04	Up	1.49E-07	6.92E-06	Up	3.50E-08	1.74E-06
OTHER GLYCAN DEGRADATION	18	Up	1.53E-03	1.59E-02	Up	4.33E-04	3.83E-03	Up	4.34E-04	8.08E-03	Up	3.34E-03	2.30E-02	Up	4.37E-03	2.54E-02	Up	2.47E-04	2.19E-03	Up	1.60E-04	2.47E-03
OXIDATIVE PHOSPHORYLATION	125	Up	8.23E-15	5.10E-13	Up	2.20E-09	2.05E-07	Up	2.22E-13	2.07E-11	Up	4.58E-10	2.84E-08	Up	7.81E-11	8.59E-09	Up	1.31E-09	1.69E-07	Up	3.74E-08	1.74E-06
PARKINSONS DISEASE	126	Up	3.12E-15	2.90E-13	Up	1.73E-08	8.05E-07	Up	7.37E-13	4.57E-11	Up	7.59E-10	3.53E-08	Up	9.24E-11	8.59E-09	Up	1.82E-09	1.69E-07	Up	1.83E-08	1.70E-06
PPAR SIGNALING PATHWAY	89	Up	6.93E-04	7.59E-03	Up	1.00E-08	6.22E-07	Up	2.31E-03	3.08E-02	Up	5.48E-07	1.70E-05	Up	4.41E-07	1.37E-05	Up	7.64E-06	1.74E-04	Up	2.32E-03	1.08E-02
PROTEASOME	43	Up	2.29E-06	7.09E-05	Up	1.99E-04	2.17E-03	Up	2.89E-04	5.97E-03	Up	1.99E-03	1.69E-02	Up	3.87E-03	2.32E-02	Up	3.87E-05	6.00E-04	Up	5.98E-04	5.57E-03
RIBOSOME	85	Up	1.47E-19	2.73E-17	Up	2.23E-05	3.45E-04	Up	1.47E-14	2.74E-12	Up	3.76E-10	2.84E-08	Up	1.60E-08	7.45E-07	Up	2.83E-05	4.78E-04	Up	1.11E-04	2.07E-03
VALINE LEUCINE AND ISOLEUCINE DEGRADATION	77	Up	4.71E-05	7.96E-04	Up	2.57E-08	9.55E-07	Up	2.75E-04	5.97E-03	Up	2.28E-03	1.76E-02	Up	8.25E-04	7.50E-03	Up	9.76E-07	3.03E-05	Up	5.50E-04	5.39E-03
ACUTE MYELOID LEUKEMIA	69	Up	4.59E-01	6.07E-01	Up	4.16E-02	1.17E-01	Up	1.60E-01	3.16E-01	Up	8.67E-02	2.07E-01	Up	8.79E-03	3.72E-02	Up	2.00E-02	4.97E-02	Up	1.44E-02	3.82E-02
APOPTOSIS	100	Up	3.07E-01	4.60E-01	Up	1.48E-01	2.84E-01	Up	2.11E-01	3.57E-01	Up	2.27E-01	3.87E-01	Up	4.58E-03	2.57E-02	Up	8.86E-03	2.75E-02	Up	6.47E-03	2.26E-02
CHEMOKINE SIGNALING PATHWAY	201	Up	4.52E-01	6.05E-01	Up	9.49E-02	2.15E-01	Up	2.42E-02	9.71E-02	Up	6.57E-02	1.78E-01	Up	6.97E-03	3.24E-02	Up	4.25E-03	1.61E-02	Up	1.75E-03	8.56E-03
COLORECTAL CANCER	73	Up	5.14E-02	1.47E-01	Up	1.50E-02	6.20E-02	Up	6.45E-02	1.74E-01	Up	2.65E-02	1.06E-01	Up	3.03E-03	1.92E-02	Up	5.68E-04	4.06E-03	Up	1.42E-03	7.78E-03
DRUG METABOLISM CYTOCHROME P450	110	Up	3.98E-02	1.24E-01	Up	4.90E-01	6.08E-01	Down	9.49E-01	9.75E-01	Down	2.94E-06	4.97E-05	Down	1.88E-04	2.31E-03	Down	2.90E-01	3.70E-01	Down	1.51E-02	3.92E-02
FC EPSILON RI SIGNALING PATHWAY	73	Up	8.78E-02	2.16E-01	Up	1.58E-01	2.91E-01	Up	1.40E-02	6.91E-02	Up	5.30E-02	1.56E-01	Up	6.45E-04	6.31E-03	Up	1.64E-03	7.44E-03	Up	4.77E-04	4.93E-03
GLYCOSAMINOGLYCAN DEGRADATION	25	Up	1.50E-01	2.96E-01	Up	1.28E-02	5.82E-02	Up	8.80E-03	5.45E-02	Up	2.86E-02	1.11E-01	Up	1.50E-03	1.12E-02	Up	5.18E-03	1.85E-02	Up	1.31E-03	7.59E-03
INSULIN SIGNALING PATHWAY	143	Up	2.03E-01	3.46E-01	Up	9.66E-02	2.16E-01	Up	1.87E-01	3.32E-01	Up	8.30E-02	2.03E-01	Up	6.65E-03	3.17E-02	Up	6.94E-03	2.30E-02	Up	7.54E-03	2.38E-02

		regular chow									semisynthetic diet											
		$Ldlr^{-/-}$			$Mc4r^{mut}$			$Mc4r^{mut}$; $Ldlr^{-/-}$			wt			$Ldlr^{-/-}$			$Mc4r^{mut}$			$Mc4r^{mut}$; $Ldlr^{-/-}$		
**KEGG pathway**	**Ngenes**	**Direction**	**p-value**	**FDR**	**Direction**	**p-value**	**FDR**	**Direction**	**p-value**	**FDR**	**Direction**	**p-value**	**FDR**	**Direction**	**p-value**	**FDR**	**Direction**	**p-value**	**FDR**	**Direction**	**p-value**	**FDR**
LYSINE DEGRADATION	71	Up	1.32E-02	6.39E-02	Up	1.06E-02	5.47E-02	Up	2.42E-02	9.71E-02	Up	4.00E-02	1.33E-01	Up	1.38E-02	4.95E-02	Up	8.05E-04	4.99E-03	Up	9.79E-03	2.89E-02
METABOLISM OF XENOBIOTICS BY CYTOCHROME P450	109	Up	6.15E-02	1.68E-01	Up	8.25E-01	8.62E-01	Down	6.19E-01	7.34E-01	Down	7.41E-07	1.97E-05	Down	4.95E-05	1.02E-03	Down	1.09E-01	1.72E-01	Down	5.23E-03	2.11E-02
MTOR SIGNALING PATHWAY	54	Up	8.55E-02	2.16E-01	Up	1.52E-01	2.89E-01	Up	3.12E-01	4.76E-01	Up	1.09E-01	2.42E-01	Up	1.19E-02	4.32E-02	Up	6.86E-03	2.30E-02	Up	1.21E-02	3.35E-02
NEUROTROPHIN SIGNALING PATHWAY	140	Up	8.78E-02	2.16E-01	Up	2.61E-02	9.35E-02	Up	5.40E-02	1.62E-01	Up	3.89E-02	1.31E-01	Up	1.01E-02	3.99E-02	Up	1.84E-03	7.76E-03	Up	8.34E-04	5.74E-03
NUCLEOTIDE EXCISION REPAIR	46	Up	1.74E-02	7.10E-02	Up	3.64E-02	1.09E-01	Up	1.36E-02	6.91E-02	Up	5.96E-03	3.58E-02	Up	4.51E-02	1.22E-01	Up	6.29E-04	4.33E-03	Up	4.35E-04	4.76E-03
P53 SIGNALING PATHWAY	89	Up	3.71E-02	1.21E-01	Up	8.65E-03	5.03E-02	Up	2.67E-02	9.93E-02	Up	6.54E-03	3.58E-02	Up	3.92E-02	1.10E-01	Up	7.33E-04	4.70E-03	Up	7.51E-03	2.38E-02
PANCREATIC CANCER	83	Up	7.95E-02	2.08E-01	Up	1.18E-02	5.48E-02	Up	5.90E-02	1.64E-01	Up	3.22E-02	1.18E-01	Up	8.24E-03	3.56E-02	Up	8.52E-04	5.05E-03	Up	1.49E-03	7.90E-03
RENAL CELL CARCINOMA	75	Up	2.85E-02	1.00E-01	Up	4.58E-02	1.22E-01	Up	6.22E-02	1.70E-01	Up	3.79E-02	1.31E-01	Up	4.36E-04	4.51E-03	Up	1.73E-03	7.60E-03	Up	8.99E-04	5.77E-03
RNA DEGRADATION	58	Up	2.33E-02	8.35E-02	Up	1.37E-01	2.69E-01	Up	2.30E-02	9.71E-02	Up	1.09E-02	5.33E-02	Up	1.16E-02	4.32E-02	Up	4.91E-04	3.65E-03	Up	7.29E-04	5.72E-03
T CELL RECEPTOR SIGNALING PATHWAY	126	Up	2.62E-01	4.20E-01	Up	2.39E-01	3.80E-01	Up	1.61E-01	3.16E-01	Up	6.30E-02	1.78E-01	Up	7.16E-03	3.25E-02	Up	1.78E-02	4.65E-02	Up	1.83E-03	8.75E-03
VEGF SIGNALING PATHWAY	72	Up	2.63E-01	4.20E-01	Up	1.54E-01	2.90E-01	Up	8.50E-02	2.14E-01	Up	1.07E-01	2.40E-01	Up	9.61E-03	3.89E-02	Up	1.84E-02	4.74E-02	Up	1.41E-02	3.82E-02
ALPHA LINOLENIC ACID METABOLISM	16	Up	1.26E-01	2.75E-01	Up	5.07E-03	3.37E-02	Up	7.97E-02	2.06E-01	Up	6.45E-03	3.58E-02	Up	1.13E-02	4.29E-02	Up	7.83E-03	2.56E-02	Up	6.24E-02	1.16E-01
B CELL RECEPTOR SIGNALING PATHWAY	104	Up	1.75E-02	7.10E-02	Up	1.12E-02	5.48E-02	Up	7.66E-04	1.30E-02	Up	3.72E-02	1.30E-01	Up	9.30E-05	1.51E-03	Up	1.56E-04	1.52E-03	Up	7.27E-05	1.69E-03
BUTANOATE METABOLISM	63	Up	2.09E-02	7.89E-02	Up	1.88E-05	3.45E-04	Up	3.04E-02	1.09E-01	Up	3.30E-02	1.18E-01	Up	1.05E-02	4.05E-02	Up	1.65E-04	1.54E-03	Up	6.69E-03	2.26E-02
ETHER LIPID METABOLISM	25	Up	3.83E-03	2.85E-02	Up	4.48E-02	1.21E-01	Up	1.23E-02	6.91E-02	Up	7.13E-02	1.82E-01	Up	3.10E-03	1.92E-02	Up	1.18E-02	3.45E-02	Up	6.56E-03	2.26E-02
FC GAMMA R MEDIATED PHAGOCYTOSIS	109	Up	4.09E-02	1.24E-01	Up	2.40E-02	8.91E-02	Up	3.65E-03	3.64E-02	Up	7.23E-02	1.82E-01	Up	1.74E-03	1.21E-02	Up	1.29E-03	6.13E-03	Up	2.38E-04	3.41E-03
SPLICEOSOME	165	Up	5.93E-03	3.80E-02	Up	9.93E-02	2.17E-01	Up	2.17E-02	9.63E-02	Up	1.30E-03	1.15E-02	Up	6.86E-02	1.56E-01	Up	1.14E-04	1.24E-03	Up	4.27E-04	4.76E-03
FATTY ACID METABOLISM	64	Up	2.14E-04	3.06E-03	Up	2.15E-07	6.68E-06	Up	1.21E-02	6.91E-02	Up	9.71E-04	9.79E-03	Up	2.11E-04	2.31E-03	Up	9.59E-05	1.17E-03	Up	3.27E-02	6.92E-02
OOCYTE MEIOSIS	107	Up	3.41E-02	1.15E-01	Up	4.96E-03	3.37E-02	Up	5.02E-03	4.06E-02	Up	2.26E-03	1.76E-02	Up	2.46E-02	7.49E-02	Up	8.97E-04	5.05E-03	Up	3.51E-03	1.52E-02
PROPANOATE METABOLISM	55	Up	2.67E-03	2.26E-02	Up	2.87E-06	6.68E-05	Up	1.05E-02	6.29E-02	Up	4.68E-02	1.45E-01	Up	1.23E-03	9.55E-03	Up	5.25E-05	7.50E-04	Up	5.70E-03	2.21E-02
PYRIMIDINE METABOLISM	103	Up	3.08E-04	3.81E-03	Up	1.10E-02	5.48E-02	Up	3.59E-03	3.64E-02	Up	5.78E-03	3.58E-02	Up	1.75E-02	6.15E-02	Up	3.27E-04	2.64E-03	Up	8.84E-05	1.83E-03
RNA POLYMERASE	28	Up	7.48E-03	4.35E-02	Up	1.72E-02	6.95E-02	Up	4.66E-03	3.98E-02	Up	5.42E-04	6.30E-03	Up	2.12E-02	6.79E-02	Up	1.63E-03	7.44E-03	Up	8.81E-04	5.77E-03
FOLATE BIOSYNTHESIS	10	Up	4.66E-04	5.42E-03	Up	2.84E-04	2.94E-03	Up	3.76E-03	3.64E-02	Up	1.67E-02	7.58E-02	Up	6.65E-03	3.17E-02	Up	5.94E-03	2.05E-02	Up	1.55E-02	3.95E-02
CELL CYCLE	125	Up	1.29E-01	2.79E-01	Up	3.31E-02	1.06E-01	Up	2.39E-02	9.71E-02	Up	1.17E-02	5.59E-02	Up	1.81E-01	3.12E-01	Up	1.11E-03	5.71E-03	Up	4.30E-03	1.78E-02
CHRONIC MYELOID LEUKEMIA	83	Up	3.39E-01	4.89E-01	Up	8.64E-03	5.03E-02	Up	1.14E-01	2.55E-01	Up	1.35E-01	2.82E-01	Up	2.70E-02	8.10E-02	Up	1.05E-03	5.58E-03	Up	1.00E-03	6.01E-03
DRUG METABOLISM OTHER ENZYMES	75	Up	4.28E-01	5.77E-01	Up	6.74E-01	7.55E-01	Up	7.25E-01	8.32E-01	Down	1.20E-04	1.72E-03	Down	8.85E-04	7.50E-03	Down	5.63E-01	6.59E-01	Down	3.66E-01	4.57E-01
EPITHELIAL CELL SIGNALING IN HELICOBACTER PYLORI INFECTION	88	Up	1.38E-01	2.86E-01	Up	2.31E-01	3.78E-01	Up	1.67E-01	3.18E-01	Up	6.81E-02	1.78E-01	Up	2.26E-02	7.12E-02	Up	1.65E-02	4.38E-02	Up	7.93E-03	2.42E-02
GALACTOSE METABOLISM	42	Up	2.98E-01	4.55E-01	Up	1.47E-02	6.20E-02	Up	1.44E-01	2.98E-01	Up	2.94E-02	1.12E-01	Up	1.50E-01	2.70E-01	Up	8.78E-03	2.75E-02	Up	1.52E-02	3.92E-02
GLIOMA	66	Up	5.01E-01	6.52E-01	Up	7.36E-02	1.80E-01	Up	1.41E-01	2.98E-01	Up	1.80E-01	3.38E-01	Up	6.79E-02	1.56E-01	Up	1.90E-02	4.84E-02	Up	9.08E-03	2.72E-02
LEUKOCYTE TRANSENDOTHELIAL MIGRATION	122	Up	5.25E-01	6.69E-01	Up	2.94E-01	4.48E-01	Up	1.66E-01	3.18E-01	Up	2.21E-01	3.82E-01	Up	5.56E-03	2.80E-02	Up	6.38E-02	1.16E-01	Up	1.43E-02	3.82E-02

		regular chow									semisynthetic diet											
		Ldlr$^{-/-}$			Mc4r^{mut}			Mc4r^{mut}; Ldlr$^{-/-}$			wt			Ldlr$^{-/-}$			Mc4r^{mut}			Mc4r^{mut}; Ldlr$^{-/-}$		
**KEGG pathway**	**Ngenes**	**Direction**	**p-value**	**FDR**	**Direction**	**p-value**	**FDR**	**Direction**	**p-value**	**FDR**	**Direction**	**p-value**	**FDR**	**Direction**	**p-value**	**FDR**	**Direction**	**p-value**	**FDR**	**Direction**	**p-value**	**FDR**
NATURAL KILLER CELL MEDIATED CYTOTOXICITY	132	Up	7.57E-01	8.41E-01	Up	1.88E-01	3.22E-01	Up	1.81E-01	3.27E-01	Up	6.62E-01	7.86E-01	Up	1.75E-03	1.21E-02	Up	6.33E-02	1.16E-01	Up	7.95E-03	2.42E-02
NITROGEN METABOLISM	20	Up	5.01E-01	6.52E-01	Down	3.74E-02	1.09E-01	Down	2.67E-01	4.24E-01	Down	2.96E-04	3.67E-03	Down	4.47E-02	1.22E-01	Down	1.76E-03	7.60E-03	Down	4.93E-02	9.72E-02
NON SMALL CELL LUNG CANCER	62	Up	2.10E-01	3.52E-01	Up	4.11E-02	1.17E-01	Up	9.68E-02	2.25E-01	Up	1.42E-01	2.91E-01	Up	1.89E-02	6.43E-02	Up	8.01E-03	2.57E-02	Up	1.03E-02	2.95E-02
PATHOGENIC ESCHERICHIA COLI INFECTION	87	Up	4.12E-02	1.24E-01	Up	3.00E-02	1.01E-01	Up	3.43E-02	1.20E-01	Up	5.15E-02	1.55E-01	Up	2.91E-02	8.46E-02	Up	1.19E-02	3.45E-02	Up	1.68E-03	8.46E-03
PENTOSE AND GLUCURONATE INTERCONVERSIONS	39	Up	8.91E-02	2.16E-01	Up	4.22E-02	1.17E-01	Up	5.53E-02	1.62E-01	Up	6.46E-01	7.85E-01	Up	4.08E-01	5.58E-01	Up	4.34E-03	1.61E-02	Up	1.80E-02	4.53E-02
PROGESTERONE MEDIATED OOCYTE MATURATION	84	Up	1.57E-01	2.99E-01	Up	1.76E-02	6.97E-02	Up	2.49E-02	9.71E-02	Up	1.97E-02	8.73E-02	Up	3.61E-02	1.03E-01	Up	9.97E-03	2.99E-02	Up	7.11E-03	2.32E-02
RETINOL METABOLISM	95	Up	1.78E-02	7.10E-02	Up	5.48E-01	6.50E-01	Up	9.26E-01	9.73E-01	Down	1.20E-06	2.79E-05	Down	1.93E-02	6.43E-02	Down	1.00E-01	1.63E-01	Down	6.39E-03	2.26E-02
RIBOFLAVIN METABOLISM	18	Up	2.04E-02	7.89E-02	Up	1.28E-01	2.59E-01	Up	5.90E-02	1.64E-01	Up	2.44E-01	4.03E-01	Up	9.44E-03	3.89E-02	Up	4.01E-02	8.10E-02	Up	1.18E-02	3.32E-02
SNARE INTERACTIONS IN VESICULAR TRANSPORT	36	Up	1.86E-01	3.21E-01	Up	7.48E-02	1.81E-01	Up	7.44E-02	1.95E-01	Up	1.65E-01	3.22E-01	Up	6.47E-02	1.52E-01	Up	9.21E-03	2.81E-02	Up	6.46E-03	2.26E-02
DNA REPLICATION	37	Up	4.25E-02	1.24E-01	Up	9.73E-02	2.16E-01	Up	6.05E-03	4.47E-02	Up	9.00E-02	2.12E-01	Up	6.11E-01	7.13E-01	Up	1.25E-03	6.10E-03	Up	6.54E-04	5.72E-03
GLYCOLYSIS GLUCONEOGENESIS	79	Up	1.00E-01	2.33E-01	Up	1.76E-04	2.04E-03	Up	2.56E-02	9.71E-02	Up	2.50E-02	1.06E-01	Up	5.47E-02	1.34E-01	Up	1.45E-04	1.50E-03	Up	5.69E-03	2.21E-02
PURINE METABOLISM	159	Up	8.19E-03	4.59E-02	Up	8.96E-02	2.06E-01	Up	1.45E-02	6.91E-02	Up	6.77E-02	1.78E-01	Up	7.24E-02	1.60E-01	Up	5.91E-03	2.05E-02	Up	7.78E-04	5.72E-03
SPHINGOLIPID METABOLISM	46	Up	8.13E-02	2.10E-01	Up	8.92E-03	5.03E-02	Up	3.91E-03	3.64E-02	Up	3.28E-02	1.18E-01	Up	2.08E-02	6.78E-02	Up	4.80E-04	3.65E-03	Up	3.40E-04	4.52E-03
PENTOSE PHOSPHATE PATHWAY	31	Up	1.77E-02	7.10E-02	Up	7.52E-03	4.67E-02	Up	7.14E-03	4.74E-02	Up	4.68E-02	1.45E-01	Up	1.90E-02	6.43E-02	Up	6.83E-04	4.54E-03	Up	4.02E-03	1.70E-02
ARACHIDONIC ACID METABOLISM	91	Up	1.62E-01	3.05E-01	Up	4.85E-01	6.08E-01	Down	5.53E-01	6.99E-01	Down	3.82E-01	5.59E-01	Down	2.46E-01	3.90E-01	Down	8.90E-02	1.48E-01	Down	2.88E-03	1.31E-02
BASAL CELL CARCINOMA	42	Down	1.77E-02	7.10E-02	Down	6.41E-01	7.32E-01	Down	1.38E-02	6.91E-02	Down	7.41E-01	8.34E-01	Down	7.20E-02	1.60E-01	Down	1.57E-02	4.22E-02	Down	3.20E-02	6.92E-02
BASAL TRANSCRIPTION FACTORS	32	Up	6.43E-02	1.73E-01	Up	7.72E-01	8.25E-01	Up	8.53E-02	2.14E-01	Up	8.43E-02	2.04E-01	Up	1.47E-01	2.67E-01	Up	2.74E-02	6.28E-02	Up	9.93E-03	2.89E-02
ERBB SIGNALING PATHWAY	86	Up	6.40E-01	7.68E-01	Up	1.64E-01	2.99E-01	Up	5.26E-01	6.84E-01	Up	2.22E-01	3.82E-01	Up	8.88E-02	1.84E-01	Up	4.31E-02	8.62E-02	Up	1.88E-02	4.61E-02
HOMOLOGOUS RECOMBINATION	27	Up	2.65E-01	4.20E-01	Up	2.48E-02	9.06E-02	Up	4.78E-02	1.53E-01	Up	4.19E-02	1.34E-01	Up	5.21E-01	6.55E-01	Up	1.45E-02	3.98E-02	Up	2.87E-02	6.48E-02
INTESTINAL IMMUNE NETWORK FOR IGA PRODUCTION	39	Up	9.13E-01	9.54E-01	Up	3.02E-01	4.53E-01	Up	1.74E-01	3.23E-01	Up	1.14E-01	2.47E-01	Up	4.84E-03	2.57E-02	Up	8.27E-02	1.40E-01	Up	5.27E-02	1.00E-01
RIG I LIKE RECEPTOR SIGNALING PATHWAY	61	Up	4.06E-01	5.60E-01	Up	1.26E-01	2.57E-01	Up	1.39E-01	2.98E-01	Up	1.45E-02	6.76E-02	Up	5.12E-02	1.31E-01	Up	1.31E-02	3.69E-02	Up	2.38E-02	5.57E-02
SMALL CELL LUNG CANCER	115	Up	7.88E-01	8.57E-01	Up	1.36E-01	2.69E-01	Up	9.41E-02	2.22E-01	Up	1.34E-01	2.82E-01	Up	1.72E-01	2.99E-01	Up	1.95E-02	4.91E-02	Up	2.34E-02	5.57E-02
STARCH AND SUCROSE METABOLISM	56	Up	1.38E-01	2.86E-01	Up	8.42E-02	1.96E-01	Up	3.79E-02	1.28E-01	Up	8.53E-01	8.91E-01	Up	3.31E-01	4.75E-01	Up	2.16E-02	5.28E-02	Up	1.85E-02	4.60E-02
STEROID BIOSYNTHESIS	20	Up	1.77E-01	3.18E-01	Up	2.86E-01	4.44E-01	Up	5.46E-01	6.96E-01	Down	1.38E-06	2.84E-05	Down	7.53E-01	8.19E-01	Down	1.84E-01	2.64E-01	Up	5.83E-01	6.46E-01
TERPENOID BACKBONE BIOSYNTHESIS	14	Up	5.77E-01	7.16E-01	Up	1.21E-01	2.51E-01	Up	9.77E-01	9.96E-01	Down	1.19E-03	1.11E-02	Down	9.77E-01	9.82E-01	Down	9.24E-01	9.51E-01	Up	4.31E-01	5.24E-01
AMINO SUGAR AND NUCLEOTIDE SUGAR METABOLISM	60	Up	7.28E-03	4.35E-02	Up	3.42E-02	1.08E-01	Up	1.93E-01	3.35E-01	Up	1.16E-01	2.48E-01	Up	5.03E-02	1.31E-01	Up	1.45E-02	3.98E-02	Up	3.67E-02	7.58E-02
BETA ALANINE METABOLISM	37	Up	2.33E-02	8.35E-02	Up	3.39E-04	3.32E-03	Up	3.79E-02	1.28E-01	Up	6.75E-02	1.78E-01	Up	5.18E-02	1.31E-01	Up	3.37E-03	1.31E-02	Up	3.32E-02	6.94E-02
LIMONENE AND PINENE DEGRADATION	18	Up	1.52E-01	2.96E-01	Up	3.28E-03	2.44E-02	Up	1.05E-01	2.37E-01	Up	2.53E-01	4.06E-01	Up	1.31E-01	2.52E-01	Up	1.20E-02	3.45E-02	Up	8.94E-02	1.52E-01
N GLYCAN BIOSYNTHESIS	45	Up	5.50E-03	3.79E-02	Up	4.33E-02	1.18E-01	Up	2.53E-02	9.71E-02	Up	5.39E-01	7.15E-01	Up	7.86E-02	1.70E-01	Up	2.61E-02	6.07E-02	Up	7.04E-03	2.32E-02
NEUROACTIVE LIGAND RECEPTOR INTERACTION	143	Down	1.87E-03	1.74E-02	Down	1.32E-02	5.85E-02	Down	8.44E-03	5.42E-02	Down	6.74E-02	1.78E-01	Down	4.13E-02	1.15E-01	Down	2.54E-02	5.99E-02	Down	6.35E-03	2.26E-02

		regular chow									semisynthetic diet											
		$Ldlr^{-/-}$			$Mc4r^{mut}$			$Mc4r^{mut}$; $Ldlr^{-/-}$			wt			$Ldlr^{-/-}$			$Mc4r^{mut}$			$Mc4r^{mut}$; $Ldlr^{-/-}$		
**KEGG pathway**	**Ngenes**	**Direction**	**p-value**	**FDR**	**Direction**	**p-value**	**FDR**	**Direction**	**p-value**	**FDR**	**Direction**	**p-value**	**FDR**	**Direction**	**p-value**	**FDR**	**Direction**	**p-value**	**FDR**	**Direction**	**p-value**	**FDR**
GLUTATHIONE METABOLISM	60	Up	2.10E-06	7.09E-05	Up	4.74E-05	6.29E-04	Up	1.63E-02	7.59E-02	Up	1.86E-01	3.46E-01	Up	5.83E-01	6.99E-01	Up	5.03E-03	1.83E-02	Up	2.96E-02	6.55E-02
ABC TRANSPORTERS	43	Up	5.91E-01	7.28E-01	Up	4.30E-01	5.83E-01	Up	5.91E-01	7.18E-01	Up	1.54E-01	3.08E-01	Up	1.60E-01	2.81E-01	Up	7.74E-02	1.32E-01	Up	1.89E-01	2.68E-01
ADHERENS JUNCTION	103	Up	6.61E-01	7.85E-01	Up	6.02E-01	6.96E-01	Down	8.43E-01	9.06E-01	Up	9.89E-01	9.89E-01	Up	2.20E-01	3.61E-01	Up	2.58E-01	3.44E-01	Up	2.47E-01	3.35E-01
ADIPOCYTOKINE SIGNALING PATHWAY	75	Up	3.61E-01	5.13E-01	Up	4.59E-01	6.08E-01	Up	8.22E-01	8.94E-01	Up	1.69E-01	3.24E-01	Up	1.18E-01	2.34E-01	Up	6.82E-02	1.22E-01	Up	2.27E-01	3.13E-01
ALANINE ASPARTATE AND GLUTAMATE METABOLISM	34	Up	2.12E-02	7.89E-02	Up	6.86E-01	7.64E-01	Up	5.78E-01	7.11E-01	Up	6.19E-01	7.73E-01	Up	3.37E-01	4.78E-01	Up	8.23E-01	8.70E-01	Up	7.61E-01	7.96E-01
ALDOSTERONE REGULATED SODIUM REABSORPTION	46	Up	5.54E-01	7.01E-01	Up	9.56E-01	9.62E-01	Up	3.30E-01	4.88E-01	Up	6.63E-01	7.86E-01	Up	2.07E-01	3.44E-01	Up	5.29E-01	6.26E-01	Up	7.47E-01	7.89E-01
AMINOACYL TRNA BIOSYNTHESIS	42	Up	3.86E-02	1.24E-01	Up	5.96E-02	1.52E-01	Up	1.89E-01	3.32E-01	Up	1.47E-01	2.96E-01	Up	5.65E-01	6.91E-01	Up	3.12E-02	6.76E-02	Up	2.40E-02	5.57E-02
AMYOTROPHIC LATERAL SCLEROSIS ALS	57	Up	1.75E-01	3.18E-01	Up	7.19E-01	7.87E-01	Up	3.69E-01	5.28E-01	Up	2.11E-01	3.70E-01	Up	2.45E-02	7.49E-02	Up	7.00E-02	1.24E-01	Up	1.33E-01	2.07E-01
ANTIGEN PROCESSING AND PRESENTATION	91	Down	5.11E-01	6.55E-01	Up	4.36E-01	5.83E-01	Up	8.43E-01	9.06E-01	Up	9.10E-01	9.41E-01	Up	4.19E-01	5.69E-01	Down	8.07E-01	8.58E-01	Up	9.53E-01	9.53E-01
ARGININE AND PROLINE METABOLISM	57	Up	3.62E-02	1.20E-01	Up	2.50E-01	3.91E-01	Up	7.42E-02	1.95E-01	Up	3.56E-01	5.26E-01	Up	6.87E-02	1.56E-01	Up	1.10E-01	1.72E-01	Up	2.03E-01	2.81E-01
ASCORBATE AND ALDARATE METABOLISM	28	Up	1.32E-01	2.82E-01	Up	1.58E-01	2.91E-01	Up	2.34E-01	3.82E-01	Down	1.97E-01	3.56E-01	Down	6.73E-01	7.55E-01	Up	2.07E-01	2.83E-01	Up	3.65E-01	4.57E-01
ASTHMA	18	Up	8.37E-01	9.01E-01	Up	4.71E-01	6.08E-01	Up	3.77E-01	5.35E-01	Up	2.67E-02	1.06E-01	Up	8.21E-02	1.76E-01	Up	6.20E-02	1.15E-01	Up	9.92E-02	1.63E-01
AXON GUIDANCE	136	Down	7.38E-01	8.32E-01	Up	9.30E-01	9.50E-01	Up	4.03E-01	5.60E-01	Down	9.37E-01	9.52E-01	Up	5.00E-01	6.46E-01	Up	7.83E-01	8.48E-01	Up	3.72E-01	4.61E-01
BASE EXCISION REPAIR	35	Up	1.08E-01	2.47E-01	Up	1.81E-01	3.18E-01	Up	1.43E-01	2.98E-01	Up	4.18E-02	1.34E-01	Up	6.86E-01	7.59E-01	Up	7.25E-02	1.27E-01	Up	2.89E-02	6.48E-02
BLADDER CANCER	51	Down	2.25E-01	3.67E-01	Up	7.31E-01	7.95E-01	Down	4.61E-01	6.17E-01	Up	6.20E-01	7.73E-01	Down	2.90E-01	4.39E-01	Up	2.74E-01	3.58E-01	Up	4.46E-01	5.31E-01
CARDIAC MUSCLE CONTRACTION	87	Up	2.71E-01	4.24E-01	Up	4.68E-01	6.08E-01	Up	2.10E-01	3.57E-01	Up	1.66E-01	3.22E-01	Down	9.47E-01	9.71E-01	Down	9.31E-01	9.51E-01	Down	4.18E-01	5.11E-01
CELL ADHESION MOLECULES CAMS	141	Down	1.79E-02	7.10E-02	Down	6.18E-01	7.09E-01	Down	1.57E-01	3.16E-01	Down	6.57E-02	1.78E-01	Down	8.62E-01	9.08E-01	Down	1.13E-01	1.75E-01	Down	8.39E-01	8.57E-01
CIRCADIAN RHYTHM MAMMAL	14	Up	9.99E-01	9.99E-01	Up	8.18E-01	8.59E-01	Down	4.99E-02	1.57E-01	Down	8.02E-01	8.74E-01	Up	8.33E-01	8.86E-01	Down	1.68E-01	2.45E-01	Down	1.40E-01	2.17E-01
COMPLEMENT AND COAGULATION CASCADES	83	Up	1.34E-01	2.84E-01	Down	1.34E-01	2.67E-01	Up	2.67E-01	4.24E-01	Down	2.06E-01	3.65E-01	Up	9.51E-01	9.71E-01	Down	9.52E-01	9.68E-01	Down	8.22E-01	8.45E-01
CYSTEINE AND METHIONINE METABOLISM	35	Up	1.57E-02	7.10E-02	Up	3.56E-01	5.18E-01	Up	9.38E-02	2.22E-01	Up	1.94E-01	3.56E-01	Up	1.00E-01	2.03E-01	Up	2.79E-01	3.58E-01	Up	1.62E-01	2.41E-01
CYTOKINE CYTOKINE RECEPTOR INTERACTION	184	Down	1.22E-01	2.74E-01	Down	3.77E-01	5.28E-01	Down	9.81E-01	9.96E-01	Down	6.08E-01	7.70E-01	Up	8.05E-01	8.61E-01	Up	9.69E-01	9.76E-01	Up	8.44E-01	8.58E-01
DILATED CARDIOMYOPATHY	102	Down	6.04E-02	1.68E-01	Down	2.16E-01	3.61E-01	Down	4.04E-01	5.60E-01	Down	5.30E-01	7.13E-01	Down	8.58E-02	1.79E-01	Down	1.69E-01	2.45E-01	Down	7.90E-02	1.40E-01
DORSO VENTRAL AXIS FORMATION	26	Up	3.48E-01	4.97E-01	Up	8.12E-01	8.58E-01	Up	8.48E-01	9.06E-01	Down	8.75E-01	9.09E-01	Up	6.22E-01	7.13E-01	Up	7.08E-01	7.95E-01	Up	5.67E-01	6.31E-01
ECM RECEPTOR INTERACTION	108	Down	4.24E-02	1.24E-01	Down	1.71E-01	3.09E-01	Down	7.20E-01	8.32E-01	Down	5.46E-01	7.15E-01	Down	1.37E-01	2.55E-01	Down	5.02E-01	5.99E-01	Down	4.15E-01	5.11E-01
ENDOCYTOSIS	213	Up	3.01E-01	4.56E-01	Up	3.60E-02	1.09E-01	Up	1.22E-01	2.70E-01	Up	7.67E-01	8.50E-01	Up	5.99E-02	1.45E-01	Up	8.42E-02	1.41E-01	Up	4.28E-02	8.66E-02
ENDOMETRIAL CANCER	59	Up	8.58E-01	9.12E-01	Up	5.13E-01	6.24E-01	Down	6.24E-01	7.35E-01	Up	6.98E-01	8.07E-01	Up	2.68E-01	4.15E-01	Up	2.78E-01	3.58E-01	Up	1.58E-01	2.37E-01
FOCAL ADHESION	248	Down	5.09E-01	6.55E-01	Down	8.12E-01	8.58E-01	Up	7.76E-01	8.64E-01	Up	9.35E-01	9.52E-01	Up	3.32E-01	4.75E-01	Up	3.63E-01	4.47E-01	Up	2.89E-01	3.81E-01
FRUCTOSE AND MANNOSE METABOLISM	71	Up	3.67E-01	5.17E-01	Up	4.64E-01	6.08E-01	Down	3.31E-01	4.88E-01	Up	9.67E-01	9.77E-01	Up	7.66E-01	8.28E-01	Up	1.40E-01	2.13E-01	Up	3.62E-01	4.57E-01
GAP JUNCTION	91	Down	8.46E-01	9.04E-01	Up	3.08E-01	4.58E-01	Up	7.67E-01	8.63E-01	Down	9.21E-01	9.46E-01	Up	9.55E-01	9.71E-01	Up	3.44E-01	4.30E-01	Up	2.78E-01	3.69E-01
GLYCOSAMINOGLYCAN BIOSYNTHESIS CHONDROITIN SULFATE	21	Up	7.35E-01	8.32E-01	Up	1.40E-01	2.70E-01	Up	2.96E-01	4.63E-01	Up	7.08E-01	8.13E-01	Up	4.02E-01	5.54E-01	Up	7.91E-01	8.48E-01	Up	8.18E-02	1.43E-01

		regular chow									semisynthetic diet											
		$Ldlr^{-/-}$			$Mc4r^{mut}$			$Mc4r^{mut}$; $Ldlr^{-/-}$			wt			$Ldlr^{-/-}$			$Mc4r^{mut}$			$Mc4r^{mut}$; $Ldlr^{-/-}$		
**KEGG pathway**	**Ngenes**	**Direction**	**p-value**	**FDR**	**Direction**	**p-value**	**FDR**	**Direction**	**p-value**	**FDR**	**Direction**	**p-value**	**FDR**	**Direction**	**p-value**	**FDR**	**Direction**	**p-value**	**FDR**	**Direction**	**p-value**	**FDR**
GLYCOSAMINOGLYCAN BIOSYNTHESIS HEPARAN SULFATE	20	Up	7.28E-01	8.31E-01	Up	9.52E-01	9.62E-01	Down	9.85E-01	9.96E-01	Down	6.48E-01	7.85E-01	Up	8.69E-01	9.08E-01	Down	7.37E-01	8.21E-01	Up	4.68E-01	5.50E-01
GLYCOSAMINOGLYCAN BIOSYNTHESIS KERATAN SULFATE	13	Down	7.60E-01	8.41E-01	Up	2.36E-01	3.80E-01	Up	1.24E-01	2.70E-01	Up	1.04E-01	2.35E-01	Up	6.24E-01	7.13E-01	Up	1.52E-01	2.26E-01	Up	1.74E-01	2.53E-01
GLYCOSPHINGOLIPID BIOSYNTHESIS GANGLIO SERIES	15	Up	2.05E-01	3.46E-01	Up	4.89E-01	6.08E-01	Up	1.03E-01	2.36E-01	Up	5.46E-01	7.15E-01	Up	2.40E-01	3.88E-01	Up	3.71E-01	4.55E-01	Up	1.97E-01	2.76E-01
GLYCOSPHINGOLIPID BIOSYNTHESIS GLOBO SERIES	12	Down	6.13E-01	7.41E-01	Up	8.58E-01	8.91E-01	Up	9.43E-01	9.75E-01	Down	6.78E-01	7.93E-01	Up	9.85E-01	9.85E-01	Up	7.93E-01	8.48E-01	Up	6.65E-01	7.15E-01
GLYCOSPHINGOLIPID BIOSYNTHESIS LACTO AND NEOLACTO SERIES	19	Down	9.96E-01	9.99E-01	Down	7.69E-01	8.25E-01	Up	4.33E-01	5.87E-01	Up	3.27E-01	4.86E-01	Up	7.80E-01	8.39E-01	Up	7.10E-01	7.95E-01	Up	4.39E-01	5.26E-01
GLYOXYLATE AND DICARBOXYLATE METABOLISM	20	Up	1.53E-01	2.96E-01	Up	1.05E-02	5.47E-02	Up	9.09E-02	2.21E-01	Up	2.60E-02	1.06E-01	Up	4.71E-02	1.25E-01	Up	4.71E-02	9.22E-02	Up	1.32E-01	2.07E-01
GNRH SIGNALING PATHWAY	98	Up	9.62E-01	9.88E-01	Up	5.11E-01	6.24E-01	Up	3.44E-01	5.03E-01	Up	1.64E-01	3.22E-01	Up	2.47E-01	3.90E-01	Up	2.00E-01	2.78E-01	Up	8.49E-02	1.46E-01
HEDGEHOG SIGNALING PATHWAY	47	Down	5.45E-02	1.54E-01	Down	9.12E-01	9.37E-01	Down	1.29E-02	6.91E-02	Down	4.43E-01	6.07E-01	Down	3.04E-01	4.52E-01	Down	7.53E-02	1.31E-01	Down	4.97E-02	9.72E-02
HEMATOPOIETIC CELL LINEAGE	92	Down	3.21E-01	4.67E-01	Down	5.90E-01	6.86E-01	Up	3.20E-01	4.84E-01	Down	6.86E-01	7.98E-01	Up	7.14E-01	7.82E-01	Down	9.30E-01	9.51E-01	Up	5.34E-01	5.99E-01
HISTIDINE METABOLISM	30	Up	1.44E-01	2.93E-01	Up	2.35E-02	8.91E-02	Up	9.14E-02	2.21E-01	Up	3.17E-01	4.83E-01	Up	3.08E-01	4.52E-01	Up	3.99E-02	8.10E-02	Up	7.34E-02	1.31E-01
HYPERTROPHIC CARDIOMYOPATHY HCM	94	Down	4.26E-02	1.24E-01	Down	1.06E-01	2.30E-01	Down	2.14E-01	3.59E-01	Down	2.52E-01	4.06E-01	Down	2.81E-02	8.30E-02	Down	9.06E-02	1.49E-01	Down	3.26E-02	6.92E-02
INOSITOL PHOSPHATE METABOLISM	52	Up	3.11E-01	4.62E-01	Up	2.28E-01	3.78E-01	Up	2.96E-01	4.63E-01	Up	2.66E-01	4.23E-01	Up	5.34E-02	1.33E-01	Up	2.20E-02	5.31E-02	Up	5.25E-02	1.00E-01
JAK STAT SIGNALING PATHWAY	112	Up	9.93E-01	9.99E-01	Down	6.90E-01	7.64E-01	Up	9.44E-01	9.75E-01	Up	4.19E-01	5.90E-01	Up	1.04E-01	2.08E-01	Up	5.77E-01	6.71E-01	Up	3.29E-01	4.26E-01
LONG TERM DEPRESSION	70	Down	4.06E-01	5.60E-01	Down	4.36E-01	5.83E-01	Down	5.33E-01	6.88E-01	Down	6.69E-01	7.88E-01	Down	9.05E-01	9.41E-01	Down	5.31E-01	6.26E-01	Down	4.78E-01	5.60E-01
LONG TERM POTENTIATION	77	Up	9.97E-01	9.99E-01	Down	4.81E-01	6.08E-01	Down	8.00E-01	8.81E-01	Up	7.73E-01	8.50E-01	Up	3.96E-01	5.51E-01	Up	6.17E-01	7.12E-01	Up	5.98E-01	6.55E-01
MAPK SIGNALING PATHWAY	257	Up	7.19E-01	8.26E-01	Up	1.77E-01	3.16E-01	Up	4.16E-01	5.69E-01	Up	7.38E-02	1.83E-01	Up	2.42E-01	3.88E-01	Up	1.09E-01	1.72E-01	Up	9.50E-02	1.58E-01
MATURITY ONSET DIABETES OF THE YOUNG	15	Down	9.06E-01	9.52E-01	Down	5.58E-01	6.55E-01	Down	2.34E-01	3.82E-01	Up	4.44E-01	6.07E-01	Down	5.89E-01	7.02E-01	Down	3.86E-01	4.69E-01	Down	2.93E-01	3.84E-01
MELANOGENESIS	96	Down	4.17E-01	5.66E-01	Up	4.28E-01	5.83E-01	Down	9.95E-01	9.97E-01	Up	8.18E-01	8.74E-01	Up	5.28E-01	6.55E-01	Up	8.77E-01	9.22E-01	Up	6.58E-01	7.12E-01
MELANOMA	70	Down	6.11E-01	7.41E-01	Up	3.74E-01	5.27E-01	Up	8.14E-01	8.91E-01	Up	7.70E-01	8.50E-01	Up	6.18E-01	7.13E-01	Up	1.82E-01	2.63E-01	Up	1.56E-01	2.37E-01
MISMATCH REPAIR	23	Up	3.13E-01	4.62E-01	Up	6.97E-01	7.67E-01	Up	1.66E-01	3.18E-01	Up	3.24E-01	4.85E-01	Up	6.78E-01	7.55E-01	Up	2.51E-02	5.99E-02	Up	8.21E-02	1.43E-01
NICOTINATE AND NICOTINAMIDE METABOLISM	27	Up	2.90E-01	4.46E-01	Up	1.11E-01	2.37E-01	Up	1.60E-01	3.16E-01	Up	4.16E-01	5.90E-01	Up	6.76E-01	7.55E-01	Up	6.13E-02	1.15E-01	Up	1.24E-01	1.97E-01
NOD LIKE RECEPTOR SIGNALING PATHWAY	77	Up	6.75E-01	7.95E-01	Up	4.07E-01	5.65E-01	Up	3.47E-01	5.04E-01	Up	1.97E-01	3.56E-01	Up	1.54E-01	2.74E-01	Up	5.51E-02	1.05E-01	Up	1.73E-01	2.53E-01
NON HOMOLOGOUS END JOINING	12	Down	6.83E-01	7.99E-01	Up	9.83E-01	9.83E-01	Up	7.64E-01	8.63E-01	Up	6.50E-01	7.85E-01	Down	9.76E-01	9.82E-01	Up	1.87E-01	2.65E-01	Up	1.73E-01	2.53E-01
NOTCH SIGNALING PATHWAY	46	Up	4.60E-01	6.07E-01	Up	4.93E-01	6.08E-01	Up	5.70E-01	7.11E-01	Up	7.35E-01	8.34E-01	Up	6.63E-01	7.52E-01	Down	9.12E-01	9.51E-01	Up	2.67E-01	3.58E-01
O GLYCAN BIOSYNTHESIS	23	Up	9.29E-01	9.65E-01	Down	4.93E-01	6.08E-01	Down	7.36E-01	8.40E-01	Down	2.71E-01	4.28E-01	Up	3.09E-01	4.52E-01	Up	7.91E-01	8.48E-01	Up	4.93E-01	5.66E-01
OLFACTORY TRANSDUCTION	36	Down	5.68E-01	7.10E-01	Down	2.44E-01	3.84E-01	Down	7.83E-01	8.67E-01	Down	6.23E-01	7.73E-01	Down	5.73E-01	6.96E-01	Down	3.04E-01	3.85E-01	Down	7.96E-01	8.23E-01
ONE CARBON POOL BY FOLATE	19	Up	1.78E-01	3.18E-01	Up	6.97E-02	1.75E-01	Up	8.80E-02	2.18E-01	Up	9.32E-02	2.14E-01	Up	6.00E-01	7.07E-01	Up	2.59E-01	3.44E-01	Up	2.41E-01	3.29E-01
PATHWAYS IN CANCER	352	Down	7.12E-01	8.22E-01	Up	1.83E-01	3.19E-01	Up	4.13E-01	5.69E-01	Up	2.93E-01	4.58E-01	Up	2.41E-01	3.88E-01	Up	1.07E-01	1.72E-01	Up	8.97E-02	1.52E-01
PHENYLALANINE METABOLISM	24	Up	1.55E-01	2.96E-01	Up	3.77E-02	1.09E-01	Up	4.72E-01	6.27E-01	Up	6.02E-01	7.67E-01	Up	4.75E-01	6.31E-01	Up	6.79E-01	7.80E-01	Up	6.43E-01	7.00E-01
PHOSPHATIDYLINOSITOL SIGNALING SYSTEM	68	Up	9.48E-01	9.80E-01	Up	5.60E-01	6.55E-01	Up	2.52E-01	4.08E-01	Up	6.35E-01	7.83E-01	Up	8.41E-02	1.78E-01	Up	2.78E-02	6.31E-02	Up	5.29E-02	1.00E-01

		regular chow									semisynthetic diet											
		Ldlr$^{-/-}$			Mc4r^{mut}			Mc4r^{mut}; Ldlr$^{-/-}$			wt			Ldlr$^{-/-}$			Mc4r^{mut}			Mc4r^{mut}; Ldlr$^{-/-}$		
**KEGG pathway**	**Ngenes**	**Direction**	**p-value**	**FDR**	**Direction**	**p-value**	**FDR**	**Direction**	**p-value**	**FDR**	**Direction**	**p-value**	**FDR**	**Direction**	**p-value**	**FDR**	**Direction**	**p-value**	**FDR**	**Direction**	**p-value**	**FDR**
PORPHYRIN AND CHLOROPHYLL METABOLISM	40	Up	3.19E-02	1.10E-01	Up	5.46E-02	1.43E-01	Up	5.50E-02	1.62E-01	Down	7.28E-01	8.30E-01	Up	4.79E-01	6.31E-01	Up	2.87E-02	6.36E-02	Up	2.18E-02	5.27E-02
PRIMARY BILE ACID BIOSYNTHESIS	80	Down	2.81E-01	4.35E-01	Down	5.43E-01	6.47E-01	Down	1.75E-01	3.23E-01	Down	4.23E-01	5.92E-01	Down	1.34E-01	2.54E-01	Down	2.07E-01	2.83E-01	Down	5.74E-02	1.08E-01
PRIMARY IMMUNODEFICIENCY	44	Down	1.24E-01	2.74E-01	Down	6.51E-01	7.39E-01	Up	5.86E-01	7.17E-01	Up	8.44E-01	8.87E-01	Down	3.05E-01	4.52E-01	Down	9.77E-01	9.77E-01	Up	5.95E-01	6.55E-01
PRION DISEASES	48	Up	1.69E-01	3.14E-01	Up	9.55E-01	9.62E-01	Down	9.97E-01	9.97E-01	Up	8.14E-01	8.74E-01	Up	8.69E-01	9.08E-01	Down	7.81E-01	8.48E-01	Down	7.17E-01	7.62E-01
PROSTATE CANCER	103	Up	6.63E-01	7.85E-01	Up	1.79E-01	3.18E-01	Up	8.57E-01	9.11E-01	Up	5.62E-01	7.25E-01	Up	3.66E-01	5.16E-01	Up	3.17E-01	3.99E-01	Up	3.27E-01	4.25E-01
PROXIMAL TUBULE BICARBONATE RECLAMATION	27	Up	1.10E-01	2.49E-01	Up	7.39E-01	7.99E-01	Up	1.95E-01	3.36E-01	Up	8.14E-01	8.74E-01	Up	5.27E-01	6.55E-01	Down	9.18E-01	9.51E-01	Down	7.00E-01	7.48E-01
REGULATION OF ACTIN CYTOSKELETON	227	Up	7.04E-01	8.19E-01	Up	2.98E-01	4.50E-01	Up	1.60E-01	3.16E-01	Up	3.03E-01	4.69E-01	Up	1.27E-01	2.46E-01	Up	4.60E-02	9.10E-02	Up	3.84E-02	7.84E-02
REGULATION OF AUTOPHAGY	21	Up	8.93E-02	2.16E-01	Up	1.14E-01	2.38E-01	Up	5.43E-01	6.96E-01	Up	3.12E-01	4.79E-01	Up	2.50E-01	3.90E-01	Up	6.53E-02	1.18E-01	Up	1.12E-01	1.79E-01
RENIN ANGIOTENSIN SYSTEM	15	Up	5.64E-01	7.09E-01	Up	4.63E-01	6.08E-01	Up	6.03E-01	7.22E-01	Up	8.13E-01	8.74E-01	Up	9.53E-01	9.71E-01	Up	7.49E-01	8.29E-01	Up	9.39E-01	9.50E-01
SELENOAMINO ACID METABOLISM	34	Up	1.45E-01	2.93E-01	Up	4.20E-01	5.79E-01	Up	3.30E-01	4.88E-01	Up	4.55E-01	6.17E-01	Up	5.23E-01	6.55E-01	Up	2.66E-01	3.51E-01	Up	1.58E-01	2.37E-01
SULFUR METABOLISM	11	Up	4.15E-01	5.66E-01	Down	8.63E-01	8.92E-01	Down	6.32E-01	7.39E-01	Down	6.54E-01	7.85E-01	Up	3.97E-01	5.51E-01	Down	4.21E-01	5.08E-01	Down	4.33E-01	5.24E-01
SYSTEMIC LUPUS ERYTHEMATOSUS	104	Down	3.72E-01	5.20E-01	Down	7.77E-02	1.85E-01	Down	5.76E-01	7.11E-01	Up	2.45E-01	4.03E-01	Down	2.83E-01	4.32E-01	Down	2.85E-02	6.36E-02	Down	1.44E-01	2.22E-01
TASTE TRANSDUCTION	46	Down	1.83E-01	3.18E-01	Down	4.84E-01	6.08E-01	Down	4.43E-01	5.96E-01	Down	8.39E-01	8.87E-01	Down	4.98E-01	6.46E-01	Down	7.63E-02	1.31E-01	Down	6.99E-02	1.28E-01
TAURINE AND HYPOTAURINE METABOLISM	10	Up	5.97E-01	7.31E-01	Up	1.13E-01	2.38E-01	Up	6.06E-01	7.22E-01	Down	1.79E-01	3.38E-01	Down	1.37E-01	2.55E-01	Down	9.71E-01	9.76E-01	Up	7.62E-01	7.96E-01
TGF BETA SIGNALING PATHWAY	82	Down	7.87E-01	8.57E-01	Up	6.70E-01	7.55E-01	Up	5.26E-01	6.84E-01	Up	6.90E-02	1.78E-01	Up	2.01E-01	3.40E-01	Up	5.15E-02	9.98E-02	Up	9.97E-02	1.63E-01
THYROID CANCER	37	Up	9.87E-01	9.99E-01	Up	3.08E-02	1.02E-01	Up	6.04E-01	7.22E-01	Up	9.17E-02	2.13E-01	Up	3.32E-01	4.75E-01	Up	3.60E-02	7.52E-02	Up	7.27E-02	1.31E-01
TIGHT JUNCTION	126	Up	7.53E-01	8.41E-01	Up	3.52E-01	5.16E-01	Up	5.64E-01	7.09E-01	Up	4.07E-01	5.88E-01	Up	4.90E-01	6.41E-01	Up	1.42E-01	2.15E-01	Up	1.83E-01	2.62E-01
TRYPTOPHAN METABOLISM	64	Up	1.34E-02	6.39E-02	Up	1.01E-02	5.47E-02	Up	1.44E-01	2.98E-01	Up	5.58E-01	7.25E-01	Up	2.76E-01	4.24E-01	Up	3.98E-02	8.10E-02	Up	2.55E-01	3.43E-01
TYPE II DIABETES MELLITUS	57	Down	2.70E-01	4.24E-01	Down	5.17E-01	6.25E-01	Down	6.47E-01	7.52E-01	Up	4.38E-01	6.07E-01	Up	4.39E-01	5.90E-01	Up	3.63E-01	4.47E-01	Up	5.03E-01	5.74E-01
VALINE LEUCINE AND ISOLEUCINE BIOSYNTHESIS	13	Up	1.81E-01	3.18E-01	Up	2.77E-02	9.53E-02	Up	5.66E-02	1.62E-01	Up	2.07E-02	8.94E-02	Up	1.95E-01	3.32E-01	Up	3.21E-02	6.87E-02	Up	2.74E-02	6.28E-02
VASCULAR SMOOTH MUSCLE CONTRACTION	113	Down	8.93E-01	9.43E-01	Up	5.32E-01	6.39E-01	Up	7.70E-01	8.63E-01	Up	5.82E-01	7.47E-01	Up	4.41E-01	5.90E-01	Up	7.57E-01	8.34E-01	Up	9.45E-01	9.50E-01
VASOPRESSIN REGULATED WATER REABSORPTION	56	Up	1.79E-01	3.18E-01	Up	5.75E-02	1.48E-01	Up	5.18E-02	1.61E-01	Up	5.85E-02	1.70E-01	Up	9.37E-02	1.91E-01	Up	1.55E-01	2.30E-01	Up	6.51E-02	1.20E-01
VIBRIO CHOLERAE INFECTION	69	Up	2.14E-01	3.53E-01	Up	1.87E-01	3.22E-01	Up	1.69E-01	3.18E-01	Up	4.08E-01	5.88E-01	Up	7.68E-02	1.68E-01	Up	5.38E-02	1.03E-01	Up	4.37E-02	8.74E-02
VIRAL MYOCARDITIS	86	Down	3.20E-01	4.67E-01	Up	2.14E-01	3.61E-01	Down	9.34E-01	9.75E-01	Down	8.43E-01	8.87E-01	Up	5.20E-02	1.31E-01	Up	6.93E-01	7.91E-01	Up	3.58E-01	4.56E-01
WNT SIGNALING PATHWAY	146	Down	7.84E-01	8.57E-01	Up	3.66E-01	5.24E-01	Down	8.81E-01	9.31E-01	Up	2.49E-01	4.06E-01	Up	5.25E-01	6.55E-01	Up	4.26E-01	5.12E-01	Up	4.61E-01	5.46E-01
ALLOGRAFT REJECTION	44	Down	2.46E-03	2.18E-02	Up	3.60E-01	5.19E-01	Down	4.00E-01	5.60E-01	Down	2.38E-01	4.00E-01	Up	5.10E-01	6.54E-01	Down	2.43E-01	3.27E-01	Down	4.91E-01	5.66E-01
ARRHYTHMOGENIC RIGHT VENTRICULAR CARDIOMYOPATHY [illegible]	83	Down	9.25E-03	4.92E-02	Down	7.36E-02	1.80E-01	Down	1.78E-01	3.25E-01	Down	1.38E-01	2.84E-01	Down	1.43E-01	2.64E-01	Down	1.37E-01	2.10E-01	Down	1.95E-01	2.75E-01
AUTOIMMUNE THYROID DISEASE	43	Down	5.77E-03	3.80E-02	Up	2.94E-01	4.48E-01	Down	3.02E-01	4.68E-01	Down	2.29E-01	3.87E-01	Up	5.32E-01	6.55E-01	Down	1.93E-01	2.72E-01	Down	4.84E-01	5.63E-01
CALCIUM SIGNALING PATHWAY	141	Down	1.32E-02	6.39E-02	Down	4.49E-03	3.21E-02	Down	4.42E-02	1.47E-01	Down	2.00E-01	3.57E-01	Down	2.03E-01	3.40E-01	Down	1.08E-01	1.72E-01	Down	1.09E-01	1.77E-01
GLYCINE SERINE AND THREONINE METABOLISM	38	Up	5.39E-03	3.79E-02	Up	3.73E-02	1.09E-01	Up	3.56E-01	5.14E-01	Down	9.79E-01	9.84E-01	Up	5.79E-01	6.99E-01	Up	6.98E-01	7.92E-01	Up	7.76E-01	8.06E-01

		regular chow									semisynthetic diet											
		**$Ldlr^{-/-}$**			**$Mc4r^{mut}$**			**$Mc4r^{mut}$; $Ldlr^{-/-}$**			**wt**			**$Ldlr^{-/-}$**			**$Mc4r^{mut}$**			**$Mc4r^{mut}$; $Ldlr^{-/-}$**		
**KEGG pathway**	**Ngenes**	**Direction**	**p-value**	**FDR**	**Direction**	**p-value**	**FDR**	**Direction**	**p-value**	**FDR**	**Direction**	**p-value**	**FDR**	**Direction**	**p-value**	**FDR**	**Direction**	**p-value**	**FDR**	**Direction**	**p-value**	**FDR**
GLYCOSYLPHOSPHATIDYLINOSITOL GPI ANCHOR BIOSYNTHESIS	25	Up	3.20E-03	2.58E-02	Up	2.32E-01	3.78E-01	Up	2.90E-02	1.06E-01	Up	1.14E-01	2.47E-01	Up	1.20E-01	2.35E-01	Up	3.37E-02	7.12E-02	Up	3.13E-02	6.86E-02
GRAFT VERSUS HOST DISEASE	47	Down	6.33E-03	3.92E-02	Up	3.23E-01	4.76E-01	Down	3.11E-01	4.76E-01	Down	5.33E-01	7.13E-01	Up	6.90E-01	7.59E-01	Down	1.98E-01	2.77E-01	Down	3.35E-01	4.29E-01
PEROXISOME	146	Up	9.67E-02	2.31E-01	Up	2.62E-03	2.03E-02	Up	5.65E-02	1.62E-01	Up	5.07E-02	1.54E-01	Up	6.22E-02	1.48E-01	Up	3.00E-02	6.56E-02	Up	1.77E-01	2.54E-01
PROTEIN EXPORT	28	Up	1.15E-04	1.78E-03	Up	2.37E-01	3.80E-01	Up	1.88E-01	3.32E-01	Up	4.17E-01	5.90E-01	Up	1.52E-01	2.72E-01	Up	1.44E-01	2.16E-01	Up	9.42E-02	1.58E-01
TYPE I DIABETES MELLITUS	53	Down	1.77E-03	1.73E-02	Up	3.74E-01	5.27E-01	Down	4.85E-01	6.39E-01	Down	3.19E-01	4.83E-01	Up	6.25E-01	7.13E-01	Down	2.40E-01	3.26E-01	Down	5.16E-01	5.82E-01
TYROSINE METABOLISM	57	Up	3.52E-03	2.72E-02	Up	2.06E-02	7.99E-02	Up	2.21E-01	3.68E-01	Up	7.45E-01	8.34E-01	Up	5.98E-01	7.07E-01	Up	2.76E-01	3.58E-01	Up	5.16E-01	5.82E-01
