# Supplementary material for: Altered hepatic lipid metabolism in mice lacking both the melanocortin type 4 receptor and low density lipoprotein receptor
Source: PLoS One. 2017 Feb 16;12(2):e0172000. doi: 10.1371/journal.pone.0172000 (PMC5313158; doi:10.1371/journal.pone.0172000)
Supplement: S5 Table — Differentially expression analysis results for all transcripts that were mentioned in the manuscript. The log2 fold expression change and the p-value for the comparison with the wt under regular chow is given. (PDF) [file pone.0172000.s008.pdf]

S5 Table. Expression changes of transcripts referred in text and figures.

	regular chow						semisynthetic diet									
	$Ldlr^{-/-}$		$Mc4r^{mut}$		$Mc4r^{mut}$; $Ldlr^{-/-}$		wt		$Ldlr^{-/-}$		$Mc4r^{mut}$		$Mc4r^{mut}$; $Ldlr^{-/-}$			
Ensembl Gene ID	log2 fold change	p-value	log2 fold change	p-value	log2 fold change	p-value	log2 fold change	p-value	log2 fold change	p-value	log2 fold change	p-value	log2 fold change	p-value	name	description
ENSMUSG00000035372	0.41	1.9E-01	0.79	6.8E-03	0.83	1.3E-02	1.08	1.6E-05	1.17	9.8E-05	0.96	3.4E-03	1.02	5.4E-03	1810055G02Rik	RIKEN cDNA 1810055G02 gene
ENSMUSG00000042041	1.01	1.5E-02	2.52	4.1E-06	2.23	2.2E-05	2.93	3.3E-06	2.46	9.6E-08	3.68	5.1E-17	3.67	2.4E-18	2010003K11Rik	RIKEN cDNA 2010003K11 gene
ENSMUSG00000025528	-5.81	1.6E-06	-3.73	3.1E-04	-3.88	3.6E-05	-1.31	4.6E-02	-3.43	2.9E-04	-5.41	8.0E-06	-Inf	1.4E-08	2010106E10Rik	RIKEN cDNA 2010106E10 gene
ENSMUSG00000086316	0.77	1.1E-02	1.34	1.8E-03	1.21	1.2E-03	1.33	4.6E-04	1.43	1.9E-04	1.71	5.0E-05	1.95	3.9E-06	2210013O21Rik	RIKEN cDNA 2210013O21 gene
ENSMUSG00000085642	-0.87	6.9E-03	-0.73	2.8E-02	-0.74	7.4E-03	-0.77	6.3E-03	-0.66	5.2E-03	-1.00	2.7E-03	-1.38	6.0E-04	3110053B16Rik	RIKEN cDNA 3110053B16 gene
ENSMUSG00000086299	0.88	1.7E-01	1.44	5.9E-04	1.96	1.2E-03	1.10	6.1E-03	1.55	3.8E-05	1.96	2.2E-05	2.32	2.0E-03	4732463B04Rik	RIKEN cDNA 4732463B04 gene
ENSMUSG00000036046	0.03	9.1E-01	0.71	7.4E-04	0.66	1.1E-02	0.70	5.1E-03	0.58	8.1E-03	0.88	5.2E-04	1.00	1.5E-03	5031439G07Rik	RIKEN cDNA 5031439G07 gene
ENSMUSG00000053168	-0.07	7.2E-01	1.08	8.1E-03	1.25	3.6E-03	1.21	3.5E-06	1.63	2.3E-03	2.45	6.9E-04	2.23	3.9E-03	9030619P08Rik	RIKEN cDNA 9030619P08 gene
ENSMUSG00000044060	-0.56	1.8E-02	-0.64	1.5E-02	-0.69	6.2E-03	-0.74	6.0E-03	-0.67	2.4E-04	-0.82	2.4E-03	-0.89	8.3E-03	A830010M20Rik	RIKEN cDNA A830010M20 gene
ENSMUSG00000087132	0.66	1.3E-01	0.73	4.3E-02	0.85	2.4E-02	0.75	2.5E-02	0.53	1.3E-01	1.07	4.2E-03	1.45	7.3E-04	A930001C03Rik	RIKEN cDNA A930001C03 gene
ENSMUSG00000035642	0.35	2.9E-02	0.50	1.3E-02	0.52	3.8E-02	0.55	3.6E-02	0.64	2.4E-03	0.48	1.4E-01	0.47	4.5E-01	Aamdc	adipogenesis associated Mth938 domain containing
ENSMUSG00000057191	0.72	2.3E-03	0.43	3.7E-02	1.41	9.3E-04	0.64	3.7E-03	1.51	2.2E-05	1.39	5.0E-04	2.55	3.1E-06	AB124611	cDNA sequence AB124611
ENSMUSG00000026944	-0.30	1.2E-01	-0.53	2.4E-03	-0.84	4.7E-04	-0.80	1.2E-05	-1.04	1.8E-06	-1.15	1.5E-04	-1.15	6.9E-04	Abca2	ATP-binding cassette, sub-family A (ABC1), member 2
ENSMUSG00000041828	-0.22	1.3E-01	-1.09	4.5E-05	-0.82	1.3E-02	-0.50	1.4E-02	-0.51	3.2E-02	-1.36	1.5E-06	-1.25	2.1E-05	Abca8a	ATP-binding cassette, sub-family A (ABC1), member 8a
ENSMUSG00000027048	-0.17	3.9E-01	-0.42	1.6E-02	-0.53	3.0E-02	-0.47	2.7E-02	-0.67	7.4E-04	-0.74	8.0E-03	-1.08	6.9E-04	Abcb11	ATP-binding cassette, sub-family B (MDR/TAP), member 11
ENSMUSG00000055782	1.27	2.4E-01	2.62	1.2E-03	3.58	6.7E-07	2.30	1.1E-04	3.06	1.2E-09	3.20	7.0E-21	3.81	2.4E-21	Abcd2	ATP-binding cassette, sub-family D (ALD), member 2
ENSMUSG00000036138	0.11	1.0E+00	0.89	2.3E-06	0.54	4.5E-02	0.60	1.7E-02	0.34	6.3E-02	0.74	7.4E-03	0.44	1.7E-01	Acaa1a	acetyl-Coenzyme A acyltransferase 1A
ENSMUSG00000010651	0.20	8.8E-01	1.14	3.5E-05	0.80	3.7E-03	0.76	6.9E-03	0.52	3.6E-03	1.04	2.0E-04	0.71	1.8E-02	Acaa1b	acetyl-Coenzyme A acyltransferase 1B
ENSMUSG00000020532	-0.10	2.0E-01	0.51	6.4E-01	0.52	2.3E-01	0.24	9.3E-01	1.02	6.3E-03	1.18	1.2E-02	1.53	1.4E-03	Acaca	acetyl-Coenzyme A carboxylase alpha
ENSMUSG00000042010	0.16	6.7E-01	0.72	1.8E-02	0.60	2.1E-01	0.47	4.6E-01	1.12	1.9E-04	0.79	1.4E-02	1.20	3.4E-03	Acacb	acetyl-Coenzyme A carboxylase beta
ENSMUSG00000029456	-0.09	4.9E-01	0.24	3.0E-01	0.12	7.9E-01	-0.01	6.8E-01	0.03	5.7E-01	0.12	4.7E-01	0.31	4.0E-01	Acad10	acyl-Coenzyme A dehydrogenase family, member 10
ENSMUSG00000090150	-0.11	2.4E-01	0.14	6.0E-01	0.08	9.4E-01	0.05	9.7E-01	0.00	1.0E+00	0.25	4.4E-01	0.00	8.5E-01	Acad11	acyl-Coenzyme A dehydrogenase family, member 11
ENSMUSG00000042647	-0.27	1.1E-01	0.27	2.0E-01	-0.06	8.7E-01	0.12	6.8E-01	0.08	6.2E-01	0.48	5.5E-02	0.60	5.1E-02	Acad12	acyl-Coenzyme A dehydrogenase family, member 12
ENSMUSG00000031969	-0.14	3.4E-01	0.06	7.6E-01	-0.35	1.4E-01	-0.04	8.2E-01	-0.37	1.5E-01	-0.22	3.5E-01	-0.48	8.9E-02	Acad8	acyl-Coenzyme A dehydrogenase family, member 8
ENSMUSG00000027710	0.13	6.0E-01	0.16	6.4E-01	0.31	1.7E-01	0.01	9.4E-01	0.10	3.6E-01	0.20	6.9E-01	0.19	3.7E-01	Acad9	acyl-Coenzyme A dehydrogenase family, member 9
ENSMUSG00000026003	0.17	7.3E-01	0.41	2.5E-02	0.41	9.2E-02	0.46	2.7E-02	0.42	2.8E-02	0.67	3.0E-02	0.47	1.3E-01	Acadl	acyl-Coenzyme A dehydrogenase, long-chain
ENSMUSG00000062908	0.18	6.0E-01	0.35	5.2E-02	0.36	1.2E-01	0.24	1.9E-01	0.15	3.4E-01	0.35	2.2E-01	0.15	7.0E-01	Acadm	acyl-Coenzyme A dehydrogenase, medium chain
ENSMUSG00000029545	0.03	9.9E-01	0.34	5.1E-02	0.07	6.6E-01	0.17	3.9E-01	0.08	3.7E-01	0.18	4.8E-01	0.03	8.0E-01	Acads	acyl-Coenzyme A dehydrogenase, short chain
ENSMUSG00000030861	-0.11	4.3E-01	-0.41	3.7E-02	-0.44	7.9E-02	-0.53	1.4E-02	-0.51	5.6E-03	-0.94	4.7E-04	-1.21	6.8E-05	Acadsb	acyl-Coenzyme A dehydrogenase, short/branched chain
ENSMUSG00000018574	0.05	8.7E-01	0.22	2.7E-01	0.05	9.3E-01	0.16	5.8E-01	-0.06	1.0E+00	0.17	6.2E-01	-0.15	6.7E-01	Acadvl	acyl-Coenzyme A dehydrogenase, very long chain
ENSMUSG00000072949	0.68	3.4E-01	1.12	1.6E-03	1.27	1.8E-02	0.98	3.4E-02	0.84	2.1E-02	0.92	6.2E-04	1.10	3.9E-02	Acot1	acyl-CoA thioesterase 1
ENSMUSG00000021228	1.97	8.3E-02	3.17	8.7E-05	4.48	2.3E-02	2.72	7.7E-06	3.26	5.1E-04	4.29	8.3E-04	4.98	1.7E-03	Acot3	acyl-CoA thioesterase 3
ENSMUSG00000052392	0.72	5.9E-02	1.28	1.5E-04	1.75	4.3E-06	0.95	6.0E-04	1.41	1.8E-05	1.72	9.2E-06	1.96	3.1E-04	Acot4	acyl-CoA thioesterase 4
ENSMUSG00000042540	1.56	1.8E-01	2.91	4.1E-02	2.65	2.0E-05	2.07	2.7E-02	1.90	6.7E-02	2.19	1.7E-02	3.29	2.7E-02	Acot5	acyl-CoA thioesterase 5

	regular chow						semisynthetic diet									
	Ldlr$^{-/-}$		Mc4r^{mut}		Mc4r^{mut}; Ldlr$^{-/-}$		wt		Ldlr$^{-/-}$		Mc4r^{mut}		Mc4r^{mut}; Ldlr$^{-/-}$			
**Ensembl Gene ID**	**log2 fold change**	**p-value**	**log2 fold change**	**p-value**	**log2 fold change**	**p-value**	**log2 fold change**	**p-value**	**log2 fold change**	**p-value**	**log2 fold change**	**p-value**	**log2 fold change**	**p-value**	**name**	**description**
ENSMUSG00000025287	0.36	1.1E-01	0.62	2.3E-02	1.01	6.2E-04	1.02	2.7E-03	0.95	6.2E-05	1.97	2.5E-02	1.93	1.5E-06	Acot9	acyl-CoA thioesterase 9
ENSMUSG00000032281	NA	NA	NA	NA	NA	NA	0.78	4.2E-01	NA	NA	NA	NA	0.40	7.1E-01	Acsbg1	acyl-CoA synthetase bubblegum family member 1
ENSMUSG00000076435	-0.06	7.4E-01	0.16	4.3E-01	0.08	8.3E-01	-0.32	1.0E-01	-0.25	3.6E-01	-0.29	5.3E-01	-0.02	9.8E-01	Acsf2	acyl-CoA synthetase family member 2
ENSMUSG00000015016	-0.02	9.9E-01	0.41	3.3E-02	0.08	7.8E-01	0.25	3.4E-01	0.13	3.2E-01	0.18	3.0E-01	0.28	2.8E-01	Acsf3	acyl-CoA synthetase family member 3
ENSMUSG00000018796	-0.39	5.7E-02	-0.21	3.5E-01	-0.42	9.2E-02	-0.29	1.8E-01	-0.40	1.0E-01	-0.67	4.2E-02	-0.52	7.7E-02	Acsl1	acyl-CoA synthetase long-chain family member 1
ENSMUSG00000032883	0.05	6.9E-01	0.19	9.2E-01	0.30	9.8E-02	-0.28	5.1E-01	0.33	1.1E-01	0.15	8.0E-01	0.40	1.6E-01	Acsl3	acyl-CoA synthetase long-chain family member 3
ENSMUSG00000031278	-0.19	6.9E-01	-0.49	7.1E-03	-0.48	7.2E-02	0.28	2.4E-01	0.09	6.5E-01	-0.15	5.0E-01	0.08	8.4E-01	Acsl4	acyl-CoA synthetase long-chain family member 4
ENSMUSG00000024981	0.30	3.5E-01	0.39	2.8E-01	0.44	1.6E-01	0.30	2.7E-01	0.41	5.3E-02	0.68	9.5E-02	0.59	1.0E-01	Acsl5	acyl-CoA synthetase long-chain family member 5
ENSMUSG00000020333	1.02	2.9E-01	0.06	9.4E-01	-2.36	1.2E-02	-1.51	3.1E-02	-0.20	8.0E-01	-5.24	1.1E-08	-4.03	6.2E-05	Acsl6	acyl-CoA synthetase long-chain family member 6
ENSMUSG00000033533	0.04	6.3E-01	-0.06	1.0E+00	-0.31	2.9E-01	-0.28	3.0E-01	-0.43	8.8E-02	-0.56	1.0E-01	-0.86	1.1E-02	Acsm1	acyl-CoA synthetase medium-chain family member 1
ENSMUSG00000030945	0.05	9.9E-01	-0.85	2.0E-01	-1.52	4.7E-02	-2.71	7.8E-03	-2.72	8.3E-04	-2.84	2.1E-03	-3.92	6.5E-04	Acsm2	acyl-CoA synthetase medium-chain family member 2
ENSMUSG00000030935	0.03	1.0E+00	0.04	6.5E-01	0.01	9.7E-01	0.30	9.4E-02	0.32	9.5E-02	0.22	4.6E-01	0.10	9.3E-01	Acsm3	acyl-CoA synthetase medium-chain family member 3
ENSMUSG00000030972	-0.16	2.0E-01	-0.21	1.0E-01	-0.24	2.4E-01	-0.42	1.8E-02	-0.55	4.4E-03	-0.26	4.0E-01	-0.59	3.9E-02	Acsm5	acyl-CoA synthetase medium-chain family member 5
ENSMUSG00000027452	0.18	7.7E-01	0.43	4.9E-01	0.07	4.9E-01	1.05	1.5E-01	0.46	2.4E-01	0.71	7.0E-02	1.78	1.3E-02	Acss1	acyl-CoA synthetase short-chain family member 1
ENSMUSG00000027605	0.01	6.3E-01	0.28	9.1E-01	0.14	9.0E-01	-0.40	2.7E-02	0.26	4.0E-01	-0.12	3.3E-01	0.18	8.8E-01	Acss2	acyl-CoA synthetase short-chain family member 2
ENSMUSG00000086529	NA	NA	NA	NA	NA	NA	NA	NA	0.29	3.5E-01	-0.08	3.9E-01	0.23	7.8E-01	Acss2os	acyl-CoA synthetase short-chain family member 2, opposite strand
ENSMUSG00000035948	0.48	6.3E-01	1.07	1.7E-02	1.22	1.2E-02	0.13	9.5E-01	0.75	2.4E-02	0.92	1.1E-02	0.90	2.1E-02	Acss3	acyl-CoA synthetase short-chain family member 3
ENSMUSG00000060923	0.33	9.8E-02	1.12	4.7E-04	1.08	6.0E-04	1.04	4.0E-04	0.53	1.4E-01	2.01	1.2E-01	1.64	8.1E-02	Acyp2	acylphosphatase 2, muscle type
ENSMUSG00000028041	0.42	7.0E-02	0.51	3.9E-02	0.58	1.9E-02	0.44	3.0E-02	0.43	7.4E-02	0.76	1.1E-02	1.04	2.2E-04	Adam15	a disintegrin and metallopeptidase domain 15 (metargidin)
ENSMUSG00000032363	-0.02	7.4E-01	-0.61	3.1E-03	-0.84	3.9E-04	-1.05	1.1E-07	-0.98	2.4E-05	-0.83	1.5E-02	-0.63	6.0E-02	Adamts7	a disintegrin-like and metallopeptidase (reprolysin type) with thrombospondin type 1 motif, 7
ENSMUSG00000020431	1.92	1.9E-02	2.03	1.8E-03	2.74	3.5E-04	2.37	1.9E-02	2.85	2.4E-04	2.35	8.5E-03	2.73	1.7E-06	Adcy1	adenylate cyclase 1
ENSMUSG00000041293	-0.72	5.5E-02	-2.54	8.7E-07	-2.23	2.4E-03	-1.02	3.0E-02	-1.58	8.4E-05	-5.18	9.7E-21	-5.64	1.3E-19	Adgrf1	adhesion G protein-coupled receptor F1
ENSMUSG00000069170	-0.85	2.0E-03	1.04	5.8E-05	-0.13	2.9E-01	1.62	1.8E-05	NA	NA	NA	NA	NA	NA	Adgrv1	adhesion G protein-coupled receptor V1
ENSMUSG00000029313	-0.40	1.6E-02	-0.34	4.4E-02	-0.50	4.1E-02	-0.44	1.4E-02	-0.41	2.8E-02	-0.42	2.1E-01	-0.23	6.4E-01	Aff1	AF4/FMR2 family, member 1
ENSMUSG00000034254	0.19	2.8E-01	0.41	8.6E-02	0.44	1.4E-01	0.56	4.1E-02	0.62	4.6E-02	0.68	1.0E-02	0.71	2.3E-02	Agpat1	1-acylglycerol-3-phosphate O-acyltransferase 1 (lysophosphatidic acid acyltransferase, alpha)
ENSMUSG00000026922	-0.08	8.0E-01	0.30	1.4E-01	0.05	4.7E-01	-0.15	5.8E-01	-0.02	5.4E-01	-0.19	5.1E-01	-0.26	5.4E-01	Agpat2	1-acylglycerol-3-phosphate O-acyltransferase 2 (lysophosphatidic acid acyltransferase, beta)
ENSMUSG00000001211	0.01	9.8E-01	0.23	2.0E-01	0.14	5.8E-01	0.26	2.2E-01	0.17	2.6E-01	0.31	2.1E-01	0.18	4.9E-01	Agpat3	1-acylglycerol-3-phosphate O-acyltransferase 3
ENSMUSG00000023827	0.50	5.0E-01	0.34	6.0E-01	0.91	1.6E-01	0.98	2.7E-02	1.39	2.6E-02	1.14	7.5E-02	1.50	9.5E-04	Agpat4	1-acylglycerol-3-phosphate O-acyltransferase 4 (lysophosphatidic acid acyltransferase, delta)
ENSMUSG00000031467	-0.05	7.3E-01	-0.19	4.7E-01	-0.09	9.8E-01	-0.16	6.4E-01	-0.12	6.1E-01	0.15	8.1E-01	0.03	9.4E-01	Agpat5	1-acylglycerol-3-phosphate O-acyltransferase 5 (lysophosphatidic acid acyltransferase, epsilon)
ENSMUSG00000029314	0.93	3.3E-03	1.73	1.5E-10	2.31	1.5E-06	1.84	3.2E-03	1.42	9.2E-04	2.48	2.4E-11	2.57	9.2E-13	Agpat9	1-acylglycerol-3-phosphate O-acyltransferase 9
ENSMUSG00000012123	0.18	1.0E+00	0.53	4.5E-02	1.50	2.6E-04	1.58	2.7E-05	2.04	3.2E-07	2.24	3.8E-09	2.61	2.9E-09	Aim1l	absent in melanoma 1-like
ENSMUSG00000066406	-0.36	8.5E-02	-0.60	3.1E-03	-0.42	4.5E-02	-0.68	4.4E-03	-0.56	4.4E-03	-0.55	2.0E-02	-0.30	1.7E-01	Akap13	A kinase (PRKA) anchor protein 13
ENSMUSG00000038729	-0.12	1.7E-01	0.48	4.1E-01	0.45	3.3E-01	0.33	4.5E-01	0.25	7.8E-01	0.97	8.2E-02	0.72	3.5E-02	Akap2	A kinase (PRKA) anchor protein 2
ENSMUSG00000010025	0.03	5.1E-01	1.01	6.5E-03	0.77	1.1E-02	0.80	1.3E-03	0.46	2.2E-02	1.35	4.7E-06	0.97	2.2E-03	Aldh3a2	aldehyde dehydrogenase family 3, subfamily A2
ENSMUSG00000072115	0.07	2.2E-01	-0.71	2.7E-01	-0.30	5.3E-01	-0.34	6.5E-01	-0.68	2.5E-02	-1.14	1.4E-03	-1.80	1.4E-06	Ang	angiogenin, ribonuclease, RNase A family, 5

	regular chow						semisynthetic diet									
	$Ldlr^{-/-}$		$Mc4r^{mut}$		$Mc4r^{mut}$; $Ldlr^{-/-}$		wt		$Ldlr^{-/-}$		$Mc4r^{mut}$		$Mc4r^{mut}$; $Ldlr^{-/-}$			
**Ensembl Gene ID**	**log2 fold change**	**p-value**	**log2 fold change**	**p-value**	**log2 fold change**	**p-value**	**log2 fold change**	**p-value**	**log2 fold change**	**p-value**	**log2 fold change**	**p-value**	**log2 fold change**	**p-value**	**name**	**description**
ENSMUSG00000022237	-0.26	2.8E-01	-0.76	9.9E-05	-0.62	3.1E-02	-1.02	4.0E-07	-0.57	9.5E-03	-1.25	6.1E-06	-1.23	2.9E-04	Ankrd33b	ankyrin repeat domain 33B
ENSMUSG00000032231	0.27	1.2E-01	1.46	5.3E-03	1.49	2.0E-03	1.69	3.2E-03	1.14	2.0E-03	2.87	3.7E-09	2.81	3.7E-09	Anxa2	annexin A2
ENSMUSG00000027712	0.27	8.7E-02	1.14	1.3E-03	0.88	3.5E-03	0.81	1.2E-02	0.53	1.0E-03	1.83	8.0E-08	1.89	3.2E-09	Anxa5	annexin A5
ENSMUSG00000064294	-0.36	4.4E-02	-0.65	4.2E-04	-0.87	4.5E-03	-1.10	4.5E-07	-1.52	5.1E-14	-1.64	6.1E-09	-2.15	3.5E-11	Aox3	aldehyde oxidase 3
ENSMUSG00000032080	0.38	1.9E-01	2.43	2.8E-02	2.71	4.5E-04	3.67	3.9E-08	3.76	2.9E-20	4.98	6.4E-15	4.89	3.7E-28	Apoa4	apolipoprotein A-IV
ENSMUSG00000002992	0.50	5.7E-03	0.82	1.2E-02	1.00	3.2E-05	1.11	3.1E-07	1.20	2.1E-09	1.72	1.3E-02	1.63	4.3E-04	Apoc2	apolipoprotein C-II
ENSMUSG00000024391	0.20	1.5E-01	-0.50	1.3E-02	-0.56	3.1E-02	-0.51	2.9E-02	-0.44	7.3E-02	-1.08	1.5E-04	-1.30	3.6E-05	Apom	apolipoprotein M
ENSMUSG00000041219	0.12	2.8E-01	1.27	1.9E-04	1.65	4.8E-04	1.22	2.1E-03	0.95	7.0E-03	1.71	3.7E-05	2.03	4.9E-07	Arhgap11a	Rho GTPase activating protein 11A
ENSMUSG00000050730	-0.24	1.4E-01	-0.60	4.1E-03	-0.63	2.0E-02	-0.50	1.2E-02	-0.38	6.2E-02	-0.86	2.1E-03	-0.96	2.6E-03	Arhgap42	Rho GTPase activating protein 42
ENSMUSG00000051517	0.86	3.8E-01	1.79	1.5E-02	2.24	1.0E-02	1.74	1.7E-02	1.95	1.5E-01	2.63	9.3E-03	2.38	4.9E-03	Arhgef39	Rho guanine nucleotide exchange factor (GEF) 39
ENSMUSG00000002910	-0.44	1.6E-01	-0.56	2.1E-02	-0.49	2.9E-02	-1.09	7.6E-05	-0.89	2.3E-03	-0.83	2.9E-03	-0.95	8.5E-03	Arrdc2	arrestin domain containing 2
ENSMUSG00000038539	0.07	3.7E-01	0.67	1.4E-04	0.47	3.2E-02	0.45	4.8E-02	0.08	2.3E-01	0.27	1.5E-01	0.36	6.1E-02	Atf5	activating transcription factor 5
ENSMUSG00000062949	-0.30	2.9E-02	-0.59	4.6E-04	-0.56	1.4E-02	-0.55	8.6E-03	-0.42	2.9E-02	-0.79	4.2E-03	-0.75	4.6E-03	Atp11c	ATPase, class VI, type 11C
ENSMUSG00000030302	1.60	1.4E-03	2.22	1.6E-03	2.89	6.7E-05	1.45	2.9E-02	2.32	1.7E-05	2.66	1.6E-02	3.28	2.3E-04	Atp2b2	ATPase, Ca++ transporting, plasma membrane 2
ENSMUSG00000039529	-0.26	2.9E-01	-0.42	5.5E-03	-0.56	3.4E-02	-0.47	2.5E-02	-0.34	1.7E-01	-0.75	7.1E-03	-0.64	3.1E-02	Atp8b1	ATPase, class I, type 8B, member 1
ENSMUSG00000054843	-0.20	2.0E-01	-0.45	1.1E-02	-0.50	2.6E-02	-0.61	2.1E-03	-0.39	4.2E-02	-0.60	1.3E-02	-0.55	7.7E-02	Atrnl1	attractin like 1
ENSMUSG00000020123	-0.42	1.1E-01	-1.24	1.5E-06	-1.56	2.2E-05	-0.98	9.0E-04	-1.61	6.9E-10	-2.42	1.4E-12	-2.58	8.1E-11	Avpr1a	arginine vasopressin receptor 1A
ENSMUSG00000046415	-0.38	6.3E-01	3.88	3.3E-04	3.67	2.0E-02	3.29	4.7E-04	2.29	1.6E-01	4.89	7.4E-07	4.05	7.5E-05	B430212C06Rik	RIKEN cDNA B430212C06 gene
ENSMUSG00000022641	-0.41	2.2E-02	-0.50	9.4E-04	-0.57	1.6E-02	-0.46	2.1E-02	-0.29	1.0E-01	-0.39	1.6E-01	-0.27	2.4E-01	Bbx	bobby sox homolog (Drosophila)
ENSMUSG00000074649	0.09	6.7E-01	0.71	3.7E-03	0.57	2.5E-02	0.53	2.1E-02	0.30	1.3E-01	1.16	5.4E-04	0.91	8.0E-03	BC029722	cDNA sequence BC029722
ENSMUSG00000027792	0.23	9.7E-01	0.26	4.2E-01	0.68	1.0E-02	-0.14	4.2E-01	0.41	2.1E-01	0.49	2.4E-01	0.64	1.5E-01	Bche	butyrylcholinesterase
ENSMUSG00000034663	0.19	3.8E-01	0.62	4.7E-02	0.59	4.6E-02	0.64	1.1E-02	0.61	3.4E-02	0.79	1.9E-02	0.78	5.6E-02	Bmp2k	BMP2 inducible kinase
ENSMUSG00000021835	-1.22	7.5E-05	-0.91	4.6E-02	-0.68	3.9E-02	-0.87	8.9E-03	-0.59	1.2E-01	-0.93	2.3E-02	-0.67	1.7E-01	Bmp4	bone morphogenetic protein 4
ENSMUSG00000031963	2.56	9.8E-02	1.64	2.5E-02	2.40	9.8E-06	1.23	1.7E-03	2.11	5.0E-03	1.91	2.4E-05	2.72	4.0E-07	Bmper	BMP-binding endothelial regulator
ENSMUSG00000040084	0.60	2.0E-01	1.00	2.3E-02	1.73	3.5E-02	1.32	1.5E-02	1.30	1.7E-02	2.07	7.4E-05	2.38	2.1E-04	Bub1b	BUB1B, mitotic checkpoint serine/threonine kinase
ENSMUSG00000036887	0.30	4.4E-02	0.48	2.5E-02	0.69	3.5E-02	0.55	3.9E-03	0.65	2.7E-04	0.88	1.5E-03	1.22	7.8E-05	C1qa	complement component 1, q subcomponent, alpha polypeptide
ENSMUSG00000036905	0.57	3.7E-03	0.52	4.6E-02	0.92	6.6E-03	0.59	2.9E-03	0.92	2.3E-06	1.00	3.3E-04	1.57	1.1E-06	C1qb	complement component 1, q subcomponent, beta polypeptide
ENSMUSG00000036896	0.51	8.4E-03	0.49	1.7E-02	0.86	1.4E-02	0.56	4.8E-03	0.87	1.8E-06	0.95	4.7E-04	1.37	8.4E-06	C1qc	complement component 1, q subcomponent, C chain
ENSMUSG00000055172	-0.19	2.9E-01	-0.44	2.6E-02	-0.45	4.7E-02	-0.37	6.1E-02	-0.38	1.0E-01	-0.55	5.7E-02	-0.71	3.2E-02	C1ra	complement component 1, r subcomponent A
ENSMUSG00000092005	-0.30	4.3E-02	-0.49	9.2E-03	-0.46	3.7E-02	-0.54	3.0E-03	-0.36	5.6E-02	-0.53	4.3E-02	-0.64	4.1E-02	C1rb	complement component 1, r subcomponent B
ENSMUSG00000035031	-0.27	3.5E-01	-0.82	1.3E-04	-0.75	2.9E-03	-0.80	6.1E-04	-1.03	3.3E-07	-1.56	4.5E-08	-1.81	6.3E-08	C8a	complement component 8, alpha polypeptide
ENSMUSG00000029656	-0.30	4.0E-01	-1.22	1.4E-05	-1.26	1.9E-04	-0.79	8.9E-03	-1.23	2.5E-07	-2.53	4.8E-16	-2.90	1.7E-16	C8b	complement component 8, beta polypeptide
ENSMUSG00000038599	-0.84	5.7E-02	-1.34	1.1E-02	-2.67	2.1E-05	-2.15	2.2E-04	-2.11	3.0E-06	-4.12	1.4E-12	-4.15	7.2E-12	Capn8	calpain 8
ENSMUSG00000018930	0.61	7.2E-01	1.27	7.4E-01	2.90	2.3E-01	1.51	2.4E-01	2.72	7.0E-02	4.28	2.1E-01	3.76	1.4E-04	Ccl4	chemokine (C-C motif) ligand 4
ENSMUSG00000035042	0.06	3.3E-01	0.14	2.8E-01	2.16	2.4E-01	1.00	6.4E-02	0.85	1.0E-01	1.39	1.0E-02	1.86	4.0E-05	Ccl5	chemokine (C-C motif) ligand 5

	regular chow						semisynthetic diet									
	Ldlr$^{-/-}$		Mc4r^{mut}		Mc4r^{mut}; Ldlr$^{-/-}$		wt		Ldlr$^{-/-}$		Mc4r^{mut}		Mc4r^{mut}; Ldlr$^{-/-}$			
**Ensembl Gene ID**	**log2 fold change**	**p-value**	**log2 fold change**	**p-value**	**log2 fold change**	**p-value**	**log2 fold change**	**p-value**	**log2 fold change**	**p-value**	**log2 fold change**	**p-value**	**log2 fold change**	**p-value**	**name**	**description**
ENSMUSG00000079227	0.11	4.8E-01	-0.05	8.0E-01	0.69	5.8E-02	0.11	8.2E-01	0.85	1.8E-01	1.08	2.0E-02	1.58	7.0E-03	Ccr5	chemokine (C-C motif) receptor 5
ENSMUSG00000026012	0.35	7.1E-01	0.67	9.9E-01	0.51	7.5E-01	0.09	9.0E-01	0.89	7.6E-01	-0.69	7.9E-01	1.51	5.4E-01	Cd28	CD28 antigen
ENSMUSG00000002944	0.80	3.3E-02	1.72	3.0E-04	2.22	2.5E-07	1.25	2.1E-03	1.83	9.0E-07	2.60	2.8E-07	2.77	3.6E-15	Cd36	CD36 antigen
ENSMUSG00000029084	0.34	5.7E-02	0.08	8.2E-01	0.49	5.6E-02	-0.18	3.4E-01	0.56	2.1E-01	0.27	3.5E-01	1.02	1.4E-02	Cd38	CD38 antigen
ENSMUSG00000023274	-1.53	5.8E-03	-2.13	1.1E-04	-2.60	8.2E-05	-1.24	3.3E-02	-2.07	1.7E-05	-3.06	1.3E-06	-2.60	1.8E-04	Cd4	CD4 antigen
ENSMUSG00000018774	0.47	2.7E-02	0.62	9.2E-03	1.24	1.6E-03	0.85	8.4E-04	1.15	2.5E-05	1.58	3.3E-08	2.18	6.6E-10	Cd68	CD68 antigen
ENSMUSG00000040592	0.04	5.3E-01	0.48	1.6E-01	0.67	8.4E-02	0.29	2.2E-01	1.34	2.1E-01	1.21	1.6E-02	1.32	1.0E-03	Cd79b	CD79B antigen
ENSMUSG00000022901	1.16	2.5E-07	0.55	3.7E-02	1.66	2.3E-04	0.75	8.7E-03	1.70	9.7E-12	1.50	1.4E-01	2.10	4.3E-06	Cd86	CD86 antigen
ENSMUSG00000028755	-0.06	5.0E-01	0.79	3.7E-04	0.66	3.2E-02	0.78	1.2E-03	0.21	9.6E-02	0.71	5.0E-03	0.91	2.3E-03	Cda	cytidine deaminase
ENSMUSG00000037628	NA	NA	3.36	1.6E-02	4.57	1.1E-03	4.59	4.1E-03	3.87	8.1E-02	5.48	3.9E-04	5.76	1.5E-05	Cdkn3	cyclin-dependent kinase inhibitor 3
ENSMUSG00000023031	-0.20	2.5E-01	-0.95	9.4E-05	-1.36	1.4E-08	-0.82	2.5E-04	-0.90	4.6E-06	-1.47	1.2E-08	-1.35	5.0E-05	Cela1	chymotrypsin-like elastase family, member 1
ENSMUSG00000061825	-0.65	4.7E-03	-0.90	2.0E-04	-1.06	5.5E-04	-1.20	1.0E-05	-1.50	1.5E-08	-1.15	8.1E-06	-1.45	1.1E-06	Ces2c	carboxylesterase 2C
ENSMUSG00000031884	-0.69	7.7E-03	-0.72	1.3E-02	-0.94	6.2E-03	-0.94	1.9E-03	-1.48	9.6E-06	-0.96	4.4E-03	-1.24	2.0E-04	Ces2d-ps	carboxylesterase 2D, pseudogene
ENSMUSG00000069922	-0.49	8.8E-04	-0.49	5.7E-03	-0.78	7.6E-05	-0.50	2.8E-03	-0.79	1.5E-05	-0.94	3.7E-04	-1.62	1.5E-08	Ces3a	carboxylesterase 3A
ENSMUSG00000020462	0.51	2.7E-01	0.43	2.9E-02	0.56	1.5E-02	0.55	7.0E-03	0.60	1.1E-02	0.52	6.4E-01	0.37	6.2E-01	Cfap36	cilia and flagella associated protein 36
ENSMUSG00000001128	0.62	5.0E-03	0.49	5.6E-03	0.97	2.1E-03	0.44	1.8E-02	1.13	3.7E-05	0.75	4.4E-03	1.46	1.2E-05	Cfp	complement factor properdin
ENSMUSG00000029161	0.53	2.6E-01	1.80	2.9E-04	1.84	9.4E-03	2.61	8.1E-04	2.31	2.3E-07	3.78	1.3E-11	3.57	1.2E-13	Cgref1	cell growth regulator with EF hand domain 1
ENSMUSG00000030086	0.22	2.7E-01	0.95	1.5E-03	0.90	3.2E-03	0.63	7.2E-03	0.47	4.4E-02	1.11	1.4E-02	1.21	3.5E-02	Chchd6	coiled-coil-helix-coiled-coil-helix domain containing 6
ENSMUSG00000004843	0.33	5.8E-02	0.46	3.5E-02	0.45	2.1E-02	0.53	2.6E-03	0.53	2.3E-02	0.93	5.2E-03	0.97	3.4E-03	Chmp2b	charged multivesicular body protein 2B
ENSMUSG00000032997	0.47	9.2E-02	0.35	2.8E-02	0.67	6.6E-03	0.74	8.8E-03	0.65	8.8E-02	0.65	2.9E-02	0.97	2.5E-03	Chpf	chondroitin polymerizing factor
ENSMUSG00000060002	0.11	7.3E-01	0.56	6.7E-04	0.50	2.2E-02	0.13	2.2E-01	0.27	2.9E-01	0.33	2.4E-01	0.25	7.4E-01	Chpt1	choline phosphotransferase 1
ENSMUSG00000024526	0.53	7.7E-01	6.88	1.1E-09	6.41	6.2E-03	5.68	1.3E-04	3.96	3.2E-02	7.95	8.8E-11	6.43	7.7E-07	Cidea	cell death-inducing DNA fragmentation factor, alpha subunit-like effector A
ENSMUSG00000022219	0.10	6.4E-01	0.41	2.7E-02	0.17	4.6E-01	0.41	5.5E-02	0.24	1.0E-01	0.28	2.0E-01	0.04	7.8E-01	Cideb	cell death-inducing DNA fragmentation factor, alpha subunit-like effector B
ENSMUSG00000030278	0.56	6.7E-01	3.71	2.2E-06	3.08	5.0E-05	3.82	5.5E-05	2.33	9.6E-04	4.45	2.6E-32	3.95	4.9E-17	Cidec	cell death-inducing DFFA-like effector c
ENSMUSG00000039910	-0.53	5.9E-02	-0.52	1.9E-02	-0.97	1.3E-04	-0.74	2.7E-04	-0.92	2.7E-04	-0.88	7.2E-03	-1.14	1.9E-03	Cited2	Cbp/p300-interacting transactivator, with Glu/Asp-rich carboxy-terminal domain, 2
ENSMUSG00000037725	0.43	6.8E-01	2.40	4.3E-02	3.31	4.4E-04	3.32	2.4E-02	2.23	6.8E-02	3.66	5.6E-04	4.04	6.0E-07	Ckap2	cytoskeleton associated protein 2
ENSMUSG00000030159	0.60	1.3E-04	0.16	4.5E-02	0.95	1.3E-04	0.61	7.0E-04	1.18	3.5E-04	1.15	5.0E-02	1.80	2.6E-02	Clec1b	C-type lectin domain family 1, member b
ENSMUSG00000030148	0.45	1.7E-01	0.84	2.4E-02	1.21	4.8E-02	1.01	4.4E-03	1.28	2.0E-02	2.06	4.4E-03	2.34	1.5E-02	Clec4a2	C-type lectin domain family 4, member a2
ENSMUSG00000029238	-0.28	6.8E-02	-0.45	8.4E-03	-0.45	4.8E-02	-0.64	5.6E-03	-0.67	2.5E-04	-1.00	1.7E-05	-1.02	7.2E-04	Clock	circadian locomotor output cycles kaput
ENSMUSG00000090110	0.13	4.3E-01	0.38	4.5E-02	0.52	3.1E-02	0.57	1.2E-02	0.45	6.3E-02	0.68	5.8E-02	0.48	1.8E-01	Cmc4	C-x(9)-C motif containing 4
ENSMUSG00000028719	0.17	4.4E-01	0.20	2.0E-01	0.19	1.6E-01	0.19	1.4E-01	0.20	3.7E-01	0.56	3.5E-01	0.39	4.2E-01	Cmpk1	cytidine monophosphate (UMP-CMP) kinase 1
ENSMUSG00000006782	-0.19	3.1E-01	-0.41	2.8E-02	-0.54	8.8E-03	-0.43	1.6E-02	-0.26	3.3E-01	-0.67	1.4E-02	-0.36	2.3E-01	Cnp	2',3'-cyclic nucleotide 3' phosphodiesterase
ENSMUSG00000018672	0.10	3.2E-01	0.70	1.9E-04	0.52	1.1E-02	0.54	6.7E-03	0.43	1.3E-02	0.72	1.8E-03	0.71	1.2E-02	Copz2	coatomer protein complex, subunit zeta 2
ENSMUSG00000005220	1.95	3.3E-01	3.48	4.1E-02	4.30	2.2E-03	2.51	2.2E-03	2.16	1.8E-02	3.93	3.0E-06	4.85	5.8E-11	Corin	corin
ENSMUSG00000025991	-0.30	8.6E-02	-1.07	5.1E-07	-0.86	1.5E-04	-0.54	1.9E-03	-0.47	2.4E-02	-1.14	2.9E-04	-1.14	2.2E-04	Cps1	carbamoyl-phosphate synthetase 1

	regular chow						semisynthetic diet									
	Ldlr$^{-/-}$		Mc4r^{mut}		Mc4r^{mut}; Ldlr$^{-/-}$		wt		Ldlr$^{-/-}$		Mc4r^{mut}		Mc4r^{mut}; Ldlr$^{-/-}$			
**Ensembl Gene ID**	**log2 fold change**	**p-value**	**log2 fold change**	**p-value**	**log2 fold change**	**p-value**	**log2 fold change**	**p-value**	**log2 fold change**	**p-value**	**log2 fold change**	**p-value**	**log2 fold change**	**p-value**	**name**	**description**
ENSMUSG00000024900	-0.03	7.8E-01	0.18	2.1E-01	0.31	3.6E-01	0.56	1.6E-02	0.75	2.2E-02	0.67	4.3E-03	0.85	7.7E-03	Cpt1a	carnitine palmitoyltransferase 1a, liver
ENSMUSG00000078937	0.06	7.6E-01	0.09	9.7E-01	0.17	9.6E-01	0.21	6.7E-01	-0.31	1.5E-01	0.64	7.8E-02	0.34	3.2E-01	Cpt1b	carnitine palmitoyltransferase 1b, muscle
ENSMUSG00000007783	0.06	8.1E-01	0.75	7.5E-01	0.59	8.9E-01	1.23	2.9E-01	1.28	4.8E-01	1.40	1.6E-01	1.30	1.5E-01	Cpt1c	carnitine palmitoyltransferase 1c
ENSMUSG00000028607	0.05	1.0E+00	0.29	6.1E-02	0.13	6.4E-01	0.15	4.3E-01	0.02	7.8E-01	0.10	5.6E-01	0.02	8.9E-01	Cpt2	carnitine palmitoyltransferase 2
ENSMUSG00000029073	NA	NA	NA	NA	NA	NA	NA	NA	-0.11	6.1E-01	-0.05	8.9E-01	-0.05	9.0E-01	Cptp	ceramide-1-phosphate transfer protein
ENSMUSG00000026853	0.34	1.1E-01	1.43	2.2E-07	1.38	2.2E-05	0.94	1.1E-02	0.77	2.3E-04	1.36	2.5E-07	1.48	1.5E-06	Crat	carnitine acetyltransferase
ENSMUSG00000022521	-0.38	4.3E-02	-0.43	2.1E-02	-0.50	1.1E-02	-0.41	3.7E-02	-0.56	4.9E-03	-0.45	3.9E-02	-0.50	1.6E-01	Crebbp	CREB binding protein
ENSMUSG00000006360	0.54	9.8E-03	0.50	1.8E-02	1.06	3.0E-03	0.94	1.1E-03	1.28	1.3E-03	1.32	6.1E-03	1.97	1.8E-04	Crip1	cysteine-rich protein 1 (intestinal)
ENSMUSG00000059326	0.31	1.7E-01	0.35	2.6E-01	0.72	1.8E-01	0.74	2.6E-02	0.70	9.9E-03	1.08	6.3E-04	1.81	1.3E-06	Csf2ra	colony stimulating factor 2 receptor, alpha, low-affinity (granulocyte-macrophage)
ENSMUSG00000071713	0.37	1.0E-01	0.49	1.6E-01	0.92	5.0E-02	0.40	1.4E-01	1.09	7.1E-05	0.89	2.7E-03	1.74	4.0E-06	Csf2rb	colony stimulating factor 2 receptor, beta, low-affinity (granulocyte-macrophage)
ENSMUSG00000071714	0.54	1.5E-01	0.61	2.2E-01	0.93	2.6E-01	0.16	7.8E-01	1.01	4.8E-02	1.19	2.6E-03	2.44	3.8E-05	Csf2rb2	colony stimulating factor 2 receptor, beta 2, low-affinity (granulocyte-macrophage)
ENSMUSG00000005054	0.31	9.2E-02	0.38	5.8E-02	0.48	2.3E-02	0.49	9.0E-03	0.41	2.5E-02	1.12	6.4E-02	1.35	1.4E-02	Cstb	cystatin B
ENSMUSG00000070495	0.15	5.4E-01	0.64	8.4E-03	0.81	2.0E-02	0.54	4.0E-02	0.44	2.6E-01	0.58	6.3E-02	0.49	1.6E-01	Ctcfl	CCCTC-binding factor (zinc finger protein)-like
ENSMUSG00000087382	0.23	2.7E-01	0.58	5.8E-03	0.85	1.8E-03	0.66	4.5E-03	0.52	3.6E-01	0.65	2.6E-02	0.46	7.7E-02	Ctcflos	CCCTC-binding factor (zinc finger protein)-like, opposite strand
ENSMUSG00000034855	-0.47	9.9E-01	0.01	6.1E-01	0.93	1.3E-01	0.76	4.2E-02	0.46	3.6E-02	1.41	2.3E-02	1.23	4.4E-04	Cxcl10	chemokine (C-X-C motif) ligand 10
ENSMUSG00000045382	0.14	4.2E-01	0.89	7.5E-02	1.53	2.2E-02	1.21	2.0E-02	1.47	5.5E-04	1.50	6.1E-03	2.59	2.7E-07	Cxcr4	chemokine (C-X-C motif) receptor 4
ENSMUSG00000024560	-0.23	3.5E-01	-0.50	1.6E-02	-0.54	2.1E-02	-0.48	2.1E-02	-0.60	1.5E-02	-0.95	5.2E-03	-0.68	6.1E-02	Cxxc1	CXXC finger 1 (PHD domain)
ENSMUSG00000003555	1.95	9.7E-02	1.69	1.2E-02	2.79	1.8E-04	1.71	3.1E-04	3.19	6.6E-21	2.78	2.3E-07	3.56	5.3E-16	Cyp17a1	cytochrome P450, family 17, subfamily a, polypeptide 1
ENSMUSG00000032315	-0.99	1.9E-03	-0.87	6.6E-03	-1.05	1.4E-03	-1.51	8.1E-06	-1.28	4.9E-04	-1.32	1.7E-04	-1.62	1.4E-04	Cyp1a1	cytochrome P450, family 1, subfamily a, polypeptide 1
ENSMUSG00000032310	-0.41	1.4E-02	-1.03	1.3E-08	-1.22	5.2E-07	-1.30	2.4E-10	-1.51	3.7E-12	-1.90	5.5E-11	-2.33	2.1E-13	Cyp1a2	cytochrome P450, family 1, subfamily a, polypeptide 2
ENSMUSG00000026170	-0.18	5.1E-01	-0.08	9.7E-01	-0.50	6.1E-02	-0.28	1.3E-01	-0.44	4.9E-02	-0.49	2.4E-01	-0.97	4.8E-03	Cyp27a1	cytochrome P450, family 27, subfamily a, polypeptide 1
ENSMUSG00000042248	-0.01	3.4E-01	-1.07	1.8E-04	-0.95	7.8E-04	-1.33	2.6E-04	-1.23	3.3E-05	-1.84	2.2E-07	-2.39	6.9E-11	Cyp2c37	cytochrome P450, family 2. subfamily c, polypeptide 37
ENSMUSG00000054827	-0.11	1.2E-01	-0.70	2.6E-05	-0.90	3.5E-07	-1.18	5.1E-07	-1.17	1.7E-08	-1.79	1.2E-12	-2.50	1.1E-18	Cyp2c50	cytochrome P450, family 2, subfamily c, polypeptide 50
ENSMUSG00000067225	-0.22	1.3E-01	-1.32	2.1E-03	-1.36	4.4E-04	-1.44	2.8E-03	-1.35	1.2E-03	-2.89	5.1E-07	-3.41	1.6E-08	Cyp2c54	cytochrome P450, family 2, subfamily c, polypeptide 54
ENSMUSG00000060613	-0.34	6.4E-01	-1.29	2.0E-06	-1.36	6.7E-06	-0.86	6.4E-03	-0.96	6.7E-05	-1.76	6.3E-09	-2.06	1.6E-09	Cyp2c70	cytochrome P450, family 2, subfamily c, polypeptide 70
ENSMUSG00000014372	-0.40	3.6E-03	-0.35	2.5E-02	-0.43	3.0E-03	-0.36	6.2E-03	-0.55	2.6E-03	-0.66	5.6E-03	-0.98	3.6E-04	Cyp2d10	cytochrome P450, family 2, subfamily d, polypeptide 10
ENSMUSG00000068085	-1.37	2.6E-03	-0.99	4.8E-02	-0.90	7.6E-03	-0.80	9.5E-03	-1.13	6.9E-03	-1.80	7.6E-04	-1.66	1.2E-03	Cyp2d11	cytochrome P450, family 2, subfamily d, polypeptide 11
ENSMUSG00000068086	-0.56	6.2E-04	-0.82	3.0E-05	-1.38	1.1E-10	-0.64	1.1E-04	-1.11	4.9E-09	-1.49	9.5E-09	-2.07	5.1E-13	Cyp2d9	cytochrome P450, family 2, subfamily d, polypeptide 9
ENSMUSG00000052914	-0.16	8.1E-02	-0.46	1.6E-03	-0.36	3.3E-02	-0.37	1.7E-02	-0.20	7.7E-02	-0.22	7.0E-02	-0.26	1.1E-01	Cyp2j6	cytochrome P450, family 2, subfamily j, polypeptide 6
ENSMUSG00000027983	-0.27	1.0E-01	-0.46	4.4E-02	-1.05	1.6E-03	-0.49	2.1E-02	-0.88	7.6E-04	-0.97	2.0E-03	-1.79	4.5E-08	Cyp2u1	cytochrome P450, family 2, subfamily u, polypeptide 1
ENSMUSG00000021259	1.13	8.4E-02	2.01	1.9E-02	2.16	8.1E-03	1.94	1.5E-02	2.85	2.9E-04	2.75	1.4E-05	3.56	1.9E-05	Cyp46a1	cytochrome P450, family 46, subfamily a, polypeptide 1
ENSMUSG00000066072	2.21	2.1E-03	2.25	1.5E-04	2.64	2.6E-03	1.81	8.6E-04	2.51	2.7E-08	2.18	1.0E-09	2.48	2.2E-05	Cyp4a10	cytochrome P450, family 4, subfamily a, polypeptide 10
ENSMUSG00000066071	0.08	9.5E-01	-0.03	6.8E-01	-0.88	6.6E-03	-0.15	2.5E-01	-0.39	2.8E-02	-1.17	1.9E-04	-1.15	1.6E-04	Cyp4a12a	cytochrome P450, family 4, subfamily a, polypeptide 12a
ENSMUSG00000078597	0.20	5.0E-01	-0.03	1.0E+00	-0.94	2.1E-02	0.12	7.8E-01	0.04	7.5E-01	-1.08	4.3E-03	-1.18	8.8E-04	Cyp4a12b	cytochrome P450, family 4, subfamily a, polypeptide 12B
ENSMUSG00000028715	3.58	9.6E-05	3.99	1.0E-09	5.11	5.9E-05	3.80	7.4E-06	4.14	7.2E-10	4.31	6.0E-34	4.76	5.5E-14	Cyp4a14	cytochrome P450, family 4, subfamily a, polypeptide 14

	regular chow						semisynthetic diet									
	Ldlr$^{-/-}$		Mc4r^{mut}		Mc4r^{mut}; Ldlr$^{-/-}$		wt		Ldlr$^{-/-}$		Mc4r^{mut}		Mc4r^{mut}; Ldlr$^{-/-}$			
Ensembl Gene ID	log2 fold change	p-value	log2 fold change	p-value	log2 fold change	p-value	log2 fold change	p-value	log2 fold change	p-value	log2 fold change	p-value	log2 fold change	p-value	name	description
ENSMUSG00000079057	-0.32	2.8E-02	-0.66	4.0E-04	-0.73	1.3E-03	-0.68	3.7E-04	-0.81	6.0E-05	-0.81	1.6E-03	-1.10	9.4E-05	Cyp4v3	cytochrome P450, family 4, subfamily v, polypeptide 3
ENSMUSG00000028240	-0.55	6.1E-01	-0.53	4.4E-01	-0.68	6.0E-01	-0.57	5.5E-01	-0.30	9.1E-01	-0.80	8.4E-01	-0.03	8.2E-01	Cyp7a1	cytochrome P450, family 7, subfamily a, polypeptide 1
ENSMUSG00000039519	-0.93	7.6E-03	-1.50	2.5E-04	-2.24	1.6E-06	-1.48	7.1E-05	-2.39	3.5E-12	-3.21	1.5E-18	-4.42	3.3E-25	Cyp7b1	cytochrome P450, family 7, subfamily b, polypeptide 1
ENSMUSG00000050445	-0.45	9.0E-03	0.41	3.0E-02	-0.09	9.4E-01	-0.78	2.3E-04	-0.62	1.3E-02	-0.53	1.1E-01	-0.50	6.8E-02	Cyp8b1	cytochrome P450, family 8, subfamily b, polypeptide 1
ENSMUSG00000030641	0.26	5.6E-01	1.12	3.0E-02	1.78	7.8E-05	1.21	2.5E-03	0.38	2.4E-01	1.40	9.9E-04	1.57	1.9E-03	Ddias	DNA damage-induced apoptosis suppressor
ENSMUSG00000028223	0.22	4.8E-01	0.43	1.7E-02	0.44	3.5E-02	0.43	1.6E-02	0.41	4.8E-02	0.72	7.2E-02	0.38	2.8E-01	Decr1	2,4-dienoyl CoA reductase 1, mitochondrial
ENSMUSG00000022419	-0.45	3.6E-03	-0.40	8.9E-03	-0.49	2.2E-02	-0.42	1.1E-02	-0.31	5.1E-02	-0.47	1.2E-01	-0.58	4.1E-02	Deptor	DEP domain containing MTOR-interacting protein
ENSMUSG00000029821	0.30	1.2E-01	0.38	4.7E-02	0.50	2.3E-02	0.53	2.3E-03	0.46	5.1E-02	0.72	1.8E-02	0.77	4.0E-02	Dfna5	deafness, autosomal dominant 5 (human)
ENSMUSG00000034480	-0.45	3.2E-02	-0.75	1.1E-03	-0.62	3.6E-02	-0.56	2.5E-02	-0.42	1.3E-01	-0.48	1.0E-01	-0.18	5.3E-01	Diaph2	diaphanous related formin 2
ENSMUSG00000038060	0.16	8.0E-01	1.01	2.3E-04	0.73	1.7E-03	0.86	6.4E-03	0.27	1.1E-01	1.13	6.2E-05	0.65	2.2E-02	Dlec1	deleted in lung and esophageal cancer 1
ENSMUSG00000030409	0.65	4.7E-03	0.57	2.8E-02	1.06	2.2E-03	0.54	3.1E-02	1.14	9.9E-04	1.13	3.3E-05	2.01	1.3E-08	Dmpk	dystrophia myotonica-protein kinase
ENSMUSG00000041268	-0.19	3.0E-01	-0.40	1.9E-02	-0.51	3.4E-02	-0.47	3.3E-02	-0.52	1.1E-02	-0.32	2.6E-01	-0.47	1.8E-01	Dmxl2	Dmx-like 2
ENSMUSG00000032285	-0.69	4.5E-03	-1.00	2.4E-04	-1.33	8.0E-05	-1.16	2.6E-04	-1.24	3.5E-05	-1.44	5.1E-06	-1.23	3.1E-04	Dnaja4	DnaJ heat shock protein family (Hsp40) member A4
ENSMUSG00000025014	1.87	3.1E-04	2.34	9.0E-08	2.51	3.3E-08	2.46	4.1E-11	2.93	7.8E-17	2.73	8.7E-08	2.92	8.1E-10	Dntt	deoxynucleotidyltransferase, terminal
ENSMUSG00000043671	-0.26	3.8E-01	-0.50	2.6E-02	-0.58	3.6E-02	-0.75	2.0E-03	-1.10	1.5E-05	-1.06	3.7E-04	-1.93	5.2E-09	Dpy19l3	dpy-19-like 3 (C. elegans)
ENSMUSG00000021928	0.33	1.1E-01	0.59	5.8E-03	0.64	6.7E-03	0.46	4.1E-02	0.60	2.1E-02	0.77	2.3E-01	0.69	2.4E-01	Ebpl	emopamil binding protein-like
ENSMUSG00000053898	0.25	3.3E-01	0.54	2.5E-03	0.50	2.7E-02	0.43	1.7E-02	0.34	5.6E-02	0.62	1.2E-01	0.23	4.8E-01	Ech1	enoyl coenzyme A hydratase 1, peroxisomal
ENSMUSG00000020122	-0.37	4.8E-01	-1.19	1.1E-03	-1.35	7.5E-04	-0.61	1.2E-01	-0.85	2.2E-02	-2.08	1.3E-07	-2.14	1.3E-07	Egfr	epidermal growth factor receptor
ENSMUSG00000085091	NA	NA	NA	NA	NA	NA	NA	NA	-0.83	2.4E-02	-2.14	3.8E-06	-1.74	1.9E-04	Egfros	epidermal growth factor receptor, opposite strand
ENSMUSG00000006390	0.08	2.8E-01	0.22	3.2E-01	0.05	5.0E-01	0.05	6.5E-01	0.00	5.7E-01	0.09	4.2E-01	0.22	2.1E-01	Elovl1	elongation of very long chain fatty acids (FEN1/Elo2, SUR4/Elo3, yeast)-like 1
ENSMUSG00000021364	0.20	4.4E-01	0.24	4.2E-01	0.42	9.9E-02	0.73	2.8E-03	0.88	6.1E-05	0.46	1.4E-01	0.36	4.1E-01	Elovl2	elongation of very long chain fatty acids (FEN1/Elo2, SUR4/Elo3, yeast)-like 2
ENSMUSG00000038754	-0.47	6.9E-02	-0.52	1.3E-01	-1.17	1.9E-02	-1.04	6.2E-03	-1.77	1.7E-08	-2.42	2.2E-12	-3.45	1.1E-21	Elovl3	elongation of very long chain fatty acids (FEN1/Elo2, SUR4/Elo3, yeast)-like 3
ENSMUSG00000032349	0.37	4.3E-01	1.36	6.1E-04	1.34	4.3E-04	1.21	9.1E-05	1.34	1.4E-05	1.70	1.1E-07	1.66	5.9E-07	Elovl5	ELOVL family member 5, elongation of long chain fatty acids (yeast)
ENSMUSG00000041220	0.12	5.0E-01	1.26	1.9E-01	1.39	1.2E-01	0.59	4.6E-01	1.60	9.0E-04	1.33	4.3E-03	1.80	3.7E-04	Elovl6	ELOVL family member 6, elongation of long chain fatty acids (yeast)
ENSMUSG00000021696	-1.49	1.1E-01	0.55	9.1E-01	0.04	4.4E-01	0.56	9.5E-01	0.47	7.1E-01	1.92	2.4E-01	2.29	7.6E-03	Elovl7	ELOVL family member 7, elongation of long chain fatty acids (yeast)
ENSMUSG00000041773	-0.32	2.8E-01	1.15	1.3E-03	1.09	1.2E-02	1.16	3.1E-03	0.72	5.7E-02	1.90	1.5E-07	2.01	5.1E-07	Enc1	ectodermal-neural cortex 1
ENSMUSG00000021236	-0.08	2.9E-01	0.53	3.9E-02	0.39	1.0E-01	-0.08	7.9E-01	-0.20	4.9E-01	0.59	3.2E-02	0.37	2.7E-01	Entpd5	ectonucleoside triphosphate diphosphohydrolase 5
ENSMUSG00000028434	-0.20	2.7E-01	-0.49	1.8E-02	-0.58	2.2E-02	-0.53	1.7E-02	-0.59	1.5E-02	-0.98	1.1E-03	-1.13	9.9E-04	Epb41l4b	erythrocyte membrane protein band 4.1 like 4b
ENSMUSG00000046324	-0.07	4.7E-01	0.70	4.9E-03	0.62	2.0E-02	0.23	3.4E-01	0.16	5.0E-01	0.90	2.5E-03	1.06	6.0E-04	Ermp1	endoplasmic reticulum metallopeptidase 1
ENSMUSG00000021996	0.16	7.8E-01	0.28	1.9E-01	0.31	2.6E-01	0.16	4.6E-01	0.30	3.8E-01	0.43	5.1E-01	0.29	7.8E-01	Esd	esterase D/formylglutathione hydrolase
ENSMUSG00000034584	-0.28	6.1E-02	-0.53	1.9E-02	-0.62	5.2E-03	-0.61	2.7E-03	-0.55	1.1E-02	-1.13	6.7E-04	-0.94	3.3E-03	Exph5	exophilin 5
ENSMUSG00000031645	-0.19	4.5E-01	-0.75	6.9E-04	-0.67	6.9E-03	-0.42	4.0E-02	-0.45	2.1E-02	-0.97	6.9E-04	-0.98	9.4E-04	F11	coagulation factor XI
ENSMUSG00000031443	0.07	6.1E-01	-0.26	2.7E-01	-0.22	2.7E-01	-0.34	5.2E-02	-0.18	5.7E-01	-0.75	2.1E-02	-0.85	8.6E-03	F7	coagulation factor VII
ENSMUSG00000054422	0.23	8.1E-01	0.56	2.2E-02	0.48	8.6E-02	0.80	2.2E-03	0.61	7.0E-02	1.05	3.2E-01	0.26	7.8E-01	Fabp1	fatty acid binding protein 1, liver
ENSMUSG00000027530	NA	NA	0.99	4.0E-01	NA	NA	1.03	9.4E-02	NA	NA	NA	NA	NA	NA	Fabp12	fatty acid binding protein 12

	regular chow						semisynthetic diet									
	Ldlr$^{-/-}$		Mc4r^{mut}		Mc4r^{mut}; Ldlr$^{-/-}$		wt		Ldlr$^{-/-}$		Mc4r^{mut}		Mc4r^{mut}; Ldlr$^{-/-}$			
**Ensembl Gene ID**	**log2 fold change**	**p-value**	**log2 fold change**	**p-value**	**log2 fold change**	**p-value**	**log2 fold change**	**p-value**	**log2 fold change**	**p-value**	**log2 fold change**	**p-value**	**log2 fold change**	**p-value**	**name**	**description**
ENSMUSG00000023057	0.68	1.4E-01	1.46	1.9E-07	1.44	6.4E-04	2.02	1.6E-12	2.08	1.1E-04	2.42	7.2E-02	2.00	1.5E-01	Fabp2	fatty acid binding protein 2, intestinal
ENSMUSG00000028773	NA	NA	NA	NA	0.26	9.4E-01	-0.36	8.6E-01	NA	NA	3.32	1.9E-01	0.26	8.8E-01	Fabp3	fatty acid binding protein 3, muscle and heart
ENSMUSG00000056366	NA	NA	NA	NA	NA	NA	-1.00	7.0E-01	-0.03	9.4E-01	0.33	8.4E-01	1.17	1.0E+00	Fabp3-ps1	fatty acid binding protein 3, muscle and heart, pseudogene 1
ENSMUSG00000062515	0.35	5.8E-02	0.81	8.2E-03	1.19	2.7E-02	0.95	2.4E-03	1.02	4.3E-03	2.59	2.1E-01	2.12	7.8E-02	Fabp4	fatty acid binding protein 4, adipocyte
ENSMUSG00000027533	0.07	6.4E-01	-0.14	4.9E-01	-0.52	9.6E-02	0.16	9.1E-01	0.56	2.3E-01	-0.51	8.1E-02	0.11	9.1E-01	Fabp5	fatty acid binding protein 5, epidermal
ENSMUSG00000019874	0.78	5.4E-03	0.07	4.6E-01	0.92	4.4E-04	0.30	1.0E-01	1.34	2.0E-02	1.24	4.0E-01	1.69	5.2E-02	Fabp7	fatty acid binding protein 7, brain
ENSMUSG00000010663	0.28	2.0E-01	0.61	5.8E-03	0.57	2.0E-02	0.78	1.1E-03	1.08	5.6E-07	0.98	4.4E-04	1.22	6.7E-05	Fads1	fatty acid desaturase 1
ENSMUSG00000024665	0.30	1.6E-01	0.83	1.7E-02	0.85	9.0E-04	1.35	5.0E-09	1.77	1.4E-16	1.66	2.0E-09	1.96	1.0E-09	Fads2	fatty acid desaturase 2
ENSMUSG00000024664	0.19	3.7E-01	0.08	9.6E-01	0.27	4.1E-01	0.62	3.1E-02	0.92	2.9E-04	1.38	9.4E-04	1.64	5.8E-06	Fads3	fatty acid desaturase 3
ENSMUSG00000044788	-0.22	1.8E-01	-0.11	4.4E-01	-0.19	3.7E-01	-0.32	4.0E-02	-0.18	4.0E-01	-0.29	5.1E-01	-0.30	3.6E-01	Fads6	fatty acid desaturase domain family, member 6
ENSMUSG00000039157	-0.01	8.2E-01	0.88	3.7E-03	0.56	2.4E-02	0.94	6.9E-03	0.88	2.4E-03	1.41	9.4E-07	1.15	4.6E-04	Fam102a	family with sequence similarity 102, member A
ENSMUSG00000049687	-5.79	2.1E-02	-5.55	3.2E-02	-4.90	4.2E-02	-6.00	1.3E-02	-5.23	3.0E-02	-4.60	3.4E-02	-4.91	4.4E-02	Fam109b	family with sequence similarity 109, member B
ENSMUSG00000030207	-0.32	1.3E-01	-0.69	2.6E-02	-0.67	6.1E-03	-0.69	4.4E-04	-0.62	1.2E-02	-1.01	5.0E-03	-0.80	2.8E-02	Fam234b	family with sequence similarity 234, member B
ENSMUSG00000051225	2.08	2.4E-03	2.96	5.5E-03	3.50	6.2E-08	3.09	4.0E-02	3.21	1.2E-02	4.53	1.9E-08	5.55	1.7E-08	Fam83a	family with sequence similarity 83, member A
ENSMUSG00000024778	0.15	2.2E-01	0.40	1.7E-02	0.41	5.5E-02	0.11	2.5E-01	0.17	6.9E-01	0.40	3.9E-01	0.25	3.2E-01	Fas	Fas (TNF receptor superfamily member 6)
ENSMUSG00000000817	0.46	1.0E+00	0.24	8.2E-01	-0.88	5.3E-01	-0.54	5.9E-01	1.55	1.1E-01	-0.01	1.0E+00	0.56	1.0E+00	Fasl	Fas ligand (TNF superfamily, member 6)
ENSMUSG00000025153	-0.05	4.2E-01	1.20	1.9E-01	0.80	2.6E-01	0.42	8.7E-01	1.55	4.4E-02	1.33	1.1E-01	1.84	1.3E-02	Fasn	fatty acid synthase
ENSMUSG00000032898	-0.48	1.3E-02	-0.59	4.3E-02	-0.85	1.0E-02	-0.68	4.6E-04	-0.87	2.0E-04	-0.65	7.1E-02	-0.93	2.3E-03	Fbxo21	F-box protein 21
ENSMUSG00000059498	0.60	1.3E-02	0.53	5.7E-03	0.89	1.9E-03	0.39	2.7E-02	1.21	1.3E-02	1.15	2.7E-02	1.88	2.7E-03	Fcgr3	Fc receptor, IgG, low affinity III
ENSMUSG00000037712	-0.34	2.0E-02	-0.42	1.9E-02	-0.75	2.8E-03	-0.75	3.1E-04	-0.69	4.5E-04	-0.88	5.0E-04	-1.07	6.4E-04	Fermt2	fermitin family member 2
ENSMUSG00000030827	-0.05	9.0E-01	1.91	5.5E-03	2.15	2.8E-05	2.49	1.8E-02	1.93	2.6E-09	2.92	5.8E-17	3.12	3.3E-15	Fgf21	fibroblast growth factor 21
ENSMUSG00000022215	0.02	7.1E-01	1.28	1.6E-06	0.83	8.6E-03	0.55	1.4E-01	0.17	7.1E-01	0.64	7.5E-02	0.22	7.7E-01	Fitm1	fat storage-inducing transmembrane protein 1
ENSMUSG00000048486	-0.10	2.5E-01	0.46	3.6E-02	0.23	6.4E-01	0.44	9.0E-02	0.16	7.3E-01	0.57	3.1E-02	0.39	2.8E-01	Fitm2	fat storage-inducing transmembrane protein 2
ENSMUSG00000033713	-0.50	4.8E-02	-0.43	2.7E-02	-0.58	4.7E-02	-0.59	7.5E-04	-0.68	1.3E-03	-0.80	6.9E-03	-0.74	2.4E-02	Foxn3	forkhead box N3
ENSMUSG00000030067	-0.23	2.4E-01	-0.62	2.9E-03	-0.67	2.4E-02	-0.51	3.7E-03	-0.75	3.4E-04	-0.69	9.7E-03	-0.60	7.1E-02	Foxp1	forkhead box P1
ENSMUSG00000033676	-1.00	1.4E-04	-0.84	1.5E-02	-1.59	3.3E-06	-1.05	7.1E-03	-1.72	5.3E-06	-0.77	1.1E-02	-0.95	1.9E-02	Gabrb3	gamma-aminobutyric acid (GABA) A receptor, subunit beta 3
ENSMUSG00000049721	0.20	9.5E-01	2.19	1.8E-09	2.28	7.4E-06	2.10	4.3E-03	1.81	1.1E-03	3.16	1.7E-15	2.90	1.7E-15	Gal3st1	galactose-3-O-sulfotransferase 1
ENSMUSG00000037280	1.22	9.9E-03	0.78	4.5E-02	1.11	3.3E-02	0.70	3.7E-02	1.16	7.6E-05	0.83	8.3E-03	2.27	1.4E-05	Galnt6	UDP-N-acetyl-alpha-D-galactosamine:polypeptide N-acetylgalactosaminyltransferase 6
ENSMUSG00000091387	-0.55	8.8E-02	-1.06	2.0E-03	-0.97	1.2E-02	-0.91	8.7E-03	-0.81	6.5E-03	-1.16	3.6E-03	-1.57	6.1E-03	Gcnt4	glucosaminyl (N-acetyl) transferase 4, core 2 (beta-1,6-N-acetylglucosaminyltransferase)
ENSMUSG00000027774	-0.01	7.9E-01	0.18	6.1E-01	0.05	9.0E-01	0.05	8.5E-01	0.04	6.8E-01	0.30	3.9E-01	0.12	7.7E-01	Gfm1	G elongation factor, mitochondrial 1
ENSMUSG00000029992	-0.24	1.3E-01	-0.37	2.1E-02	-0.52	4.3E-02	-0.61	4.0E-02	-0.62	3.1E-03	-0.67	4.3E-03	-0.41	1.1E-01	Gfpt1	glutamine fructose-6-phosphate transaminase 1
ENSMUSG00000048000	-0.38	3.4E-02	-0.57	4.6E-03	-0.52	1.8E-02	-0.51	6.4E-03	-0.56	2.5E-03	-0.54	3.0E-02	-0.49	8.4E-02	Gigyf2	GRB10 interacting GYF protein 2
ENSMUSG00000047797	0.05	8.7E-01	-0.12	5.5E-01	-0.28	3.1E-01	-0.28	1.6E-01	-0.43	7.4E-02	-0.80	7.0E-03	-0.86	8.2E-03	Gjb1	gap junction protein, beta 1
ENSMUSG00000071204	-0.50	9.3E-02	-1.03	1.0E-02	-0.56	3.5E-02	-0.97	8.7E-03	-1.11	6.7E-04	-1.06	4.9E-03	-1.08	2.1E-03	Gm10319	predicted pseudogene 10319
ENSMUSG00000074373	0.41	1.5E-01	2.49	2.2E-02	2.75	2.6E-04	3.76	2.6E-08	3.80	4.8E-18	5.06	1.5E-13	4.94	2.0E-25	Gm10680	predicted gene 10680

	regular chow						semisynthetic diet									
	Ldlr$^{-/-}$		Mc4r^{mut}		Mc4r^{mut}; Ldlr$^{-/-}$		wt		Ldlr$^{-/-}$		Mc4r^{mut}		Mc4r^{mut}; Ldlr$^{-/-}$			
**Ensembl Gene ID**	**log2 fold change**	**p-value**	**log2 fold change**	**p-value**	**log2 fold change**	**p-value**	**log2 fold change**	**p-value**	**log2 fold change**	**p-value**	**log2 fold change**	**p-value**	**log2 fold change**	**p-value**	**name**	**description**
ENSMUSG00000074380	-1.44	1.1E-02	-2.65	7.3E-04	-3.27	2.8E-04	-1.27	4.0E-02	-1.74	1.4E-03	-2.53	7.6E-03	-5.09	5.7E-08	Gm10681	predicted gene 10681
ENSMUSG00000085651	2.58	1.5E-03	2.70	4.6E-04	3.61	9.5E-06	2.55	9.2E-04	3.31	4.2E-04	3.79	1.2E-08	4.21	6.6E-10	Gm11695	predicted gene 11695
ENSMUSG00000086819	-0.19	5.3E-01	-0.48	2.1E-02	-0.60	4.5E-02	-0.59	2.3E-02	-0.63	8.4E-03	-0.76	1.5E-02	-1.02	2.8E-03	Gm13613	predicted gene 13613
ENSMUSG00000087185	-0.06	3.0E-01	-0.93	1.5E-03	-1.15	8.6E-05	-0.97	2.1E-04	-0.73	7.2E-03	-0.69	4.0E-02	-1.15	1.8E-03	Gm13872	predicted gene 13872
ENSMUSG00000085990	-0.56	2.6E-01	-1.29	1.3E-02	-1.13	4.7E-02	-1.33	7.7E-03	-0.97	2.7E-02	-1.57	6.0E-03	-1.15	5.1E-02	Gm16731	predicted gene, 16731
ENSMUSG00000085328	-0.26	1.4E-01	-0.69	3.1E-03	-0.37	4.5E-02	-0.47	4.4E-02	-0.45	4.1E-03	-0.99	1.6E-04	-0.65	9.3E-03	Gm17131	predicted gene 17131
ENSMUSG00000090290	-0.64	4.8E-02	-1.16	2.6E-03	-0.86	1.2E-02	-0.79	6.5E-03	-0.68	8.1E-03	-0.59	3.5E-02	-0.58	2.3E-01	Gm17296	predicted gene, 17296
ENSMUSG00000091038	-0.39	1.5E-01	-0.93	1.6E-04	-0.93	1.1E-02	-1.06	9.7E-05	-1.01	1.1E-04	-1.63	2.7E-07	-1.92	3.8E-07	Gm17662	predicted gene, 17662
ENSMUSG00000091489	-0.36	3.2E-01	-1.24	2.2E-03	-1.51	1.9E-03	-0.91	1.4E-02	-0.87	2.9E-02	-2.17	8.7E-06	-1.80	3.2E-05	Gm17664	predicted gene, 17664
ENSMUSG00000076036	0.33	4.1E-02	0.75	9.8E-04	0.76	5.3E-04	0.43	2.7E-02	0.39	3.0E-02	0.79	8.9E-03	0.93	5.4E-03	Gm22133	predicted gene, 22133
ENSMUSG00000090817	-1.36	2.1E-03	-2.37	1.6E-05	-3.41	2.1E-09	-1.68	9.3E-05	-1.43	3.3E-04	-5.23	4.5E-17	-4.27	2.0E-12	Gm4450	predicted gene 4450
ENSMUSG00000066538	-0.19	7.9E-02	-0.40	4.2E-02	-0.30	4.8E-02	-0.56	3.2E-03	-0.20	6.9E-02	-0.38	2.8E-02	-0.36	8.3E-02	Gm6254	predicted gene 6254
ENSMUSG00000060791	0.78	3.1E-04	0.85	4.4E-04	0.96	1.0E-04	0.46	9.0E-03	0.71	1.1E-02	0.88	2.3E-01	1.10	2.4E-01	Gmfg	glia maturation factor, gamma
ENSMUSG00000046338	0.09	6.9E-01	0.16	5.2E-01	0.36	4.5E-01	0.17	4.8E-01	0.11	3.5E-01	0.38	1.8E-01	0.26	6.4E-01	Gpat2	glycerol-3-phosphate acyltransferase 2, mitochondrial
ENSMUSG00000031545	0.26	5.1E-02	0.20	3.7E-02	0.22	1.3E-01	0.38	2.2E-02	0.26	9.5E-02	0.04	4.0E-01	-0.08	9.0E-01	Gpat4	glycerol-3-phosphate acyltransferase 4
ENSMUSG00000034220	0.14	4.9E-01	1.43	2.2E-03	1.19	3.8E-02	1.58	2.5E-03	1.48	1.3E-06	1.98	8.3E-07	1.28	1.4E-02	Gpc1	glypican 1
ENSMUSG00000008734	1.75	1.2E-01	5.69	4.9E-05	5.81	1.5E-16	3.80	2.6E-03	3.47	1.9E-03	4.95	3.7E-19	5.81	3.2E-21	Gprc5b	G protein-coupled receptor, family C, group 5, member B
ENSMUSG00000075706	0.32	7.2E-02	0.84	4.6E-05	0.66	2.7E-03	0.52	2.7E-03	0.54	1.2E-01	1.35	2.7E-01	1.20	1.7E-01	Gpx4	glutathione peroxidase 4
ENSMUSG00000024211	-0.62	1.4E-01	-1.68	3.3E-04	-1.76	3.6E-03	-0.87	2.7E-03	-1.12	3.8E-04	-2.93	1.6E-11	-4.70	9.5E-23	Grm8	glutamate receptor, metabotropic 8
ENSMUSG00000034708	0.42	1.5E-02	0.73	5.8E-06	0.94	2.6E-04	0.72	3.8E-03	0.81	1.4E-03	0.95	2.4E-05	1.34	3.1E-06	Grn	granulin
ENSMUSG00000024580	0.47	1.1E-01	0.54	2.4E-03	0.80	5.6E-03	0.63	1.7E-02	0.74	1.6E-02	1.23	4.1E-02	0.88	1.6E-02	Grpel2	GrpE-like 2, mitochondrial
ENSMUSG00000034345	0.10	4.5E-01	0.39	4.4E-02	0.43	3.1E-02	0.46	1.4E-02	0.30	2.2E-01	0.69	2.3E-01	0.41	3.5E-01	Gtf2h5	general transcription factor IIH, polypeptide 5
ENSMUSG00000020444	0.29	5.8E-02	0.45	4.2E-03	0.35	3.7E-02	0.40	1.8E-02	0.35	7.0E-02	0.63	2.2E-01	0.43	3.9E-01	Guk1	guanylate kinase 1
ENSMUSG00000056870	-2.43	2.5E-06	-2.47	7.6E-07	-3.56	2.6E-10	-1.54	2.1E-04	-1.88	6.4E-06	-2.10	2.9E-07	-2.07	1.3E-04	Gulp1	GULP, engulfment adaptor PTB domain containing 1
ENSMUSG00000027984	0.09	7.8E-01	0.49	1.7E-02	0.45	7.9E-02	0.30	2.1E-01	0.34	6.7E-02	0.67	3.1E-02	0.46	1.9E-01	Hadh	hydroxyacyl-Coenzyme A dehydrogenase
ENSMUSG00000003283	0.68	1.2E-02	0.65	3.7E-02	1.15	3.4E-02	0.58	4.1E-02	1.06	8.1E-03	0.85	1.5E-03	1.76	5.5E-07	Hck	hemopoietic cell kinase
ENSMUSG00000035247	-0.30	3.2E-02	-0.49	8.1E-03	-0.42	4.3E-02	-0.51	7.4E-03	-0.55	3.3E-03	-0.51	7.3E-02	-0.59	4.4E-02	Hectd1	HECT domain containing 1
ENSMUSG00000025232	0.46	4.4E-03	0.71	1.4E-04	0.75	3.5E-04	0.43	1.4E-02	0.73	1.3E-04	1.18	2.9E-05	1.39	5.1E-06	Hexa	hexosaminidase A
ENSMUSG00000043421	0.25	3.0E-01	0.64	4.1E-02	0.86	1.6E-03	1.35	6.5E-03	0.57	2.1E-02	2.27	3.9E-01	1.09	1.4E-03	Hilpda	hypoxia inducible lipid droplet associated
ENSMUSG00000000628	0.95	3.3E-01	2.43	4.7E-05	2.62	4.3E-04	2.34	2.4E-03	1.88	1.9E-02	3.55	1.4E-12	3.46	8.1E-10	Hk2	hexokinase 2
ENSMUSG00000028672	0.29	2.0E-01	0.59	2.8E-03	0.51	2.1E-02	0.42	3.1E-02	0.47	1.0E-02	0.50	1.1E-01	0.44	1.6E-01	Hmgcl	3-hydroxy-3-methylglutaryl-Coenzyme A lyase
ENSMUSG00000021670	-0.19	8.0E-01	0.14	8.2E-01	-0.18	9.6E-01	-0.97	1.6E-02	0.15	2.5E-01	-0.58	1.7E-01	0.19	5.3E-01	Hmgcr	3-hydroxy-3-methylglutaryl-Coenzyme A reductase
ENSMUSG00000027875	0.05	5.0E-01	0.40	6.4E-02	0.37	3.4E-01	0.21	3.9E-01	0.23	2.9E-01	0.34	2.7E-01	0.23	6.0E-01	Hmgcs2	3-hydroxy-3-methylglutaryl-Coenzyme A synthase 2
ENSMUSG00000005413	0.71	3.4E-03	0.75	9.6E-05	1.29	1.2E-02	0.60	1.8E-02	1.22	1.8E-04	1.30	4.1E-02	1.41	9.6E-06	Hmox1	heme oxygenase 1
ENSMUSG00000031844	-0.26	1.4E-01	-0.41	2.6E-02	-0.85	5.8E-04	-0.48	2.9E-02	-0.63	2.5E-03	-1.04	5.9E-05	-1.16	4.0E-04	Hsd17b2	hydroxysteroid (17-beta) dehydrogenase 2

	regular chow						semisynthetic diet									
	Ldlr$^{-/-}$		Mc4r^{mut}		Mc4r^{mut}; Ldlr$^{-/-}$		wt		Ldlr$^{-/-}$		Mc4r^{mut}		Mc4r^{mut}; Ldlr$^{-/-}$			
**Ensembl Gene ID**	**log2 fold change**	**p-value**	**log2 fold change**	**p-value**	**log2 fold change**	**p-value**	**log2 fold change**	**p-value**	**log2 fold change**	**p-value**	**log2 fold change**	**p-value**	**log2 fold change**	**p-value**	**name**	**description**
ENSMUSG00000062410	-0.17	1.3E-01	-0.59	1.1E-04	-0.77	4.3E-03	-0.35	3.9E-02	-0.29	8.3E-02	-0.74	3.2E-03	-1.16	2.5E-05	Hsd3b3	hydroxy-delta-5-steroid dehydrogenase, 3 beta- and steroid delta-isomerase 3
ENSMUSG00000056351	-1.27	4.3E-03	-2.49	2.0E-05	-3.28	7.3E-07	-1.48	1.6E-03	-1.62	1.8E-04	-3.29	2.4E-06	-4.86	2.2E-12	Hsd3b4	hydroxy-delta-5-steroid dehydrogenase, 3 beta- and steroid delta-isomerase 4
ENSMUSG00000038092	-0.64	1.9E-02	-2.16	3.7E-18	-3.43	2.9E-29	-0.84	4.3E-02	-1.47	9.0E-08	-4.71	1.2E-48	-6.48	1.6E-60	Hsd3b5	hydroxy-delta-5-steroid dehydrogenase, 3 beta- and steroid delta-isomerase 5
ENSMUSG00000030541	0.30	2.4E-01	0.53	1.9E-02	0.45	6.3E-02	0.27	2.4E-01	0.30	3.0E-02	0.78	6.2E-03	0.77	6.7E-03	Idh2	isocitrate dehydrogenase 2 (NADP+), mitochondrial
ENSMUSG00000021208	0.47	3.2E-02	1.24	2.3E-04	1.57	1.3E-04	1.61	1.0E-02	1.30	1.4E-02	2.76	5.6E-08	2.66	6.6E-14	Ifi27l2b	interferon, alpha-inducible protein 27 like 2B
ENSMUSG00000031838	0.47	1.1E-02	0.44	2.3E-02	0.92	6.9E-03	0.55	1.1E-02	0.97	5.1E-05	0.75	4.6E-03	1.49	3.0E-06	Ifi30	interferon gamma inducible protein 30
ENSMUSG00000022967	-0.13	4.2E-01	-0.26	2.2E-01	-0.18	5.7E-01	-0.05	9.4E-01	-0.11	5.3E-01	-0.11	9.5E-01	-0.05	9.8E-01	Ifnar1	interferon (alpha and beta) receptor 1
ENSMUSG00000022971	0.24	7.2E-02	0.19	8.9E-02	0.31	1.3E-01	0.07	5.1E-01	0.02	5.9E-01	0.11	5.2E-01	0.06	6.7E-01	Ifnar2	interferon (alpha and beta) receptor 2
ENSMUSG00000022969	0.67	2.6E-04	0.50	3.1E-02	0.87	1.4E-04	0.53	7.7E-03	0.99	9.6E-06	1.05	2.7E-04	1.68	1.4E-06	Il10rb	interleukin 10 receptor, beta
ENSMUSG00000000791	-0.21	4.9E-01	-0.91	1.4E-02	-0.89	1.0E-03	-1.47	4.7E-05	-1.48	2.1E-05	-1.91	7.3E-05	-2.34	1.4E-04	Il12rb1	interleukin 12 receptor, beta 1
ENSMUSG00000026981	2.09	9.6E-02	1.34	2.1E-02	2.89	2.9E-04	2.02	6.5E-03	2.68	5.7E-09	3.39	5.3E-07	3.66	1.2E-10	Il1rn	interleukin 1 receptor antagonist
ENSMUSG00000027947	-0.13	5.3E-01	-0.55	7.7E-02	-0.55	3.6E-02	-0.52	7.4E-02	-0.27	2.1E-01	-1.30	1.3E-03	-1.00	8.4E-03	Il6ra	interleukin 6 receptor, alpha
ENSMUSG00000021756	-0.15	3.1E-01	-0.16	3.3E-01	-0.15	5.7E-01	-0.23	2.4E-01	-0.02	6.8E-01	0.01	9.0E-01	0.08	9.1E-01	Il6st	interleukin 6 signal transducer
ENSMUSG00000024525	0.41	4.1E-01	1.68	1.3E-04	1.37	1.8E-03	1.36	3.4E-03	1.20	5.3E-04	1.93	2.2E-06	2.13	3.6E-06	Impa2	inositol (myo)-1(or 4)-monophosphatase 2
ENSMUSG00000026638	-0.17	4.0E-01	-0.67	9.2E-04	-0.87	1.6E-03	-0.79	6.3E-04	-1.09	1.9E-07	-1.50	4.7E-07	-1.80	1.0E-06	Irf6	interferon regulatory factor 6
ENSMUSG00000055980	-0.34	7.5E-02	-0.61	4.8E-03	-0.64	1.8E-02	-0.60	9.4E-03	-0.84	2.8E-04	-0.68	5.0E-03	-0.77	2.0E-02	Irs1	insulin receptor substrate 1
ENSMUSG00000025780	-0.54	1.1E-02	-1.33	2.7E-06	-1.17	8.0E-05	-1.23	6.5E-06	-1.25	4.0E-06	-1.26	2.4E-05	-0.89	1.4E-02	Itih5	inter-alpha (globulin) inhibitor H5
ENSMUSG00000052684	0.00	7.6E-01	0.23	2.7E-01	0.05	8.4E-01	0.65	4.4E-02	0.31	2.9E-01	0.82	6.1E-03	0.55	2.2E-02	Jun	jun proto-oncogene
ENSMUSG00000061751	-0.06	3.2E-01	-0.46	4.2E-03	-0.43	9.3E-03	-0.34	4.3E-02	-0.19	1.7E-01	-0.47	5.1E-02	-0.59	3.0E-02	Kalrn	kalirin, RhoGEF kinase
ENSMUSG00000016940	0.05	5.2E-01	0.91	1.1E-03	0.65	2.4E-03	0.68	4.5E-03	0.62	4.2E-03	1.05	1.4E-04	0.96	1.6E-03	Kctd2	potassium channel tetramerisation domain containing 2
ENSMUSG00000009905	-0.10	6.0E-01	0.59	4.5E-03	0.64	9.7E-03	0.43	4.3E-02	0.36	8.6E-02	1.00	1.3E-03	1.00	2.4E-03	Kdsr	3-ketodihydrosphingosine reductase
ENSMUSG00000024795	0.67	3.6E-01	1.15	3.6E-02	1.61	7.1E-03	1.31	1.1E-02	1.13	3.1E-01	2.04	1.7E-02	2.28	3.3E-04	Kif20b	kinesin family member 20B
ENSMUSG00000030187	0.64	7.8E-03	0.51	4.7E-02	0.81	3.5E-02	0.60	3.1E-02	1.06	1.5E-02	1.13	4.3E-02	1.27	1.1E-03	Klra2	killer cell lectin-like receptor, subfamily A, member 2
ENSMUSG00000024421	-0.60	5.6E-03	-0.87	2.1E-03	-1.29	5.4E-03	-0.70	1.2E-02	-1.20	6.0E-06	-2.18	2.1E-10	-3.32	6.6E-15	Lama3	laminin, alpha 3
ENSMUSG00000026639	-0.06	7.4E-01	1.40	1.1E-04	1.78	3.0E-02	0.68	4.9E-02	0.46	6.3E-02	0.91	5.1E-03	0.94	3.4E-03	Lamb3	laminin, beta 3
ENSMUSG00000028581	0.82	6.0E-03	0.93	3.4E-04	1.31	1.2E-02	0.66	1.1E-02	1.39	1.1E-05	1.17	4.5E-06	2.42	3.9E-09	Laptm5	lysosomal-associated protein transmembrane 5
ENSMUSG00000068220	0.38	5.8E-01	1.89	4.2E-07	1.90	1.2E-04	1.37	5.0E-04	1.45	1.5E-02	2.89	1.2E-01	2.68	1.6E-03	Lgals1	lectin, galactose binding, soluble 1
ENSMUSG00000050335	1.06	1.6E-05	0.55	1.9E-03	1.93	3.7E-03	1.31	1.8E-04	1.74	1.6E-06	2.43	1.0E-03	3.27	2.5E-09	Lgals3	lectin, galactose binding, soluble 3
ENSMUSG00000054263	-0.44	6.8E-02	-1.36	1.4E-07	-1.52	9.4E-07	-1.05	3.1E-04	-1.24	2.0E-07	-2.27	2.2E-13	-2.53	7.4E-14	Lifr	leukemia inhibitory factor receptor
ENSMUSG00000056394	0.20	2.5E-01	0.73	3.2E-02	0.97	5.8E-03	0.75	2.6E-02	0.72	9.3E-03	1.32	6.3E-05	1.39	6.9E-04	Lig1	ligase I, DNA, ATP-dependent
ENSMUSG00000020697	-0.28	2.1E-01	-0.53	7.2E-03	-0.66	1.2E-02	-0.53	1.6E-02	-0.76	2.1E-03	-0.74	2.5E-02	-0.67	9.8E-02	Lig3	ligase III, DNA, ATP-dependent
ENSMUSG00000029674	0.43	9.3E-02	1.45	3.7E-03	1.50	1.7E-02	0.81	2.3E-02	0.52	4.5E-02	1.26	1.1E-04	1.55	1.7E-05	Limk1	LIM-domain containing, protein kinase
ENSMUSG00000024766	0.80	3.6E-03	0.46	3.9E-02	1.12	6.6E-04	0.60	3.9E-03	NA	NA	0.93	2.6E-03	NA	NA	Lipo3	lipase, member O3
ENSMUSG00000022500	0.55	6.4E-02	0.72	2.5E-03	0.59	3.9E-02	0.55	2.1E-03	0.47	7.9E-02	0.51	2.6E-02	0.65	5.6E-03	Litaf	LPS-induced TN factor
ENSMUSG00000041891	-0.17	2.5E-01	-0.59	5.3E-04	-0.53	4.0E-02	-0.45	2.3E-02	-0.25	3.3E-01	-0.70	1.5E-02	-0.59	3.6E-02	Lman1	lectin, mannose-binding, 1

	regular chow						semisynthetic diet									
	Ldlr$^{-/-}$		Mc4r^{mut}		Mc4r^{mut}; Ldlr$^{-/-}$		wt		Ldlr$^{-/-}$		Mc4r^{mut}		Mc4r^{mut}; Ldlr$^{-/-}$			
Ensembl Gene ID	log2 fold change	p-value	log2 fold change	p-value	log2 fold change	p-value	log2 fold change	p-value	log2 fold change	p-value	log2 fold change	p-value	log2 fold change	p-value	name	description
ENSMUSG00000030029	-0.32	2.0E-02	-0.38	2.9E-02	-0.57	3.2E-03	-0.39	2.1E-02	-0.49	4.3E-03	-0.62	1.3E-02	-0.80	1.7E-02	Lrig1	leucine-rich repeats and immunoglobulin-like domains 1
ENSMUSG00000049988	0.93	3.4E-03	0.73	3.8E-02	1.19	3.9E-03	0.68	4.9E-02	1.18	9.1E-04	0.90	5.5E-03	1.73	4.4E-05	Lrrc25	leucine rich repeat containing 25
ENSMUSG00000027961	0.13	9.3E-01	1.22	4.4E-03	1.31	2.9E-03	1.31	1.3E-03	1.04	1.5E-02	2.19	6.5E-04	2.12	8.9E-03	Lrrc39	leucine rich repeat containing 39
ENSMUSG00000022584	0.84	5.9E-02	0.82	1.8E-02	1.14	3.7E-02	0.94	4.9E-03	1.36	1.1E-02	1.01	2.2E-03	1.08	9.9E-03	Ly6c2	lymphocyte antigen 6 complex, locus C2
ENSMUSG00000034634	1.64	5.3E-03	4.91	1.2E-04	5.96	2.7E-03	5.54	7.5E-03	4.80	2.0E-02	7.58	1.8E-03	7.73	2.7E-12	Ly6d	lymphocyte antigen 6 complex, locus D
ENSMUSG00000061143	-0.13	9.6E-01	-1.39	5.2E-05	-1.23	6.0E-04	-1.07	1.5E-03	-1.02	8.6E-03	-1.06	1.1E-02	-0.92	3.2E-02	Maml3	mastermind like 3 (Drosophila)
ENSMUSG00000037236	0.07	8.3E-01	-0.35	9.2E-02	-0.12	7.0E-01	-0.03	9.6E-01	0.03	9.4E-01	-0.22	2.9E-01	-0.20	3.6E-01	Matr3	matrin 3
ENSMUSG00000024863	0.67	3.2E-04	0.70	1.5E-04	0.80	2.2E-04	0.53	5.6E-03	0.57	3.6E-03	0.72	5.8E-02	0.17	6.8E-01	Mbl2	mannose-binding lectin (protein C) 2
ENSMUSG00000071856	-0.30	1.6E-01	-0.67	2.7E-02	-1.18	1.6E-04	-0.69	5.1E-03	-0.47	6.3E-02	-1.05	3.4E-03	-1.12	9.6E-05	Mcc	mutated in colorectal cancers
ENSMUSG00000032418	0.40	8.7E-01	1.54	3.7E-03	1.29	1.2E-02	0.74	1.6E-01	1.09	1.9E-03	1.54	2.7E-03	1.36	7.7E-03	Me1	malic enzyme 1, NADP(+)-dependent, cytosolic
ENSMUSG00000039208	1.02	2.4E-02	0.77	4.0E-02	1.26	8.4E-04	0.84	1.0E-02	1.24	2.8E-05	1.53	1.8E-01	1.69	1.0E-04	Metrnl	meteorin, glial cell differentiation regulator-like
ENSMUSG00000028655	1.10	1.4E-02	1.96	6.5E-04	2.20	2.5E-08	2.27	6.6E-10	2.44	9.3E-14	2.58	1.9E-11	2.34	3.1E-09	Mfsd2a	major facilitator superfamily domain containing 2A
ENSMUSG00000022978	0.10	3.9E-01	0.46	3.6E-02	0.64	5.0E-03	0.41	3.9E-02	0.69	2.1E-03	0.92	3.4E-03	0.87	6.4E-03	Mis18a	MIS18 kinetochore protein A
ENSMUSG00000068011	0.26	2.0E-01	0.66	7.6E-03	0.63	4.2E-03	0.47	1.5E-02	0.53	1.8E-02	1.08	7.0E-02	1.09	8.9E-02	Mkrn2os	makorin, ring finger protein 2, opposite strand
ENSMUSG00000025159	0.02	6.0E-01	-0.02	5.9E-01	0.02	8.5E-01	0.22	3.0E-01	-0.02	9.3E-01	0.15	3.0E-01	-0.05	8.9E-01	Mms19	MMS19 (MET18 S. cerevisiae)
ENSMUSG00000012187	0.29	7.3E-01	2.56	2.2E-04	2.50	1.9E-03	1.94	1.8E-03	1.60	2.6E-02	3.44	6.0E-07	2.98	1.3E-12	Mogat1	monoacylglycerol O-acyltransferase 1
ENSMUSG00000052396	1.53	1.7E-01	1.85	1.7E-01	2.08	8.7E-02	-0.67	5.3E-01	1.30	3.3E-01	1.97	6.4E-02	2.18	1.1E-01	Mogat2	monoacylglycerol O-acyltransferase 2
ENSMUSG00000034543	-0.30	2.6E-01	-0.31	2.1E-01	-0.18	3.2E-01	0.09	7.8E-01	-0.11	3.7E-01	-0.09	7.9E-01	0.01	8.8E-01	Morc2a	microrchidia 2A
ENSMUSG00000039456	0.05	8.4E-01	0.11	8.4E-01	0.25	4.4E-01	0.05	8.1E-01	0.33	3.6E-01	0.28	5.0E-01	0.33	4.9E-01	Morc3	microrchidia 3
ENSMUSG00000031434	0.52	9.6E-02	1.49	3.1E-02	1.63	7.1E-04	1.26	1.3E-03	1.80	2.6E-05	2.76	1.5E-06	2.90	3.4E-13	Morc4	microrchidia 4
ENSMUSG00000020000	-2.26	5.3E-02	-4.57	2.4E-04	-4.57	9.7E-05	-2.19	2.0E-02	-2.38	3.7E-03	-8.79	5.6E-12	-9.10	2.8E-11	Moxd1	monooxygenase, DBH-like 1
ENSMUSG00000024683	-0.08	1.0E+00	0.27	2.4E-01	0.05	5.6E-01	0.10	3.7E-01	0.02	7.2E-01	0.19	5.8E-01	0.19	5.0E-01	Mrpl16	mitochondrial ribosomal protein L16
ENSMUSG00000024679	1.10	2.2E-02	0.97	1.1E-02	1.63	7.0E-03	1.03	1.6E-03	1.73	4.8E-06	1.88	4.5E-03	2.47	2.4E-04	Ms4a6d	membrane-spanning 4-domains, subfamily A, member 6D
ENSMUSG00000024672	0.18	2.9E-01	1.14	2.3E-02	1.40	5.9E-03	1.92	3.9E-02	1.76	1.3E-02	2.70	3.0E-03	3.35	1.9E-03	Ms4a7	membrane-spanning 4-domains, subfamily A, member 7
ENSMUSG00000054764	0.11	1.0E+00	1.16	5.7E-04	0.85	1.9E-02	2.16	3.2E-03	0.79	1.9E-02	1.57	2.5E-04	1.75	1.4E-03	Mtnr1a	melatonin receptor 1A
ENSMUSG00000059908	-0.80	2.7E-04	-1.20	6.6E-05	-1.25	6.6E-05	-1.05	1.2E-05	-1.29	1.4E-06	-1.43	2.4E-07	-1.81	6.4E-09	Mug1	murinoglobulin 1
ENSMUSG00000078680	-0.64	1.3E-02	-0.75	3.5E-02	-1.26	1.9E-03	-0.86	1.7E-02	-1.22	4.3E-05	-2.04	7.6E-09	-3.59	2.4E-17	Mup10	major urinary protein 10
ENSMUSG00000073834	-0.68	3.9E-02	-0.96	3.2E-02	-1.51	2.1E-03	-1.13	1.4E-02	-1.59	1.8E-04	-2.44	3.1E-06	-4.08	2.1E-10	Mup11	major urinary protein 11
ENSMUSG00000073839	-0.86	2.1E-02	-1.14	2.9E-02	-1.62	2.9E-03	-1.30	4.9E-03	-1.82	1.1E-04	-2.97	8.6E-07	-4.60	1.7E-10	Mup12	major urinary protein 12
ENSMUSG00000042451	0.43	2.2E-01	1.10	1.9E-03	1.17	1.1E-03	0.98	2.6E-02	0.93	9.4E-03	1.43	2.4E-04	0.96	3.8E-02	Mybph	myosin binding protein H
ENSMUSG00000048612	0.28	2.8E-01	0.59	2.5E-02	1.07	2.9E-02	0.66	3.5E-02	1.20	1.7E-03	2.00	1.2E-04	2.67	2.8E-08	Myof	myoferlin
ENSMUSG00000029063	0.03	6.8E-01	0.38	4.4E-02	0.30	9.4E-02	0.25	1.9E-01	0.34	4.8E-02	0.38	1.2E-01	0.35	1.5E-01	Nadk	NAD kinase
ENSMUSG00000022253	-0.08	4.9E-01	-0.13	4.3E-01	-0.11	8.5E-01	-0.41	4.8E-02	-0.20	2.9E-01	-0.51	4.2E-02	-0.48	7.8E-02	Nadk2	NAD kinase 2, mitochondrial
ENSMUSG00000034744	0.09	5.0E-01	0.40	7.1E-03	0.51	9.5E-03	0.42	1.8E-02	0.62	7.8E-04	1.02	3.2E-03	1.02	5.8E-04	Nagk	N-acetylglucosamine kinase
ENSMUSG00000020572	0.25	1.7E-01	0.25	3.0E-01	0.40	4.4E-02	0.43	2.6E-02	0.71	5.3E-03	0.77	2.5E-03	0.78	2.6E-02	Nampt	nicotinamide phosphoribosyltransferase

	regular chow						semisynthetic diet									
	$Ldlr^{-/-}$		$Mc4r^{mut}$		$Mc4r^{mut}$; $Ldlr^{-/-}$		wt		$Ldlr^{-/-}$		$Mc4r^{mut}$		$Mc4r^{mut}$; $Ldlr^{-/-}$			
**Ensembl Gene ID**	**log2 fold change**	**p-value**	**log2 fold change**	**p-value**	**log2 fold change**	**p-value**	**log2 fold change**	**p-value**	**log2 fold change**	**p-value**	**log2 fold change**	**p-value**	**log2 fold change**	**p-value**	**name**	**description**
ENSMUSG00000057103	-0.08	7.5E-01	-0.49	2.1E-02	-0.44	1.0E-01	-0.33	1.3E-01	-0.50	2.2E-02	-1.04	5.3E-04	-1.47	2.9E-06	Nat8f1	N-acetyltransferase 8 (GCN5-related) family member 1
ENSMUSG00000001911	-0.60	1.2E-02	-0.51	1.3E-02	-0.65	4.5E-02	-0.43	4.8E-02	-0.58	2.2E-02	-0.75	4.2E-02	-0.69	5.0E-02	Nfix	nuclear factor I/X
ENSMUSG00000072889	-0.28	2.6E-01	-0.65	3.4E-03	-0.63	3.2E-02	-0.75	8.7E-03	-0.89	1.1E-04	-0.97	3.7E-03	-0.90	8.8E-03	Nfxl1	nuclear transcription factor, X-box binding-like 1
ENSMUSG00000063480	-0.44	7.3E-02	-0.73	2.5E-02	-0.70	1.2E-02	-0.57	3.1E-02	-0.62	3.8E-02	-0.68	1.4E-02	-0.73	1.6E-02	Nhp2l1	NHP2 non-histone chromosome protein 2-like 1 (S. cerevisiae)
ENSMUSG00000039835	-0.58	2.6E-02	-0.69	1.2E-03	-0.95	9.1E-04	-0.82	3.9E-03	-1.16	6.9E-06	-1.51	8.0E-06	-1.06	7.6E-03	Nhsl1	NHS-like 1
ENSMUSG00000067219	2.53	6.4E-03	1.61	2.4E-02	3.02	1.1E-04	2.62	2.6E-02	3.13	9.3E-08	2.13	1.3E-02	3.43	2.8E-16	Nipal1	NIPA-like domain containing 1
ENSMUSG00000028469	-0.67	2.8E-04	-0.62	5.9E-03	-1.10	8.1E-07	-0.59	3.9E-03	-0.99	1.6E-06	-1.08	1.2E-03	-1.42	1.4E-05	Npr2	natriuretic peptide receptor 2
ENSMUSG00000060601	0.03	7.0E-01	0.13	4.5E-01	0.00	8.9E-01	0.08	6.5E-01	-0.16	8.2E-01	-0.03	7.7E-01	-0.11	9.2E-01	Nr1h2	nuclear receptor subfamily 1, group H, member 2
ENSMUSG00000064959	-6.34	1.6E-02	-6.13	2.7E-02	-5.28	3.9E-02	-6.74	1.0E-02	-5.66	2.5E-02	-4.72	3.4E-02	-6.20	2.8E-02	n-R5s41	nuclear encoded rRNA 5S 41
ENSMUSG00000055254	0.32	6.8E-01	1.90	4.4E-02	2.13	2.1E-02	1.92	9.8E-03	1.76	4.0E-02	2.77	3.0E-08	3.08	5.6E-06	Ntrk2	neurotrophic tyrosine kinase, receptor, type 2
ENSMUSG00000045211	-0.22	8.8E-01	0.60	9.6E-03	0.51	1.5E-02	0.78	1.6E-02	0.30	6.2E-02	1.14	1.2E-04	1.14	8.1E-05	Nudt18	nudix (nucleoside diphosphate linked moiety X)-type motif 18
ENSMUSG00000029310	0.01	5.4E-01	0.32	5.8E-02	0.29	1.2E-01	0.30	8.4E-02	0.17	1.9E-01	0.57	5.0E-02	0.42	1.1E-01	Nudt9	nudix (nucleoside diphosphate linked moiety X)-type motif 9
ENSMUSG00000022142	-0.23	1.8E-01	-0.28	2.7E-02	-0.48	2.8E-02	-0.37	5.0E-02	-0.53	1.1E-02	-0.32	1.6E-01	-0.53	9.9E-02	Nup155	nucleoporin 155
ENSMUSG00000030717	1.12	9.8E-02	1.40	2.4E-02	1.85	3.7E-04	1.55	1.1E-02	2.03	2.8E-04	2.86	1.1E-02	3.40	8.3E-06	Nupr1	nuclear protein transcription regulator 1
ENSMUSG00000029822	0.62	4.9E-01	2.76	3.7E-04	2.67	9.7E-05	2.66	6.5E-05	2.36	2.0E-04	4.00	1.0E-14	4.10	5.2E-25	Osbpl3	oxysterol binding protein-like 3
ENSMUSG00000029247	-0.04	6.9E-01	-0.46	1.4E-02	-0.53	1.9E-02	-0.59	1.6E-03	-0.46	9.3E-03	-0.83	9.1E-04	-1.06	4.5E-04	Paics	phosphoribosylaminoimidazole carboxylase, phosphoribosylaminoribosylaminoimidazole, [illegible]
ENSMUSG00000042167	0.25	2.8E-01	0.05	8.1E-01	0.13	4.5E-01	0.07	6.4E-01	-0.02	8.5E-01	0.11	8.9E-01	0.00	8.8E-01	Papd4	PAP associated domain containing 4
ENSMUSG00000036779	-0.13	3.5E-01	-0.24	2.2E-01	-0.13	7.0E-01	-0.16	4.6E-01	-0.20	2.9E-01	-0.19	5.2E-01	-0.30	3.0E-01	Papd5	PAP associated domain containing 5
ENSMUSG00000034575	-0.19	2.9E-01	-0.17	5.4E-01	-0.32	3.2E-01	-0.18	2.6E-01	-0.12	5.8E-01	-0.29	5.4E-01	-0.42	3.4E-01	Papd7	PAP associated domain containing 7
ENSMUSG00000021223	-0.46	1.3E-01	0.08	5.6E-01	-0.09	3.0E-01	-0.35	8.0E-02	0.12	6.8E-01	-0.59	1.6E-01	-0.31	7.1E-01	Papln	papilin, proteoglycan-like sulfated glycoprotein
ENSMUSG00000021111	0.01	8.7E-01	-0.12	4.5E-01	-0.13	8.5E-01	-0.10	8.7E-01	-0.11	5.3E-01	-0.13	4.5E-01	-0.24	4.4E-01	Papola	poly (A) polymerase alpha
ENSMUSG00000020273	-0.23	2.4E-01	-0.25	1.8E-01	-0.12	4.1E-01	-0.03	8.7E-01	0.00	6.7E-01	-0.06	3.6E-01	-0.15	6.4E-01	Papolg	poly(A) polymerase gamma
ENSMUSG00000028370	1.24	5.6E-01	NA	NA	0.72	7.8E-01	0.23	8.8E-01	0.88	1.0E+00	0.32	8.7E-01	1.98	4.6E-01	Pappa	pregnancy-associated plasma protein A
ENSMUSG00000028032	0.17	3.4E-01	-0.17	6.0E-01	-0.21	7.1E-01	-0.53	4.4E-02	-0.32	3.9E-01	-0.46	1.8E-01	-0.29	7.0E-01	Papss1	3'-phosphoadenosine 5'-phosphosulfate synthase 1
ENSMUSG00000024899	-0.12	3.2E-01	-0.17	2.4E-01	-0.08	5.3E-01	-0.26	6.3E-02	-0.01	8.8E-01	-0.38	2.9E-01	-0.32	2.5E-01	Papss2	3'-phosphoadenosine 5'-phosphosulfate synthase 2
ENSMUSG00000034422	-0.19	4.3E-01	-0.39	3.2E-02	-0.06	6.3E-01	-0.09	6.6E-01	0.21	4.5E-01	0.15	4.8E-01	0.42	2.6E-01	Parp14	poly (ADP-ribose) polymerase family, member 14
ENSMUSG00000032392	-0.49	4.8E-02	-0.66	3.7E-03	-0.61	2.5E-02	-0.49	2.0E-02	-0.67	4.2E-03	-0.74	2.4E-02	-0.72	6.8E-02	Parp16	poly (ADP-ribose) polymerase family, member 16
ENSMUSG00000014030	NA	NA	NA	NA	2.58	3.2E-01	1.18	9.7E-01	NA	NA	NA	NA	1.69	4.9E-01	Pax5	paired box 5
ENSMUSG00000020553	0.41	3.2E-01	0.94	6.2E-05	0.99	2.7E-04	0.76	1.8E-03	0.90	2.1E-04	0.92	4.6E-03	1.09	3.8E-03	Pctp	phosphatidylcholine transfer protein
ENSMUSG00000030545	0.01	5.2E-01	0.80	8.2E-04	0.64	2.4E-02	0.49	2.9E-02	0.39	1.8E-01	0.89	1.7E-03	0.81	1.6E-02	Pex11a	peroxisomal biogenesis factor 11 alpha
ENSMUSG00000026773	-0.87	1.0E-01	-1.77	1.4E-03	-2.04	1.6E-03	-1.04	4.3E-02	-1.10	2.1E-02	-1.08	3.0E-02	-1.61	9.3E-03	Pfkfb3	6-phosphofructo-2-kinase/fructose-2,6-biphosphatase 3
ENSMUSG00000022940	0.29	1.7E-01	0.55	2.6E-03	0.80	1.1E-04	0.57	9.3E-04	0.95	8.4E-02	1.20	3.6E-02	1.30	3.5E-02	Pigp	phosphatidylinositol glycan anchor biosynthesis, class P
ENSMUSG00000025229	-0.65	7.4E-02	-0.95	3.6E-02	-1.63	2.3E-02	-1.88	4.0E-04	-2.62	2.8E-06	-2.68	1.0E-05	-3.55	1.3E-09	Pitx3	paired-like homeodomain transcription factor 3
ENSMUSG00000023908	0.58	2.6E-01	1.26	4.1E-02	1.41	1.7E-02	1.05	4.9E-02	0.65	2.3E-01	1.30	1.5E-02	1.95	3.9E-04	Pkmyt1	protein kinase, membrane associated tyrosine/threonine 1
ENSMUSG00000040268	0.24	4.5E-01	0.87	1.2E-02	0.92	1.0E-02	0.61	3.1E-02	0.50	7.8E-02	1.30	3.5E-04	1.40	3.3E-05	Plekha1	pleckstrin homology domain containing, family A (phosphoinositide binding specific) member 1

	regular chow						semisynthetic diet									
	$Ldlr^{-/-}$		$Mc4r^{mut}$		$Mc4r^{mut}$; $Ldlr^{-/-}$		wt		$Ldlr^{-/-}$		$Mc4r^{mut}$		$Mc4r^{mut}$; $Ldlr^{-/-}$			
**Ensembl Gene ID**	**log2 fold change**	**p-value**	**log2 fold change**	**p-value**	**log2 fold change**	**p-value**	**log2 fold change**	**p-value**	**log2 fold change**	**p-value**	**log2 fold change**	**p-value**	**log2 fold change**	**p-value**	**name**	**description**
ENSMUSG00000028494	0.32	3.6E-01	0.93	1.3E-06	0.84	1.8E-03	0.88	2.2E-03	0.52	1.2E-02	1.30	2.7E-05	1.07	8.6E-04	Plin2	perilipin 2
ENSMUSG00000024197	0.01	1.0E+00	0.40	4.7E-02	0.29	2.6E-01	0.23	4.8E-01	0.45	1.5E-02	0.83	1.6E-03	0.97	1.6E-03	Plin3	perilipin 3
ENSMUSG00000002831	0.13	8.8E-01	1.78	4.6E-03	1.63	1.6E-05	1.16	1.6E-01	0.67	8.6E-01	2.22	7.2E-07	1.89	2.0E-05	Plin4	perilipin 4
ENSMUSG00000011305	0.29	1.9E-01	0.79	3.4E-05	0.64	1.9E-02	0.55	5.1E-02	0.25	1.8E-01	0.49	8.7E-02	0.27	2.5E-01	Plin5	perilipin 5
ENSMUSG00000052151	0.34	4.9E-02	0.33	1.0E-01	0.60	9.3E-03	0.55	3.6E-02	NA	NA	0.81	2.6E-03	NA	NA	Plpp2	phospholipid phosphatase 2
ENSMUSG00000032369	0.54	2.4E-02	0.36	4.8E-02	0.38	5.0E-02	1.30	2.3E-03	0.71	1.8E-04	1.66	5.9E-03	1.39	2.9E-06	Plscr1	phospholipid scramblase 1
ENSMUSG00000017754	0.57	6.5E-02	1.82	7.6E-09	1.64	9.7E-08	0.99	1.3E-01	1.54	8.2E-07	1.26	8.3E-04	2.20	9.9E-07	Pltp	phospholipid transfer protein
ENSMUSG00000073460	0.35	4.1E-01	2.27	8.5E-08	2.19	4.6E-03	3.03	2.7E-02	2.37	1.1E-04	3.78	2.7E-07	2.93	5.7E-09	Pnldc1	poly(A)-specific ribonuclease (PARN)-like domain containing 1
ENSMUSG00000068417	-0.13	3.0E-01	-0.44	4.7E-02	-0.64	5.6E-03	-0.83	3.6E-04	-0.79	1.2E-04	-1.08	1.0E-05	-1.11	4.0E-04	Pnp2	purine-nucleoside phosphorylase 2
ENSMUSG00000009739	-0.82	4.2E-02	-0.70	3.5E-02	-0.76	2.3E-02	-0.88	2.1E-02	-0.91	1.1E-02	-0.91	5.8E-02	-0.25	5.7E-01	Pou6f1	POU domain, class 6, transcription factor 1
ENSMUSG00000022383	-0.06	2.5E-01	0.21	5.8E-01	0.19	9.7E-01	0.13	6.9E-01	0.52	1.6E-01	0.32	2.5E-01	0.40	4.7E-01	Ppara	peroxisome proliferator activated receptor alpha
ENSMUSG00000002250	-0.48	3.1E-01	-0.32	2.0E-01	-0.24	8.4E-01	-0.41	2.9E-01	-0.53	1.8E-01	-0.45	5.0E-01	-0.67	3.1E-01	Ppard	peroxisome proliferator activator receptor delta
ENSMUSG00000000440	-0.12	7.8E-01	1.23	6.9E-04	1.09	2.0E-05	0.81	5.2E-03	0.33	2.5E-02	1.51	2.1E-07	1.22	3.3E-05	Pparg	peroxisome proliferator activated receptor gamma
ENSMUSG00000029167	0.27	5.5E-01	-0.25	2.5E-01	0.04	8.0E-01	-0.09	5.6E-01	0.66	8.3E-02	0.03	5.8E-01	0.18	7.3E-01	Ppargc1a	peroxisome proliferative activated receptor, gamma, coactivator 1 alpha
ENSMUSG00000033871	0.48	8.6E-01	-0.02	9.3E-01	0.06	6.5E-01	0.06	4.4E-01	0.65	4.5E-01	0.13	8.3E-01	-0.17	3.6E-01	Ppargc1b	peroxisome proliferative activated receptor, gamma, coactivator 1 beta
ENSMUSG00000029246	-0.27	2.8E-01	-0.81	1.1E-03	-0.77	1.1E-02	-0.73	2.2E-03	-0.75	8.1E-03	-0.92	4.8E-04	-1.05	4.6E-04	Ppat	phosphoribosyl pyrophosphate amidotransferase
ENSMUSG00000040225	-0.45	1.1E-02	-0.65	2.1E-03	-0.43	4.3E-02	-0.50	1.3E-02	-0.40	2.0E-02	-0.52	9.2E-02	-0.13	6.9E-01	Prrc2c	proline-rich coiled-coil 2C
ENSMUSG00000026979	0.14	1.8E-01	0.91	4.9E-04	1.19	4.9E-05	0.97	5.5E-05	0.85	9.2E-05	1.31	8.9E-07	1.70	1.3E-07	Psd4	pleckstrin and Sec7 domain containing 4
ENSMUSG00000026395	0.38	3.5E-02	0.23	7.4E-01	0.69	6.7E-02	0.14	4.8E-01	0.87	5.2E-03	1.03	2.0E-04	1.48	1.5E-06	Ptprc	protein tyrosine phosphatase, receptor type, C
ENSMUSG00000045826	0.48	4.0E-01	0.03	8.2E-01	1.02	2.3E-01	1.09	1.1E-01	0.86	2.8E-01	1.08	1.1E-01	1.02	1.7E-01	Ptprcap	protein tyrosine phosphatase, receptor type, C polypeptide-associated protein
ENSMUSG00000028909	-0.35	2.9E-01	-0.99	1.1E-02	-0.95	5.0E-03	-0.90	9.0E-03	-0.96	3.1E-02	-0.08	2.6E-01	-0.40	2.0E-01	Ptpru	protein tyrosine phosphatase, receptor type, U
ENSMUSG00000059060	0.48	5.4E-01	4.49	1.8E-04	5.16	3.7E-03	4.14	1.2E-04	3.38	1.4E-03	6.61	6.7E-04	6.46	5.9E-06	Rad51b	RAD51 paralog B
ENSMUSG00000078773	0.82	1.9E-01	1.72	5.7E-03	1.49	6.9E-03	1.45	1.9E-02	0.50	4.8E-01	1.46	1.4E-02	1.65	1.8E-02	Rad54b	RAD54 homolog B (S. cerevisiae)
ENSMUSG00000026594	0.08	5.9E-01	0.38	3.4E-02	0.49	2.0E-02	0.70	2.6E-04	0.53	9.1E-03	0.97	5.8E-04	0.75	1.6E-02	Ralgps2	Ral GEF with PH domain and SH3 binding motif 2
ENSMUSG00000057236	-0.15	6.6E-01	-0.38	6.8E-02	-0.60	4.2E-02	-0.19	6.7E-01	-0.49	6.0E-02	-0.70	2.6E-02	-0.86	1.1E-02	Rbbp4	retinoblastoma binding protein 4
ENSMUSG00000019873	0.03	8.6E-01	0.04	8.7E-01	0.15	5.5E-01	-0.04	9.0E-01	0.08	8.6E-01	0.35	4.2E-01	0.29	5.5E-01	Reep3	receptor accessory protein 3
ENSMUSG00000031387	0.15	1.7E-01	0.63	4.6E-04	0.73	1.5E-02	0.45	3.6E-02	0.78	2.0E-04	1.31	4.8E-06	1.76	7.6E-08	Renbp	renin binding protein
ENSMUSG00000021932	0.51	2.0E-02	0.48	2.2E-02	0.52	1.8E-02	0.59	3.6E-03	0.62	7.0E-04	0.92	1.0E-02	0.79	5.6E-02	Rnaseh2b	ribonuclease H2, subunit B
ENSMUSG00000028557	0.05	5.7E-01	0.27	2.2E-01	0.31	4.1E-02	0.11	2.9E-01	0.04	9.3E-01	0.35	4.9E-01	0.35	3.1E-01	Rnf11	ring finger protein 11
ENSMUSG00000020458	0.64	1.0E-03	0.81	6.8E-05	1.30	3.8E-08	0.75	3.0E-05	1.12	2.2E-04	1.37	3.9E-03	1.70	1.3E-06	Rtn4	reticulon 4
ENSMUSG00000061815	0.64	8.7E-01	2.92	7.8E-07	3.03	3.6E-08	3.10	1.7E-04	2.17	6.7E-04	3.32	1.1E-10	3.54	4.3E-09	Rufy4	RUN and FYVE domain containing 4
ENSMUSG00000015846	-0.18	2.8E-01	-0.13	5.4E-01	-0.27	2.0E-01	-0.16	2.0E-01	-0.06	7.2E-01	-0.43	3.6E-01	-0.35	3.3E-01	Rxra	retinoid X receptor alpha
ENSMUSG00000039656	-0.03	9.3E-01	0.04	6.7E-01	-0.22	4.9E-01	0.03	9.4E-01	-0.06	9.9E-01	-0.19	7.9E-01	-0.18	8.8E-01	Rxrb	retinoid X receptor beta
ENSMUSG00000015843	0.20	9.5E-01	0.77	5.0E-03	0.72	5.1E-02	0.48	3.1E-01	0.41	1.4E-01	0.54	4.1E-02	0.80	1.2E-01	Rxrg	retinoid X receptor gamma
ENSMUSG00000044080	0.39	4.2E-02	0.42	3.5E-02	0.35	6.5E-02	0.22	1.3E-01	0.28	1.9E-01	0.61	7.0E-01	0.12	1.0E+00	S100a1	S100 calcium binding protein A1

	regular chow						semisynthetic diet									
	$Ldlr^{-/-}$		$Mc4r^{mut}$		$Mc4r^{mut}$; $Ldlr^{-/-}$		wt		$Ldlr^{-/-}$		$Mc4r^{mut}$		$Mc4r^{mut}$; $Ldlr^{-/-}$			
Ensembl Gene ID	log2 fold change	p-value	log2 fold change	p-value	log2 fold change	p-value	log2 fold change	p-value	log2 fold change	p-value	log2 fold change	p-value	log2 fold change	p-value	name	description
ENSMUSG00000041959	0.60	1.9E-03	0.93	3.2E-05	0.88	2.2E-02	0.87	4.3E-04	0.85	6.2E-02	1.87	3.4E-01	1.23	1.4E-01	S100a10	S100 calcium binding protein A10 (calpactin)
ENSMUSG00000027907	0.15	1.5E-01	1.06	3.1E-03	1.08	1.0E-02	1.53	7.5E-03	1.11	8.9E-03	2.51	3.6E-06	2.49	3.9E-08	S100a11	S100 calcium binding protein A11
ENSMUSG00000042312	0.43	4.0E-02	0.42	2.4E-02	0.30	1.9E-01	0.28	2.0E-01	0.16	2.8E-01	0.37	7.4E-01	0.10	9.4E-01	S100a13	S100 calcium binding protein A13
ENSMUSG00000074457	0.27	9.3E-02	0.32	6.5E-02	0.18	3.3E-01	0.28	1.2E-01	0.36	4.8E-02	0.49	4.0E-01	0.46	4.8E-01	S100a16	S100 calcium binding protein A16
ENSMUSG00000037071	0.48	3.8E-01	1.27	5.3E-03	1.13	2.4E-02	1.22	8.6E-03	1.39	1.5E-04	1.83	7.4E-09	1.68	1.6E-06	Scd1	stearoyl-Coenzyme A desaturase 1
ENSMUSG00000025203	-0.91	1.6E-01	0.55	1.0E+00	1.14	1.0E+00	-0.32	1.8E-01	0.99	1.0E+00	0.61	8.3E-01	1.28	4.3E-01	Scd2	stearoyl-Coenzyme A desaturase 2
ENSMUSG00000025202	-0.40	1.5E-01	0.84	3.7E-01	1.05	7.1E-01	0.36	4.3E-01	1.04	4.2E-01	1.12	2.8E-01	1.23	2.2E-01	Scd3	stearoyl-coenzyme A desaturase 3
ENSMUSG00000050195	-1.70	1.7E-01	0.43	1.0E+00	0.93	7.9E-01	-0.93	1.8E-01	0.81	8.7E-01	0.57	9.0E-01	0.85	9.1E-01	Scd4	stearoyl-coenzyme A desaturase 4
ENSMUSG00000027456	NA	NA	5.31	2.2E-07	4.38	1.7E-02	5.48	1.6E-02	3.61	9.7E-02	6.73	1.8E-04	5.90	2.0E-04	Sdcbp2	syndecan binding protein (syntenin) 2
ENSMUSG00000040127	-0.78	8.1E-03	-0.91	3.0E-02	-1.22	4.2E-04	-1.53	1.2E-04	-1.33	5.4E-04	-1.93	1.6E-05	-2.20	2.9E-06	Sdr9c7	4short chain dehydrogenase/reductase family 9C, member 7
ENSMUSG00000027429	-0.25	1.7E-01	-0.40	1.1E-02	-0.70	1.3E-02	-0.65	9.3E-03	-0.71	4.3E-03	-0.79	3.8E-03	-0.76	1.7E-02	Sec23b	SEC23 homolog B, COPII coat complex component
ENSMUSG00000030082	-0.17	4.5E-01	-0.33	5.0E-02	-0.58	2.8E-02	-0.53	1.3E-02	-0.46	7.8E-02	-0.75	1.4E-02	-0.57	1.0E-01	Sec61a1	Sec61 alpha 1 subunit (S. cerevisiae)
ENSMUSG00000068874	-1.03	2.4E-02	-1.13	3.8E-02	-1.42	3.9E-03	-1.13	1.2E-02	-1.38	7.3E-03	-1.70	2.3E-03	-1.84	2.3E-03	Selenbp1	selenium binding protein 1
ENSMUSG00000068877	-1.65	1.1E-05	-2.37	6.5E-09	-2.80	2.6E-09	-1.87	2.6E-07	-2.99	5.9E-16	-4.41	5.6E-25	-4.99	4.5E-25	Selenbp2	selenium binding protein 2
ENSMUSG00000022456	NA	NA	-7.65	1.5E-02	-6.17	2.9E-02	-7.44	6.0E-03	-6.39	1.6E-02	-7.88	6.4E-03	-7.74	1.6E-02	Sept3	septin 3
ENSMUSG00000023232	-0.09	7.9E-01	1.17	4.8E-04	0.70	3.9E-02	1.01	1.4E-04	0.56	4.2E-03	1.58	6.9E-08	1.67	3.0E-07	Serinc2	serine incorporator 2
ENSMUSG00000072849	-0.34	3.2E-01	-0.98	1.3E-02	-0.97	4.6E-02	-1.47	1.5E-09	-1.32	1.4E-06	-2.90	2.2E-24	-3.15	2.4E-21	Serpina1e	serine (or cysteine) peptidase inhibitor, clade A, member 1E
ENSMUSG00000031271	0.48	5.7E-01	1.44	1.3E-03	1.46	2.5E-03	1.61	1.2E-05	1.41	1.4E-02	2.13	6.3E-02	1.29	6.0E-02	Serpina7	serine (or cysteine) peptidase inhibitor, clade A (alpha-1 antiproteinase, antitrypsin), member 7
ENSMUSG00000026315	0.69	3.2E-02	0.85	4.3E-02	1.43	2.8E-04	1.14	8.8E-04	1.70	1.1E-09	1.70	3.7E-05	2.13	3.5E-08	Serpinb8	serine (or cysteine) peptidase inhibitor, clade B, member 8
ENSMUSG00000043183	-0.09	3.5E-01	-0.44	2.5E-02	-0.58	7.8E-03	-0.47	5.6E-03	-0.92	5.0E-06	-0.76	8.0E-03	-0.93	4.1E-03	Simc1	SUMO-interacting motifs containing 1
ENSMUSG00000037902	0.25	4.1E-02	0.53	2.2E-02	0.88	1.8E-02	0.65	2.7E-02	1.08	8.1E-03	1.39	5.3E-08	2.24	1.6E-13	Sirpa	signal-regulatory protein alpha
ENSMUSG00000020063	-0.04	6.9E-01	-0.30	5.9E-01	-0.03	9.1E-01	-0.07	8.2E-01	0.05	7.8E-01	-0.22	5.3E-01	-0.31	3.6E-01	Sirt1	sirtuin 1
ENSMUSG00000015149	0.10	5.6E-01	0.12	3.7E-01	0.12	5.6E-01	0.06	8.3E-01	-0.04	8.0E-01	0.03	8.6E-01	0.05	6.4E-01	Sirt2	sirtuin 2
ENSMUSG00000025486	0.16	9.0E-01	0.24	2.8E-01	0.15	7.1E-01	0.22	4.9E-01	0.15	6.0E-01	0.02	8.7E-01	-0.20	3.5E-01	Sirt3	sirtuin 3
ENSMUSG00000029524	-0.18	5.0E-01	0.00	9.6E-01	-0.10	6.7E-01	-0.07	5.6E-01	-0.36	2.5E-01	-0.07	7.3E-01	-0.39	3.1E-01	Sirt4	sirtuin 4
ENSMUSG00000054021	0.12	6.4E-01	0.48	5.7E-02	0.32	2.4E-01	0.49	3.9E-02	0.33	5.9E-02	0.62	2.7E-02	0.67	3.0E-02	Sirt5	sirtuin 5
ENSMUSG00000034748	-0.28	4.9E-01	-0.26	4.4E-01	-0.25	4.4E-01	-0.22	5.8E-01	-0.22	8.9E-01	0.14	5.0E-01	-0.28	9.1E-01	Sirt6	sirtuin 6
ENSMUSG00000025138	0.02	7.5E-01	0.06	3.7E-01	-0.08	8.3E-01	0.08	8.2E-01	-0.13	7.9E-01	-0.14	8.9E-01	-0.20	6.3E-01	Sirt7	sirtuin 7
ENSMUSG00000036309	0.09	7.1E-01	0.24	1.4E-01	0.24	1.1E-01	0.09	3.4E-01	0.13	7.0E-01	0.48	4.6E-01	0.15	8.5E-01	Skp1a	S-phase kinase-associated protein 1A
ENSMUSG00000045775	2.42	1.7E-03	2.45	7.3E-10	3.24	3.5E-07	2.63	8.1E-04	2.99	4.7E-04	3.11	1.7E-05	3.73	1.5E-11	Slc16a5	solute carrier family 16 (monocarboxylic acid transporters), member 5
ENSMUSG00000020102	0.30	7.7E-01	0.76	5.6E-02	1.06	1.5E-03	0.61	2.7E-02	0.85	9.6E-04	1.55	9.6E-07	1.38	2.3E-04	Slc16a7	solute carrier family 16 (monocarboxylic acid transporters), member 7
ENSMUSG00000036083	-0.30	6.2E-02	-0.54	1.8E-03	-0.58	5.6E-03	-0.48	1.2E-02	-0.61	5.1E-04	-0.87	7.1E-04	-1.00	3.8E-04	Slc17a3	solute carrier family 17 (sodium phosphate), member 3
ENSMUSG00000005089	-0.33	5.3E-02	-0.85	1.4E-04	-0.95	5.5E-06	-0.77	1.1E-04	-0.85	2.2E-05	-0.82	1.1E-02	-1.27	2.4E-05	Slc1a2	solute carrier family 1 (glial high affinity glutamate transporter), member 2
ENSMUSG00000052562	-0.12	3.4E-01	-0.56	1.4E-03	-0.51	3.9E-02	-0.51	2.9E-02	-0.65	1.3E-04	-1.30	1.6E-06	-1.54	1.2E-07	Slc22a30	solute carrier family 22, member 30
ENSMUSG00000042202	-0.39	4.1E-02	-0.43	2.4E-02	-0.53	1.5E-02	-0.69	2.5E-04	-0.47	7.2E-03	-0.85	1.3E-03	-0.77	5.7E-03	Slc35e2	solute carrier family 35, member E2

	regular chow						semisynthetic diet									
	Ldlr$^{-/-}$		Mc4r^{mut}		Mc4r^{mut}; Ldlr$^{-/-}$		wt		Ldlr$^{-/-}$		Mc4r^{mut}		Mc4r^{mut}; Ldlr$^{-/-}$			
Ensembl Gene ID	log2 fold change	p-value	log2 fold change	p-value	log2 fold change	p-value	log2 fold change	p-value	log2 fold change	p-value	log2 fold change	p-value	log2 fold change	p-value	name	description
ENSMUSG00000042195	1.22	2.1E-01	2.51	1.0E-03	2.35	1.0E-02	3.30	1.7E-09	3.44	1.1E-09	4.00	4.4E-06	4.39	3.8E-14	Slc35f2	solute carrier family 35, member F2
ENSMUSG00000039878	1.04	1.1E-01	2.97	5.0E-04	3.22	5.7E-09	2.04	2.7E-05	2.33	1.3E-04	3.07	6.0E-04	3.77	2.8E-12	Slc39a5	solute carrier family 39 (metal ion transporter), member 5
ENSMUSG00000072620	0.50	2.9E-02	0.73	2.4E-02	1.13	5.0E-03	1.09	9.5E-04	1.24	8.7E-05	1.84	9.2E-02	2.08	4.6E-05	Slfn2	schlafen 2
ENSMUSG00000000204	0.85	1.7E-01	1.88	5.0E-02	2.06	9.9E-03	1.62	4.6E-02	1.89	6.0E-03	2.77	2.7E-02	2.84	1.3E-05	Slfn4	schlafen 4
ENSMUSG00000071669	-1.00	9.2E-03	-1.31	1.5E-03	-1.36	1.0E-03	-1.18	3.4E-03	-1.08	4.6E-03	-2.23	8.4E-06	-1.73	2.1E-04	Snx29	sorting nexin 29
ENSMUSG00000041540	-0.68	5.3E-03	-0.38	3.6E-02	-0.49	1.2E-02	-0.78	1.2E-04	-0.60	6.1E-04	-0.44	6.2E-02	-0.75	7.1E-03	Sox5	SRY (sex determining region Y)-box 5
ENSMUSG00000074476	0.36	1.8E-01	0.60	3.0E-02	0.76	9.7E-03	0.62	2.5E-02	0.94	3.3E-04	0.59	4.2E-02	0.76	8.7E-02	Spc24	SPC24, NDC80 kinetochore complex component, homolog (S. cerevisiae)
ENSMUSG00000005233	0.51	2.9E-01	0.84	9.6E-04	1.22	2.5E-03	0.93	1.1E-05	0.74	3.7E-02	1.85	3.1E-05	1.56	5.8E-04	Spc25	SPC25, NDC80 kinetochore complex component, homolog (S. cerevisiae)
ENSMUSG00000040761	-0.38	4.2E-02	-0.34	3.7E-02	-0.44	2.8E-02	-0.52	1.5E-03	-0.40	1.5E-02	-0.78	7.5E-03	-0.23	4.4E-01	Spen	SPEN homolog, transcriptional regulator (Drosophila)
ENSMUSG00000055561	1.83	2.2E-02	1.37	3.5E-02	1.36	2.0E-02	2.17	5.4E-03	2.33	1.9E-04	1.47	1.1E-02	1.04	9.2E-02	Spink5	serine peptidase inhibitor, Kazal type 5
ENSMUSG00000020538	-0.21	4.3E-01	0.45	4.3E-01	0.14	6.3E-01	0.77	1.1E-01	0.88	2.6E-02	0.85	8.2E-03	0.60	1.1E-01	Srebf1	sterol regulatory element binding transcription factor 1
ENSMUSG00000022463	-1.67	6.6E-02	-1.45	6.8E-02	-1.75	3.4E-02	-1.95	2.5E-02	-1.89	4.6E-02	-1.94	4.1E-02	-1.79	5.7E-02	Srebf2	sterol regulatory element binding factor 2
ENSMUSG00000021134	-0.13	6.4E-01	-0.03	7.1E-01	0.06	1.0E+00	0.15	4.1E-01	-0.15	6.4E-01	0.56	1.2E-02	0.24	3.7E-01	Srsf5	serine/arginine-rich splicing factor 5
ENSMUSG00000032802	0.31	8.7E-02	0.91	2.9E-03	0.74	3.3E-03	1.22	6.7E-02	0.58	4.0E-03	1.59	7.0E-05	1.50	3.7E-06	Srxn1	sulfiredoxin 1 homolog (S. cerevisiae)
ENSMUSG00000029254	1.55	5.5E-05	2.84	1.5E-04	3.48	8.6E-06	3.80	5.1E-13	4.36	8.7E-08	4.36	1.8E-07	4.20	6.4E-09	Stap1	signal transducing adaptor family member 1
ENSMUSG00000006800	1.02	1.3E-03	1.14	7.3E-05	1.57	2.6E-05	1.45	1.2E-03	1.78	5.2E-09	2.02	3.5E-11	2.14	4.5E-10	Sulf2	sulfatase 2
ENSMUSG00000023122	0.91	2.1E-01	1.25	3.6E-02	1.52	1.2E-03	1.59	6.8E-05	2.31	1.0E-08	2.17	1.0E-03	2.84	3.7E-09	Sult1c2	sulfotransferase family, cytosolic, 1C, member 2
ENSMUSG00000038576	-0.58	3.6E-03	-1.06	1.6E-05	-2.30	2.6E-16	-1.66	5.1E-12	-2.07	4.3E-24	-3.12	2.1E-25	-3.64	1.9E-26	Susd4	sushi domain containing 4
ENSMUSG00000031357	0.09	8.3E-01	0.03	8.5E-01	0.16	3.7E-01	-0.08	9.7E-01	0.13	6.2E-01	0.20	8.2E-01	0.12	9.2E-01	Syap1	synapse associated protein 1
ENSMUSG00000022340	1.55	1.6E-01	1.55	2.5E-02	2.86	1.9E-04	2.15	1.5E-02	1.98	1.2E-01	3.37	1.5E-06	4.40	1.3E-12	Sybu	syntabulin (syntaxin-interacting)
ENSMUSG00000052302	-0.59	4.6E-02	-1.37	2.1E-04	-1.45	1.6E-05	-0.93	1.2E-03	-0.52	4.0E-02	-1.32	1.8E-03	-1.15	8.4E-03	Tbc1d30	TBC1 domain family, member 30
ENSMUSG00000046603	-0.37	1.1E-01	-0.39	4.3E-02	-0.61	4.4E-02	-0.64	7.9E-03	-0.53	1.1E-02	-0.74	1.1E-02	-0.76	1.7E-02	Tcaim	T cell activation inhibitor, mitochondrial
ENSMUSG00000051579	0.86	3.9E-02	1.87	2.7E-05	2.23	1.3E-04	1.56	7.8E-06	2.22	1.3E-04	3.04	8.4E-03	2.82	6.6E-07	Tceal8	transcription elongation factor A (SII)-like 8
ENSMUSG00000047347	-1.03	3.9E-02	-1.29	5.0E-02	-1.25	9.2E-03	-0.87	3.1E-02	-1.46	1.1E-02	-1.63	1.1E-02	-2.20	4.5E-03	Tdg-ps	thymine DNA glycosylase, pseudogene
ENSMUSG00000020778	0.06	2.6E-01	0.57	6.0E-03	0.68	9.5E-03	0.86	2.9E-04	0.65	2.5E-02	1.06	1.5E-02	1.04	3.2E-02	Ten1	TEN1 telomerase capping complex subunit
ENSMUSG00000003923	0.06	8.7E-01	0.07	7.2E-01	0.07	5.4E-01	-0.01	1.0E+00	0.13	5.1E-01	0.03	9.2E-01	0.06	8.7E-01	Tfam	transcription factor A, mitochondrial
ENSMUSG00000002603	0.20	3.1E-01	0.62	2.4E-02	0.72	1.5E-02	0.64	6.4E-03	0.67	6.2E-03	0.78	3.4E-03	1.32	3.0E-05	Tgfb1	transforming growth factor, beta 1
ENSMUSG00000024251	-0.54	6.3E-03	-0.43	3.4E-02	-0.56	2.6E-02	-0.44	2.7E-02	-0.44	8.5E-02	-0.48	2.8E-01	-0.38	4.0E-01	Thada	thyroid adenoma associated
ENSMUSG00000049109	0.62	7.3E-01	2.64	3.9E-03	2.29	7.2E-03	2.11	8.8E-03	1.20	3.2E-02	3.73	1.4E-14	3.30	2.2E-04	Themis	thymocyte selection associated
ENSMUSG00000001131	1.08	1.2E-01	1.55	3.7E-02	2.50	5.9E-03	2.39	1.1E-02	2.59	1.0E-02	4.50	3.2E-02	4.71	6.7E-19	Timp1	tissue inhibitor of metalloproteinase 1
ENSMUSG00000034371	-0.06	3.6E-01	0.48	2.7E-01	0.35	6.9E-01	0.19	8.2E-01	0.34	5.4E-02	0.51	9.3E-02	0.43	2.7E-01	Tkfc	triokinase, FMN cyclase
ENSMUSG00000044827	1.37	2.5E-02	1.59	3.4E-02	2.35	8.8E-03	2.09	9.6E-04	2.39	9.6E-03	2.95	4.1E-07	3.60	9.3E-10	Tlr1	toll-like receptor 1
ENSMUSG00000062545	-0.14	4.8E-01	1.82	5.0E-07	1.26	6.0E-04	1.39	2.6E-03	1.09	7.1E-05	2.17	7.4E-08	1.94	1.5E-07	Tlr12	toll-like receptor 12
ENSMUSG00000027801	0.46	1.5E-03	0.28	3.0E-02	0.49	2.0E-02	0.62	4.2E-02	0.42	3.3E-02	1.06	1.5E-04	0.90	6.8E-04	Tm4sf4	transmembrane 4 superfamily member 4
ENSMUSG00000036151	0.97	6.6E-06	0.89	1.0E-04	1.13	1.3E-05	0.61	4.9E-02	1.00	1.6E-06	0.71	7.8E-03	1.01	2.5E-03	Tm6sf2	transmembrane 6 superfamily member 2

	regular chow						semisynthetic diet									
	Ldlr$^{-/-}$		Mc4r^{mut}		Mc4r^{mut}; Ldlr$^{-/-}$		wt		Ldlr$^{-/-}$		Mc4r^{mut}		Mc4r^{mut}; Ldlr$^{-/-}$			
**Ensembl Gene ID**	**log2 fold change**	**p-value**	**log2 fold change**	**p-value**	**log2 fold change**	**p-value**	**log2 fold change**	**p-value**	**log2 fold change**	**p-value**	**log2 fold change**	**p-value**	**log2 fold change**	**p-value**	**name**	**description**
ENSMUSG00000026109	-0.57	2.0E-01	-4.04	5.0E-07	-2.22	1.8E-03	-2.17	3.1E-03	-2.75	2.7E-05	-4.11	4.9E-08	-5.39	5.6E-09	Tmeff2	transmembrane protein with EGF-like and two follistatin-like domains 2
ENSMUSG00000039886	0.29	3.2E-02	0.59	5.5E-05	0.46	1.3E-02	0.66	3.5E-04	0.45	8.2E-03	0.79	4.0E-03	0.53	4.5E-02	Tmem120a	transmembrane protein 120A
ENSMUSG00000009035	0.30	4.8E-01	0.73	1.1E-02	0.63	4.5E-02	0.73	1.4E-02	0.66	5.7E-03	1.11	1.2E-04	1.19	9.4E-04	Tmem184b	transmembrane protein 184b
ENSMUSG00000022856	0.19	1.9E-01	0.45	2.3E-02	0.61	2.1E-02	0.68	1.1E-02	0.59	9.2E-03	0.70	1.4E-03	0.80	8.5E-03	Tmem41a	transmembrane protein 41a
ENSMUSG00000048772	0.30	2.3E-01	0.65	2.5E-03	0.63	2.3E-02	0.68	4.5E-02	0.48	1.1E-02	0.74	1.5E-02	0.82	1.9E-02	Tmem53	transmembrane protein 53
ENSMUSG00000010307	0.60	1.6E-02	0.66	2.4E-02	1.30	4.4E-05	1.82	4.7E-05	2.09	1.8E-05	2.15	1.1E-11	2.62	3.0E-12	Tmem86a	transmembrane protein 86A
ENSMUSG00000035413	0.62	4.9E-03	1.16	2.0E-06	0.93	3.1E-04	0.63	1.7E-02	0.45	1.9E-02	0.81	2.4E-03	0.87	1.8E-03	Tmem98	transmembrane protein 98
ENSMUSG00000068105	-7.41	5.6E-03	-6.67	2.5E-02	-5.88	3.2E-02	-7.26	6.6E-03	-5.77	2.4E-02	-7.00	9.4E-03	-7.53	1.7E-02	Tnfrsf13c	tumor necrosis factor receptor superfamily, member 13c
ENSMUSG00000047888	-0.45	4.0E-03	-0.42	1.7E-02	-0.47	1.6E-02	-0.69	2.0E-04	-0.61	1.4E-03	-0.59	3.2E-02	-0.55	4.2E-02	Tnrc6b	trinucleotide repeat containing 6b
ENSMUSG00000026849	0.20	2.4E-01	0.45	1.8E-02	0.41	4.5E-02	0.67	5.3E-04	0.65	3.8E-04	0.80	1.8E-03	0.73	2.3E-02	Tor1a	torsin family 1, member A (torsin A)
ENSMUSG00000030894	0.00	9.7E-01	0.02	1.0E+00	0.12	7.7E-01	0.09	7.7E-01	-0.02	8.5E-01	0.37	1.4E-01	0.38	2.1E-01	Tpp1	tripeptidyl peptidase I
ENSMUSG00000032098	0.39	3.5E-02	1.64	6.5E-07	1.39	1.5E-03	0.57	7.1E-03	0.42	4.8E-02	1.00	3.8E-05	1.75	2.0E-08	Treh	trehalase (brush-border membrane glycoprotein)
ENSMUSG00000030555	0.14	9.5E-01	0.51	1.3E-01	0.68	1.9E-02	0.33	2.9E-01	0.54	6.1E-03	0.65	5.3E-02	0.59	1.5E-01	Ttc23	tetratricopeptide repeat domain 23
ENSMUSG00000028555	0.86	1.8E-01	2.02	4.4E-03	1.98	1.9E-02	2.18	2.9E-02	2.67	2.6E-04	4.07	4.1E-34	4.34	1.5E-24	Ttc39a	tetratricopeptide repeat domain 39A
ENSMUSG00000053841	-0.58	4.1E-02	-0.54	8.9E-03	-0.65	1.6E-02	-0.48	2.4E-02	-0.73	1.1E-03	-0.78	2.4E-02	-0.74	8.8E-02	Txlna	taxilin alpha
ENSMUSG00000030579	0.64	1.7E-03	0.57	1.4E-02	1.09	2.9E-03	0.77	1.2E-04	1.23	4.5E-06	1.52	6.9E-02	2.09	1.5E-02	Tyrobp	TYRO protein tyrosine kinase binding protein
ENSMUSG00000064594	-1.12	2.5E-03	-1.54	5.4E-04	-2.77	1.9E-06	-1.82	9.8E-06	-2.36	2.5E-08	-3.21	7.0E-13	-3.99	2.5E-14	U1	U1 spliceosomal RNA
ENSMUSG00000027078	0.23	1.9E-01	0.41	2.4E-02	0.45	4.2E-02	0.51	8.7E-03	0.45	9.7E-03	0.49	7.9E-02	0.38	1.3E-01	Ube2l6	ubiquitin-conjugating enzyme E2L 6
ENSMUSG00000033685	0.03	4.6E-01	0.81	5.3E-03	1.06	4.4E-02	0.53	4.9E-03	0.48	1.3E-02	1.06	7.6E-03	1.45	1.5E-05	Ucp2	uncoupling protein 2 (mitochondrial, proton carrier)
ENSMUSG00000037470	-0.09	7.5E-01	-0.51	1.4E-02	-0.51	4.9E-02	-0.79	2.3E-04	-0.57	8.4E-03	-0.88	5.2E-03	-0.70	2.3E-02	Uggt1	UDP-glucose glycoprotein glucosyltransferase 1
ENSMUSG00000035836	-0.13	4.0E-01	-0.76	1.0E-04	-0.87	1.3E-03	-0.64	3.5E-03	-0.69	2.3E-04	-1.40	1.4E-07	-1.59	5.7E-08	Ugt2b1	UDP glucuronosyltransferase 2 family, polypeptide B1
ENSMUSG00000035811	-0.43	9.6E-03	-0.51	6.3E-04	-0.55	6.3E-03	-0.74	2.0E-03	-0.88	1.3E-06	-0.54	6.3E-03	-0.53	1.1E-02	Ugt2b35	UDP glucuronosyltransferase 2 family, polypeptide B35
ENSMUSG00000002058	0.47	1.2E-01	1.68	2.0E-05	1.25	4.5E-05	1.00	3.3E-05	0.78	1.2E-03	1.41	1.3E-04	1.49	2.6E-05	Unc119	unc-119 lipid binding chaperone
ENSMUSG00000037440	0.34	9.9E-01	1.89	4.4E-05	2.06	1.1E-03	1.56	2.2E-04	1.67	4.7E-06	2.21	4.5E-13	2.32	3.3E-10	Vnn1	vanin 1
ENSMUSG00000029462	0.14	5.5E-01	0.33	9.9E-02	0.25	1.9E-01	0.26	9.6E-02	0.41	7.5E-02	0.85	4.7E-01	0.54	4.0E-01	Vps29	VPS29 retromer complex component
ENSMUSG00000025722	-0.08	8.3E-01	0.41	1.3E-02	0.41	4.8E-02	0.64	2.6E-03	0.55	7.9E-03	0.86	4.5E-04	0.93	3.5E-03	Wdr73	WD repeat domain 73
ENSMUSG00000017723	0.45	5.3E-02	2.00	1.4E-06	1.97	2.3E-05	0.99	3.5E-02	1.70	1.9E-02	2.93	1.7E-02	3.43	3.6E-07	Wfdc2	WAP four-disulfide core domain 2
ENSMUSG00000035112	-0.33	1.3E-01	-1.77	1.5E-04	-1.45	4.5E-03	-0.88	7.8E-03	-1.53	2.5E-03	-1.84	6.1E-04	-2.31	1.3E-05	Wnk4	WNK lysine deficient protein kinase 4
ENSMUSG00000022708	-0.30	7.3E-02	-0.51	1.8E-02	-0.52	1.5E-02	-0.43	4.4E-02	-0.43	4.0E-02	-0.90	6.4E-04	-0.89	6.2E-03	Zbtb20	zinc finger and BTB domain containing 20
ENSMUSG00000040721	-0.62	2.3E-02	-0.84	2.8E-03	-0.74	9.1E-03	-0.83	4.1E-04	-1.03	8.0E-05	-1.11	2.3E-03	-0.84	6.4E-02	Zfhx2	zinc finger homeobox 2
ENSMUSG00000069755	-0.51	5.2E-02	-0.80	2.3E-02	-0.28	3.4E-02	-0.39	4.5E-02	-0.60	5.6E-03	-0.87	3.2E-04	-0.86	1.0E-03	Zfp125	zinc finger protein 125
ENSMUSG00000035877	-0.31	8.1E-02	-0.53	8.3E-03	-0.75	1.0E-03	-0.69	3.2E-04	-0.55	5.4E-03	-0.93	2.2E-03	-0.93	3.8E-03	Zhx3	zinc fingers and homeoboxes 3
ENSMUSG00000041961	-0.40	4.9E-02	-0.56	4.0E-02	-0.43	4.9E-02	-0.37	4.5E-02	-0.43	1.2E-01	-0.35	1.5E-01	-0.35	2.1E-01	Znrf3	zinc and ring finger 3
