# Supplementary material for: Altered hepatic lipid metabolism in mice lacking both the melanocortin type 4 receptor and low density lipoprotein receptor
Source: PLoS One. 2017 Feb 16;12(2):e0172000. doi: 10.1371/journal.pone.0172000 (PMC5313158; doi:10.1371/journal.pone.0172000)
Supplement: S6 Table — Gene lists were generated comprising only genes that were significantly regulated in all groups fed with semisynthetic diet, but in none of the regular chow fed mice. Similar lists for Mc4rmut and Ldlr-/- background were created respectively. Genes with a p-value < 0.05 were considered statistically significant. Log2 fold changes and p-values are given. (PDF) [file pone.0172000.s009.pdf]

S6 Table. Genes specifically regulated in either the $Mc4r^{mut}$ or the $Ldlr^{-/-}$ background or under semisynthetic diet.

$Mc4r^{mut}$ specific genes

	regular chow						semisynthetic diet									
	$Ldlr^{-/-}$		$Mc4r^{mut}$		$Mc4r^{mut}$; $Ldlr^{-/-}$		wt		$Ldlr^{-/-}$		$Mc4r^{mut}$		$Mc4r^{mut}$; $Ldlr^{-/-}$			
**Ensembl Gene ID**	**log2 fold change**	**p-value**	**log2 fold change**	**p-value**	**log2 fold change**	**p-value**	**log2 fold change**	**p-value**	**log2 fold change**	**p-value**	**log2 fold change**	**p-value**	**log2 fold change**	**p-value**	**name**	**description**
ENSMUSG00000053553	-0.90	1.7E-01	-1.22	8.6E-03	-2.14	2.3E-04	0.14	3.9E-01	-0.74	2.3E-01	-1.69	3.7E-03	-1.50	1.1E-02	3110082I17Rik	RIKEN cDNA 3110082I17 gene
ENSMUSG00000087611	0.68	6.4E-01	1.71	4.8E-02	2.12	6.7E-03	1.54	5.7E-02	1.24	1.5E-01	2.50	3.0E-04	2.50	8.4E-04	4930458D05Rik	RIKEN cDNA 4930458D05 gene
ENSMUSG00000022994	-0.02	4.7E-01	0.63	9.0E-03	0.65	4.7E-02	0.56	5.5E-02	0.52	6.6E-02	1.06	8.8E-05	0.87	5.7E-03	Adcy6	adenylate cyclase 6
ENSMUSG00000045730	0.71	1.0E-01	1.19	3.0E-02	1.29	9.2E-03	0.95	9.5E-02	0.77	9.5E-02	2.06	3.0E-05	2.11	4.4E-05	Adrb2	adrenergic receptor, beta 2
ENSMUSG00000030762	0.37	5.3E-01	1.04	2.7E-02	0.97	3.7E-02	0.57	3.8E-01	0.82	5.3E-02	1.33	1.5E-02	1.01	3.1E-02	Aqp8	aquaporin 8
ENSMUSG00000022947	0.59	4.3E-01	2.29	8.3E-03	2.30	4.2E-03	1.00	2.0E-01	0.98	2.2E-01	3.11	1.4E-03	3.45	8.1E-05	Cbr3	carbonyl reductase 3
ENSMUSG00000038903	0.05	8.6E-01	1.20	1.9E-03	1.28	2.8E-03	0.57	1.4E-01	0.03	8.5E-01	1.58	7.7E-03	1.66	3.3E-04	Ccdc68	coiled-coil domain containing 68
ENSMUSG00000006398	0.92	1.9E-01	1.96	2.1E-02	2.87	7.9E-03	2.86	6.7E-02	1.75	1.4E-01	2.89	2.9E-02	3.47	8.0E-05	Cdc20	cell division cycle 20
ENSMUSG00000023505	0.70	4.3E-01	1.43	6.9E-04	1.98	1.5E-02	2.24	9.3E-02	1.48	9.6E-02	2.49	2.2E-02	2.62	5.9E-04	Cdca3	cell division cycle associated 3
ENSMUSG00000019942	0.91	6.2E-02	1.32	3.3E-02	2.13	1.1E-02	2.11	1.5E-01	1.54	1.6E-01	2.40	5.7E-04	2.54	4.3E-04	Cdk1	cyclin-dependent kinase 1
ENSMUSG00000024542	0.02	8.1E-01	1.24	4.4E-04	1.35	1.3E-04	0.47	1.1E-01	0.46	1.4E-01	1.67	2.3E-04	1.78	1.3E-06	Cep192	centrosomal protein 192
ENSMUSG00000046324	-0.07	4.7E-01	0.70	4.9E-03	0.62	2.0E-02	0.23	3.4E-01	0.16	5.0E-01	0.90	2.5E-03	1.06	6.0E-04	Ermp1	endoplasmic reticulum metallopeptidase 1
ENSMUSG00000085023	0.18	1.3E-01	0.76	5.5E-05	0.43	3.6E-02	0.37	1.5E-01	0.21	7.1E-02	0.65	4.4E-03	0.69	7.8E-03	Gm12744	predicted gene 12744
ENSMUSG00000087435	0.83	3.0E-01	1.47	1.9E-02	1.73	5.0E-03	1.08	1.2E-01	1.03	1.4E-01	1.59	4.6E-03	1.60	1.8E-02	Gm16323	predicted gene 16323
ENSMUSG00000087904	-0.29	1.3E-01	-1.05	9.9E-04	-1.86	1.4E-05	-0.09	7.5E-01	-0.61	7.0E-02	-2.45	5.9E-11	-3.56	1.9E-14	Gm22036	predicted gene, 22036
ENSMUSG00000024697	-0.49	5.1E-02	-1.18	1.5E-04	-1.22	3.2E-05	-0.55	3.6E-01	-0.42	2.2E-01	-1.23	5.7E-04	-1.57	8.6E-05	Gna14	guanine nucleotide binding protein, alpha 14
ENSMUSG00000058135	0.24	3.3E-01	1.03	3.3E-03	0.71	1.1E-03	0.18	9.7E-01	0.04	6.7E-01	1.07	2.9E-04	1.08	3.9E-03	Gstm1	glutathione S-transferase, mu 1
ENSMUSG00000027890	0.25	6.0E-01	0.92	9.3E-03	0.68	1.9E-02	-0.09	3.1E-01	-0.05	6.4E-01	0.87	1.5E-02	0.62	3.4E-02	Gstm4	glutathione S-transferase, mu 4
ENSMUSG00000022096	-0.60	3.2E-01	1.62	1.2E-07	1.43	2.0E-03	1.62	1.4E-01	0.42	9.1E-02	1.69	2.5E-03	2.20	5.9E-06	Hr	hairless
ENSMUSG00000039323	-0.06	8.6E-01	-1.31	1.1E-06	-1.36	3.0E-04	-0.06	9.5E-01	-0.48	3.6E-01	-1.23	4.5E-04	-2.29	2.8E-10	Igfbp2	insulin-like growth factor binding protein 2
ENSMUSG00000022790	0.03	1.0E00	0.81	1.8E-03	0.64	1.3E-02	0.13	3.3E-01	0.18	2.2E-01	0.76	2.0E-02	1.06	6.2E-04	Igsf11	immunoglobulin superfamily, member 11
ENSMUSG00000073856	0.92	2.7E-01	3.23	3.4E-04	2.68	2.0E-04	2.20	6.0E-02	1.16	1.8E-01	2.99	8.7E-06	2.78	2.2E-04	Iqck	IQ motif containing K
ENSMUSG00000040675	1.17	2.1E-01	2.57	9.1E-03	2.82	1.5E-04	2.39	1.0E-01	2.98	1.3E-01	3.36	1.1E-05	3.47	3.2E-08	Mthfd1l	methylenetetrahydrofolate dehydrogenase (NADP+ dependent) 1-like
ENSMUSG00000026622	0.73	1.8E-01	1.00	2.7E-02	1.39	2.0E-04	1.23	7.7E-02	0.78	8.7E-02	1.55	3.5E-04	1.64	1.4E-04	Nek2	NIMA (never in mitosis gene a)-related expressed kinase 2
ENSMUSG00000030562	-0.13	3.3E-01	-0.56	1.7E-03	-0.78	1.8E-03	-0.01	8.1E-01	-0.17	3.7E-01	-0.63	2.3E-02	-1.15	1.0E-04	Nox4	NADPH oxidase 4
ENSMUSG00000032420	0.25	4.5E-01	0.91	1.6E-03	1.49	2.8E-05	-0.14	9.5E-01	0.23	5.0E-01	0.87	1.1E-02	1.52	2.2E-06	Nt5e	5' nucleotidase, ecto
ENSMUSG00000021699	0.45	2.8E-01	1.42	6.1E-03	1.22	2.8E-03	0.65	2.3E-01	0.52	1.2E-01	1.78	6.3E-05	1.62	2.0E-04	Pde4d	phosphodiesterase 4D, cAMP specific
ENSMUSG00000002831	0.13	8.8E-01	1.78	4.6E-03	1.63	1.6E-05	1.16	1.6E-01	0.67	8.6E-01	2.22	7.2E-07	1.89	2.0E-05	Plin4	perilipin 4
ENSMUSG00000039601	0.16	9.6E-01	0.96	1.5E-02	1.21	1.1E-02	0.34	4.2E-01	0.40	2.0E-01	1.17	8.6E-04	1.38	5.7E-05	Rcan2	regulator of calcineurin 2
ENSMUSG00000022883	0.30	4.1E-01	0.85	3.8E-02	1.15	1.2E-04	-0.38	2.2E-01	0.41	9.7E-02	0.69	2.0E-02	1.43	1.3E-05	Robo1	roundabout guidance receptor 1

ENSMUSG00000021838	0.19	1.8E-01	0.80	3.1E-02	0.67	9.7E-03	0.39	8.4E-02	0.26	1.6E-01	1.10	2.0E-04	1.20	8.5E-05	Samd4	sterile alpha motif domain containing 4
ENSMUSG00000026249	-0.25	2.3E-01	-1.18	1.1E-03	-1.70	5.7E-05	-0.32	4.8E-01	-0.59	5.5E-02	-2.05	1.1E-10	-2.16	1.3E-07	Serpine2	serine (or cysteine) peptidase inhibitor, clade E, member 2
ENSMUSG00000025792	0.08	7.8E-01	0.92	1.5E-04	0.70	2.0E-02	0.28	2.8E-01	0.25	6.9E-02	0.49	4.4E-02	0.55	4.4E-02	Slc25a10	solute carrier family 25 (mitochondrial carrier, dicarboxylate transporter), member 10
ENSMUSG00000041698	-0.31	5.3E-02	-1.05	6.3E-04	-1.97	4.0E-06	-0.28	2.5E-01	-0.47	1.0E-01	-2.42	2.4E-16	-4.12	2.5E-33	Slco1a1	solute carrier organic anion transporter family, member 1a1
ENSMUSG00000063975	-1.11	6.2E-02	-2.08	4.7E-02	-2.01	1.4E-02	-0.80	1.4E-01	-0.93	6.4E-02	-3.93	4.7E-04	-2.39	2.5E-03	Slco1a5	solute carrier organic anion transporter family, member 1a5
ENSMUSG00000037379	-0.32	2.1E-01	1.17	7.0E-04	0.90	1.1E-02	0.88	1.2E-01	0.04	9.4E-01	1.49	3.8E-03	1.44	3.2E-03	Spon2	spondin 2, extracellular matrix protein
ENSMUSG00000024427	-0.51	1.0E-01	-0.99	9.2E-03	-1.00	3.0E-03	-0.27	3.1E-01	0.02	8.5E-01	-0.90	3.4E-02	-0.91	4.8E-02	Spry4	sprouty homolog 4 (Drosophila)
ENSMUSG00000028832	0.12	1.5E-01	0.38	1.1E-02	0.66	1.2E-02	0.83	6.9E-02	0.12	3.7E-01	0.88	1.4E-02	1.06	9.6E-05	Stmn1	stathmin 1
ENSMUSG00000030378	-0.01	5.9E-01	-0.62	9.7E-03	-0.81	2.5E-03	-0.51	1.1E-01	-0.20	3.6E-01	-1.63	5.3E-09	-2.07	2.4E-13	Sult2a8	sulfotransferase family 2A, dehydroepiandrosterone (DHEA)-preferring, member 8
ENSMUSG00000031255	0.14	9.2E-01	0.91	1.9E-02	0.80	1.9E-02	0.07	8.0E-01	0.60	1.2E-01	0.98	9.7E-03	0.95	1.4E-02	Sytl4	synaptotagmin-like 4
ENSMUSG00000054453	0.91	1.6E-01	2.49	8.1E-04	3.42	5.3E-05	1.01	3.7E-01	2.22	1.4E-01	2.33	1.6E-02	3.44	2.0E-06	Sytl5	synaptotagmin-like 5
ENSMUSG00000037613	1.39	1.2E-01	1.88	3.9E-02	2.24	9.1E-03	0.93	2.9E-01	1.40	8.9E-02	3.04	5.4E-03	3.19	3.0E-05	Tnfrsf23	tumor necrosis factor receptor superfamily, member 23
ENSMUSG00000027469	0.82	1.3E-01	1.25	2.8E-02	2.05	3.5E-02	2.11	1.9E-01	1.21	1.7E-01	2.94	1.5E-02	2.90	1.6E-04	Tpx2	TPX2, microtubule-associated
ENSMUSG00000050663	0.18	7.0E-01	-0.62	2.3E-02	-0.82	3.9E-02	-0.62	7.2E-02	0.01	5.9E-01	-1.33	2.0E-03	-1.65	5.5E-06	Trhde	TRH-degrading enzyme
ENSMUSG00000021569	1.17	1.5E-01	1.52	2.3E-02	1.42	2.7E-02	0.96	1.0E-01	0.45	3.4E-01	2.23	9.4E-04	1.47	2.3E-02	Trip13	thyroid hormone receptor interactor 13
ENSMUSG00000058672	0.27	4.1E-01	1.25	4.5E-02	0.87	3.0E-02	1.12	5.6E-02	0.21	4.7E-01	1.51	1.5E-02	1.30	1.1E-02	Tubb2a	tubulin, beta 2A class IIA
ENSMUSG00000026956	0.19	3.8E-01	1.14	2.1E-03	0.93	4.2E-02	0.95	6.1E-02	0.95	7.3E-02	2.32	1.0E-06	2.58	5.3E-14	Uap1l1	UDP-N-acteylglucosamine pyrophosphorylase 1-like 1

Ldlr$^{-/-}$ specific genes

	regular chow						semisynthetic diet									
	Ldlr$^{-/-}$		Mc4r^{mut}		Mc4r^{mut}; Ldlr$^{-/-}$		wt		Ldlr$^{-/-}$		Mc4r^{mut}		Mc4r^{mut}; Ldlr$^{-/-}$			
**Ensembl Gene ID**	**log2 fold change**	**p-value**	**log2 fold change**	**p-value**	**log2 fold change**	**p-value**	**log2 fold change**	**p-value**	**log2 fold change**	**p-value**	**log2 fold change**	**p-value**	**log2 fold change**	**p-value**	**name**	**description**
ENSMUSG00000090307	1.13	1.0E-02	0.79	3.2E-01	1.47	8.8E-03	0.76	1.4E-01	1.81	1.4E-04	1.21	6.7E-02	2.98	1.1E-04	1700071M16Rik	RIKEN cDNA 1700071M16 gene
ENSMUSG00000053070	2.04	3.5E-02	1.17	6.2E-01	2.12	2.8E-02	0.33	9.9E-01	2.47	4.9E-03	1.57	9.1E-01	2.78	4.7E-02	9230110C19Rik	RIKEN cDNA 9230110C19 gene
ENSMUSG00000032915	0.44	1.2E-02	0.33	4.3E-01	0.71	7.6E-03	-0.32	4.5E-01	0.68	4.9E-03	0.31	1.1E-01	0.72	3.4E-02	Adgre4	adhesion G protein-coupled receptor E4
ENSMUSG00000019806	0.40	8.7E-03	0.27	6.6E-02	0.71	6.8E-04	0.17	2.1E-01	0.30	4.3E-02	0.32	1.7E-01	0.71	9.4E-03	Aig1	androgen-induced 1
ENSMUSG00000035561	0.81	7.2E-03	0.23	4.0E-01	1.16	6.1E-03	0.25	5.1E-01	1.01	1.3E-02	0.46	8.0E-02	1.47	6.5E-06	Aldh1b1	aldehyde dehydrogenase 1 family, member B1
ENSMUSG00000032246	1.31	2.1E-02	-0.40	5.5E-01	1.44	5.5E-03	0.13	5.7E-01	1.63	1.8E-02	1.02	2.9E-01	3.45	5.1E-03	Calml4	calmodulin-like 4
ENSMUSG00000018927	0.63	3.2E-03	0.19	2.7E-01	0.96	3.2E-03	0.26	8.4E-02	1.53	4.5E-02	1.60	1.8E-01	2.53	1.2E-02	Ccl6	chemokine (C-C motif) ligand 6
ENSMUSG00000034641	0.53	2.5E-02	0.23	5.3E-01	0.80	8.1E-03	-0.06	1.0E00	0.97	6.1E-03	0.70	2.3E-01	1.31	2.0E-03	Cd300ld	CD300 molecule like family member d
ENSMUSG00000014542	0.40	8.4E-03	0.21	2.1E-01	0.62	3.1E-03	0.15	4.0E-01	0.71	6.5E-04	0.36	1.9E-01	0.75	2.7E-02	Clec4f	C-type lectin domain family 4, member f
ENSMUSG00000004098	-2.20	4.3E-11	0.44	1.3E-01	-1.14	2.8E-05	0.11	9.3E-01	-2.18	1.9E-12	0.20	6.4E-01	-1.39	9.0E-05	Col5a3	collagen, type V, alpha 3
ENSMUSG00000030560	0.55	2.4E-04	0.30	1.1E-01	1.00	8.2E-04	-0.17	7.4E-01	0.63	3.5E-04	0.37	1.0E-01	0.91	3.9E-03	Ctsc	cathepsin C
ENSMUSG00000035000	1.89	1.9E-10	-0.08	6.8E-01	2.00	6.1E-16	0.02	9.4E-01	2.04	2.4E-19	0.19	5.8E-01	2.00	1.9E-08	Dpp4	dipeptidylpeptidase 4
ENSMUSG00000029270	-0.34	5.0E-02	-0.45	5.8E-02	-0.61	2.5E-02	-0.42	9.1E-02	-0.52	2.8E-02	-0.39	8.9E-02	-0.71	1.9E-02	Fam69a	family with sequence similarity 69, member A
ENSMUSG00000006205	0.73	3.0E-03	0.57	1.3E-01	0.79	1.2E-02	0.87	1.0E-01	1.55	3.8E-02	1.45	5.9E-02	2.29	9.2E-05	Htra1	HtrA serine peptidase 1
ENSMUSG00000059108	1.86	7.1E-03	1.19	1.3E-01	2.25	8.7E-04	-0.10	9.8E-01	2.48	7.3E-04	1.59	9.8E-02	3.23	1.2E-05	Ifitm6	interferon induced transmembrane protein 6
ENSMUSG00000020427	0.52	1.1E-02	0.49	3.0E-01	0.82	4.2E-03	0.15	7.0E-01	0.75	1.5E-03	0.58	7.3E-02	1.07	8.4E-03	Igfbp3	insulin-like growth factor binding protein 3
ENSMUSG00000032193	1.73	2.7E-19	0.41	1.2E-01	1.80	9.5E-14	-0.19	3.7E-01	1.82	7.2E-20	0.28	1.7E-01	1.93	1.1E-09	Ldlr	low density lipoprotein receptor
ENSMUSG00000005447	0.71	3.6E-03	0.58	1.3E-01	0.61	4.9E-02	0.39	2.8E-01	0.87	9.4E-03	0.99	1.3E-01	1.24	4.2E-03	Pafah1b3	platelet-activating factor acetylhydrolase, isoform 1b, subunit 3
ENSMUSG00000033278	0.50	2.8E-02	0.33	4.5E-01	0.83	1.9E-02	0.02	9.4E-01	1.02	8.5E-03	0.36	6.5E-02	1.36	2.1E-04	Ptprm	protein tyrosine phosphatase, receptor type, M
ENSMUSG00000091780	-0.47	1.5E-02	0.04	4.4E-01	-0.54	1.6E-02	0.07	7.1E-01	-0.77	4.5E-04	-0.28	5.0E-01	-0.79	1.2E-02	Sco2	SCO2 cytochrome c oxidase assembly protein
ENSMUSG00000025743	0.35	4.8E-02	0.24	3.9E-01	0.69	2.9E-02	-0.10	5.9E-01	0.72	4.5E-04	0.27	1.2E-01	1.35	1.2E-04	Sdc3	syndecan 3
ENSMUSG00000026581	0.74	3.4E-02	0.38	5.4E-01	1.36	8.6E-03	-0.04	7.6E-01	2.30	2.3E-04	0.97	2.8E-01	2.82	5.9E-04	Sell	selectin, lymphocyte
ENSMUSG00000057193	0.52	4.2E-02	-0.05	6.5E-01	0.76	3.2E-02	-0.07	8.8E-01	0.66	1.5E-02	0.44	1.5E-01	1.24	5.0E-04	Slc44a2	solute carrier family 44, member 2
ENSMUSG00000019872	0.41	3.6E-02	0.37	1.0E-01	0.54	9.5E-03	0.19	3.1E-01	0.70	2.1E-03	0.71	1.2E-01	0.91	1.8E-02	Smpdl3a	sphingomyelin phosphodiesterase, acid-like 3A
ENSMUSG00000020077	0.40	8.6E-03	0.04	6.4E-01	0.65	3.1E-02	0.15	3.3E-01	0.62	1.5E-02	0.99	2.1E-01	1.33	9.7E-03	Srgn	serglycin
ENSMUSG00000022885	0.43	4.5E-02	0.20	5.5E-01	0.66	2.4E-02	-0.15	3.0E-01	0.51	3.1E-02	0.31	4.6E-01	0.88	1.7E-02	St6gal1	beta galactoside alpha 2,6 sialyltransferase 1
ENSMUSG00000038540	1.78	6.7E-03	-0.90	5.1E-01	2.69	1.5E-02	0.20	6.8E-01	2.46	2.7E-02	0.76	2.9E-01	3.65	6.0E-08	Tmc3	transmembrane channel-like gene family 3
ENSMUSG00000022615	-0.72	6.6E-05	-0.02	8.6E-01	-0.87	1.4E-04	-0.06	7.1E-01	-0.96	4.3E-06	-0.30	3.3E-01	-1.03	1.1E-03	Tymp	thymidine phosphorylase
ENSMUSG00000044206	0.59	2.7E-04	0.21	5.7E-01	0.94	1.1E-03	0.03	5.4E-01	0.83	1.6E-03	0.54	7.2E-02	1.16	3.1E-03	Vsig4	V-set and immunoglobulin domain containing 4
ENSMUSG00000073643	-1.03	1.7E-06	-0.26	2.2E-01	-1.11	9.5E-05	-0.13	7.0E-01	-1.70	3.0E-16	-0.03	8.1E-01	-1.76	1.7E-07	Wdfy1	WD repeat and FYVE domain containing 1

semisynthetic diet specific genes

	regular chow						semisynthetic diet									
	$Ldlr^{-/-}$		$Mc4r^{mut}$		$Mc4r^{mut}$; $Ldlr^{-/-}$		wt		$Ldlr^{-/-}$		$Mc4r^{mut}$		$Mc4r^{mut}$; $Ldlr^{-/-}$			
**Ensembl Gene ID**	**log2 fold change**	**p-value**	**log2 fold change**	**p-value**	**log2 fold change**	**p-value**	**log2 fold change**	**p-value**	**log2 fold change**	**p-value**	**log2 fold change**	**p-value**	**log2 fold change**	**p-value**	**name**	**description**
ENSMUSG00000027327	0.09	2.7E-01	0.22	2.4E-01	-0.03	6.9E-01	0.46	4.8E-02	0.51	9.9E-03	0.63	1.2E-02	0.52	4.2E-02	1700037H04Rik	RIKEN cDNA 1700037H04 gene
ENSMUSG00000031974	-0.23	1.4E-01	-0.20	1.5E-01	-0.46	8.3E-02	-0.49	1.0E-02	-0.46	3.0E-02	-0.79	8.6E-03	-0.94	5.3E-03	Abcb10	ATP-binding cassette, sub-family B (MDR/TAP), member 10
ENSMUSG00000034254	0.19	2.8E-01	0.41	8.6E-02	0.44	1.4E-01	0.56	4.1E-02	0.62	4.6E-02	0.68	1.0E-02	0.71	2.3E-02	Agpat1	1-acylglycerol-3-phosphate O-acyltransferase 1 (lysophosphatidic acid acyltransferase, alpha)
ENSMUSG00000022763	-0.19	1.7E-01	-0.02	6.9E-01	-0.32	5.1E-01	-0.74	9.6E-04	-1.36	2.3E-07	-1.41	1.4E-04	-1.38	2.5E-04	Aifm3	apoptosis-inducing factor, mitochondrion-associated 3
ENSMUSG00000030695	-0.06	9.0E-01	0.31	1.1E-01	0.20	3.5E-01	0.62	2.3E-03	0.43	8.8E-03	0.67	6.5E-03	0.76	5.4E-03	Aldoa	aldolase A, fructose-bisphosphate
ENSMUSG00000050947	0.13	7.9E-01	-0.28	2.5E-01	-0.22	3.8E-01	-0.50	2.0E-02	-0.74	4.5E-03	-0.74	1.7E-02	-1.16	4.4E-03	Amigo1	adhesion molecule with Ig like domain 1
ENSMUSG00000055116	-0.38	3.9E-01	-0.55	6.6E-02	-0.67	4.0E-01	-1.06	3.9E-02	-1.75	9.2E-06	-1.69	1.2E-05	-1.61	9.4E-04	Arntl	aryl hydrocarbon receptor nuclear translocator-like
ENSMUSG00000029752	-0.01	5.8E-01	-0.70	2.9E-01	-0.73	7.8E-01	-2.74	1.2E-11	-2.64	1.8E-11	-3.27	1.4E-13	-2.48	2.4E-07	Asns	asparagine synthetase
ENSMUSG00000017929	0.05	6.8E-01	0.17	2.4E-01	-0.07	7.2E-01	0.64	3.4E-02	0.69	1.8E-02	0.66	3.8E-03	0.79	7.2E-03	B4galt5	UDP-Gal:betaGlcNAc beta 1,4-galactosyltransferase, polypeptide 5
ENSMUSG00000039653	-0.07	6.2E-01	-0.22	1.9E-01	-0.38	1.1E-01	-0.53	1.0E-02	-0.51	1.2E-02	-0.74	3.2E-03	-0.90	2.2E-03	Baat	bile acid-Coenzyme A: amino acid N-acyltransferase
ENSMUSG00000061132	0.14	7.3E-01	0.52	3.7E-01	0.53	8.5E-02	1.57	1.6E-02	1.39	9.4E-03	1.33	4.4E-03	2.14	2.0E-09	Blnk	B cell linker
ENSMUSG00000027559	-0.29	8.8E-02	-0.19	2.4E-01	-0.23	2.8E-01	-0.90	7.4E-04	-0.86	1.3E-03	-1.46	2.7E-08	-1.22	4.9E-06	Car3	carbonic anhydrase 3
ENSMUSG00000036526	0.43	4.4E-01	0.81	1.4E-01	0.50	9.7E-02	1.30	8.1E-03	1.24	1.4E-02	1.58	3.4E-03	2.49	7.6E-07	Card11	caspase recruitment domain family, member 11
ENSMUSG00000068114	-3.47	5.6E-02	-3.71	6.1E-02	-3.37	5.3E-02	-3.74	3.6E-02	-3.97	4.0E-02	-4.16	3.8E-02	-4.20	4.9E-02	Ccdc134	coiled-coil domain containing 134
ENSMUSG00000070348	0.19	3.1E-01	0.34	4.2E-01	0.74	2.4E-01	1.88	1.8E-03	1.75	5.8E-04	2.37	5.4E-06	2.47	1.8E-12	Ccnd1	cyclin D1
ENSMUSG00000028459	0.01	5.7E-01	0.25	9.0E-01	0.31	3.5E-01	0.60	9.6E-03	0.54	3.5E-02	1.13	8.1E-04	1.33	2.7E-05	Cd72	CD72 antigen
ENSMUSG00000024610	0.21	9.3E-02	0.24	6.8E-02	0.73	2.3E-01	0.70	1.1E-02	0.76	1.2E-03	0.85	2.1E-03	1.98	9.5E-06	Cd74	CD74 antigen (invariant polypeptide of major histocompatibility complex, class II antigen-associated)
ENSMUSG00000046722	0.08	4.0E-01	0.41	8.2E-02	0.43	5.0E-02	0.47	1.9E-02	0.54	1.3E-02	0.84	3.6E-03	1.00	4.8E-04	Cdc42se1	CDC42 small effector 1
ENSMUSG00000040420	0.65	5.8E-01	0.78	3.4E-01	0.87	1.0E-01	1.29	9.4E-03	2.13	1.5E-03	1.85	2.8E-05	2.51	3.4E-06	Cdh18	cadherin 18
ENSMUSG00000015357	-0.22	2.1E-01	-0.23	3.6E-01	-0.38	1.6E-01	-0.75	1.9E-02	-1.12	4.9E-07	-1.08	9.5E-06	-1.18	6.4E-04	Clpx	caseinolytic mitochondrial matrix peptidase chaperone subunit
ENSMUSG00000024900	-0.03	7.8E-01	0.18	2.1E-01	0.31	3.6E-01	0.56	1.6E-02	0.75	2.2E-02	0.67	4.3E-03	0.85	7.7E-03	Cpt1a	carnitine palmitoyltransferase 1a, liver
ENSMUSG00000031825	0.58	1.2E-01	0.29	5.8E-01	1.17	5.8E-02	1.35	3.8E-02	1.48	3.7E-02	1.73	4.5E-02	2.26	1.4E-05	Crispld2	cysteine-rich secretory protein LCCL domain containing 2
ENSMUSG00000059326	0.31	1.7E-01	0.35	2.6E-01	0.72	1.8E-01	0.74	2.6E-02	0.70	9.9E-03	1.08	6.3E-04	1.81	1.3E-06	Csf2ra	colony stimulating factor 2 receptor, alpha, low-affinity (granulocyte-macrophage)
ENSMUSG00000031360	0.11	5.3E-01	0.17	7.2E-01	0.27	2.3E-01	0.43	1.7E-02	0.49	9.4E-03	0.96	2.2E-03	0.94	1.7E-03	Ctps2	cytidine 5'-triphosphate synthase 2
ENSMUSG00000034855	-0.47	9.9E-01	0.01	6.1E-01	0.93	1.3E-01	0.76	4.2E-02	0.46	3.6E-02	1.41	2.3E-02	1.23	4.4E-04	Cxcl10	chemokine (C-X-C motif) ligand 10
ENSMUSG00000003053	0.43	3.6E-01	-0.17	1.3E-01	0.08	7.0E-01	-0.64	9.0E-03	-0.52	3.8E-02	-1.32	1.1E-06	-1.48	1.5E-08	Cyp2c29	cytochrome P450, family 2, subfamily c, polypeptide 29
ENSMUSG00000052974	-0.01	7.5E-01	-0.33	1.3E-01	-0.49	1.6E-01	-0.63	2.5E-03	-0.68	4.1E-03	-0.74	1.4E-02	-0.82	6.6E-03	Cyp2f2	cytochrome P450, family 2, subfamily f, polypeptide 2
ENSMUSG00000056035	0.07	3.9E-01	-0.12	4.9E-01	-0.01	6.1E-01	-1.71	2.0E-05	-1.92	1.4E-05	-1.41	4.6E-05	-1.33	2.8E-04	Cyp3a11	cytochrome P450, family 3, subfamily a, polypeptide 11
ENSMUSG00000038656	-1.75	1.4E-01	-0.18	7.4E-01	-0.80	2.8E-01	-4.39	4.5E-03	-7.08	2.9E-04	-6.54	7.2E-04	-3.99	1.6E-02	Cyp3a16	cytochrome P450, family 3, subfamily a, polypeptide 16
ENSMUSG00000029630	0.04	4.4E-01	-0.08	3.6E-01	0.02	7.0E-01	-0.80	1.9E-06	-0.69	6.9E-05	-0.55	9.1E-03	-0.81	5.2E-04	Cyp3a25	cytochrome P450, family 3, subfamily a, polypeptide 25
ENSMUSG00000075552	-2.31	1.1E-01	-0.42	7.1E-01	-0.78	2.4E-01	-5.21	3.7E-03	-6.97	7.6E-04	-5.93	4.5E-03	-4.61	1.7E-02	Cyp3a41b	cytochrome P450, family 3, subfamily a, polypeptide 41B
ENSMUSG00000054417	-2.72	5.6E-02	-0.63	7.4E-01	-0.97	1.7E-01	-5.21	4.4E-03	-6.36	1.9E-03	-5.48	6.6E-03	-3.87	3.9E-02	Cyp3a44	cytochrome P450, family 3, subfamily a, polypeptide 44

ENSMUSG00000061292	-0.46	6.0E-02	-0.23	2.1E-01	0.00	2.3E-01	-1.74	5.4E-04	-1.44	1.9E-03	-0.96	5.9E-03	-0.94	4.7E-03	Cyp3a59	cytochrome P450, family 3, subfamily a, polypeptide 59
ENSMUSG00000001467	-0.13	4.6E-01	-0.21	8.5E-02	-0.46	5.1E-02	-1.40	3.2E-10	-0.86	3.1E-05	-1.08	2.5E-05	-0.72	2.6E-02	Cyp51	cytochrome P450, family 51
ENSMUSG00000059824	0.44	6.8E-01	0.43	3.4E-01	0.44	7.5E-01	2.37	4.3E-02	2.23	2.1E-04	2.39	3.0E-04	1.80	2.8E-02	Dbp	D site albumin promoter binding protein
ENSMUSG00000019891	0.07	7.5E-01	0.31	3.5E-01	0.69	5.3E-02	0.69	2.7E-02	0.89	2.1E-03	0.93	5.2E-03	0.97	1.5E-02	Dcbld1	discoidin, CUB and LCCL domain containing 1
ENSMUSG00000061322	0.92	3.5E-01	-2.13	5.9E-02	-0.26	4.9E-01	-2.24	2.4E-02	-1.89	2.8E-02	-2.41	4.0E-02	-3.43	9.4E-03	Dnaic1	dynein, axonemal, intermediate chain 1
ENSMUSG00000020057	0.67	1.5E-01	0.92	1.1E-01	1.30	7.9E-02	0.97	4.1E-02	1.27	5.5E-03	1.16	2.1E-02	2.06	3.5E-04	Dram1	DNA-damage regulated autophagy modulator 1
ENSMUSG00000019960	-0.03	7.7E-01	0.16	8.2E-01	0.07	8.6E-01	0.81	4.4E-03	0.51	3.2E-02	1.04	7.7E-03	0.73	2.3E-02	Dusp6	dual specificity phosphatase 6
ENSMUSG00000040659	0.23	1.3E-01	0.26	1.8E-01	0.19	4.5E-01	0.70	1.9E-03	0.51	9.5E-03	0.75	1.8E-02	0.56	4.4E-02	Efhd2	EF hand domain containing 2
ENSMUSG00000064254	-0.08	5.7E-01	0.09	8.6E-01	-0.32	3.2E-01	-0.52	1.1E-02	-0.78	1.3E-03	-0.56	2.4E-02	-0.88	7.7E-03	Ethe1	ethylmalonic encephalopathy 1
ENSMUSG00000028128	0.04	8.8E-01	0.26	2.6E-01	0.03	9.6E-01	0.96	9.3E-03	0.63	1.1E-02	1.06	8.2E-04	0.91	1.8E-02	F3	coagulation factor III
ENSMUSG00000024664	0.19	3.7E-01	0.08	9.6E-01	0.27	4.1E-01	0.62	3.1E-02	0.92	2.9E-04	1.38	9.4E-04	1.64	5.8E-06	Fads3	fatty acid desaturase 3
ENSMUSG00000072568	0.35	4.1E-01	0.50	2.2E-01	1.06	7.7E-02	0.97	4.5E-03	1.16	2.0E-03	1.68	3.7E-05	2.23	3.0E-07	Fam84b	family with sequence similarity 84, member B
ENSMUSG00000039899	0.23	1.6E-01	-0.15	9.4E-01	1.04	6.9E-02	0.68	1.6E-02	1.56	2.3E-05	0.80	4.9E-02	2.13	2.7E-04	Fgl2	fibrinogen-like protein 2
ENSMUSG00000040170	-0.32	2.7E-01	0.24	8.2E-01	0.58	4.6E-01	0.85	3.6E-02	1.30	1.1E-02	1.38	4.4E-03	2.09	2.0E-02	Fmo2	flavin containing monooxygenase 2
ENSMUSG00000033066	-0.15	2.3E-01	-0.27	9.7E-02	-0.44	1.5E-01	-0.78	2.3E-03	-1.10	3.3E-04	-0.77	5.4E-04	-0.89	1.6E-02	Gas7	growth arrest specific 7
ENSMUSG00000028270	0.53	8.0E-02	0.36	8.2E-02	1.29	2.4E-01	0.90	3.1E-03	1.01	3.8E-05	1.35	9.7E-04	1.32	1.3E-03	Gbp2	guanylate binding protein 2
ENSMUSG00000028268	0.23	6.6E-02	0.20	5.2E-01	1.00	1.3E-01	0.95	1.4E-04	1.06	9.9E-08	1.04	2.2E-04	1.16	1.8E-04	Gbp3	guanylate binding protein 3
ENSMUSG00000086136	-0.05	8.6E-01	-0.64	1.2E-01	-0.64	1.7E-01	-1.44	1.3E-04	-1.67	2.1E-06	-3.44	1.2E-14	-4.17	1.4E-14	Gm12718	predicted gene 12718
ENSMUSG00000083287	-0.17	6.0E-01	-0.22	1.6E-01	-0.52	6.3E-02	-1.68	2.3E-10	-0.97	4.7E-06	-1.46	4.6E-08	-0.83	5.5E-03	Gm13502	predicted gene 13502
ENSMUSG00000084780	-0.26	8.7E-01	-0.88	1.2E-01	-1.12	9.1E-02	-1.67	2.3E-03	-1.51	5.7E-03	-2.50	2.7E-05	-2.46	7.4E-03	Gm15350	predicted gene 15350
ENSMUSG00000086868	-0.01	7.1E-01	-0.40	6.1E-02	-0.55	1.5E-01	-0.68	1.3E-03	-0.67	5.7E-03	-0.82	1.2E-02	-0.80	8.9E-03	Gm15883	predicted gene 15883
ENSMUSG00000059571	-0.42	2.1E-01	-0.20	5.5E-01	-0.66	2.0E-01	-0.99	2.5E-02	-1.29	3.7E-03	-1.31	2.3E-03	-1.22	3.6E-02	Gm16218	predicted gene 16218
ENSMUSG00000091833	-0.54	3.5E-01	-0.62	3.1E-01	-0.86	2.0E-01	-1.25	3.8E-02	-1.24	2.1E-02	-1.50	7.3E-03	-1.68	2.5E-02	Gm17317	predicted gene, 17317
ENSMUSG00000085692	-0.22	4.3E-01	0.33	8.6E-01	0.06	6.1E-01	-0.73	3.2E-02	-0.73	3.7E-02	-0.89	1.0E-02	-1.05	4.0E-02	Gm2061	predicted gene 2061
ENSMUSG00000073421	0.22	8.2E-02	0.18	1.2E-01	0.62	3.7E-01	0.86	2.8E-03	0.67	8.9E-03	0.99	6.6E-05	2.02	5.2E-07	H2-Ab1	histocompatibility 2, class II antigen A, beta 1
ENSMUSG00000037649	0.28	7.2E-02	0.44	1.2E-01	0.83	1.1E-01	0.70	7.7E-03	1.03	2.2E-04	1.12	5.1E-04	2.01	2.0E-08	H2-DMa	histocompatibility 2, class II, locus DMa
ENSMUSG00000031770	-0.17	4.4E-01	-0.36	8.0E-02	-0.50	1.1E-01	-0.54	2.1E-02	-0.60	2.3E-02	-0.90	2.4E-03	-1.06	1.3E-03	Herpud1	homocysteine-inducible, endoplasmic reticulum stress-inducible, ubiquitin-like domain member 1
ENSMUSG00000032115	-0.13	8.7E-01	-0.62	6.0E-02	-0.95	8.4E-02	-1.24	4.3E-03	-1.11	1.4E-02	-1.60	2.9E-04	-1.10	1.4E-02	Hyou1	hypoxia up-regulated 1
ENSMUSG00000058258	-0.17	5.9E-01	-0.24	1.7E-01	-0.57	9.1E-02	-1.70	5.7E-10	-1.01	5.4E-06	-1.51	1.1E-08	-0.87	3.5E-03	Idi1	isopentenyl-diphosphate delta isomerase
ENSMUSG00000062488	0.33	1.2E-01	0.23	4.9E-01	0.83	8.1E-02	0.95	4.7E-03	1.21	6.5E-05	1.26	3.5E-03	1.18	3.3E-03	Ifit3b	interferon-induced protein with tetratricopeptide repeats 3B
ENSMUSG00000020009	0.09	2.5E-01	0.02	7.4E-01	0.34	6.6E-02	0.57	3.4E-02	0.63	5.9E-03	0.57	1.0E-02	1.04	1.0E-03	Ifngr1	interferon gamma receptor 1
ENSMUSG00000036256	0.11	2.5E-01	0.12	5.7E-01	0.31	3.1E-01	0.47	3.7E-02	0.46	4.0E-02	0.60	1.1E-02	0.82	1.3E-03	Igfbp7	insulin-like growth factor binding protein 7
ENSMUSG00000038034	0.29	4.5E-01	0.41	7.8E-02	0.57	5.0E-02	0.60	2.7E-02	0.47	4.8E-02	0.87	1.6E-02	1.00	4.0E-04	Igsf8	immunoglobulin superfamily, member 8
ENSMUSG00000073859	0.40	1.8E-01	0.29	3.3E-01	0.47	1.4E-01	0.67	3.3E-02	0.70	4.1E-02	1.04	7.5E-03	1.37	2.9E-03	Itpripl2	inositol 1,4,5-triphosphate receptor interacting protein-like 2

ENSMUSG00000033880	0.03	6.4E-01	0.41	7.7E-02	0.39	9.2E-02	0.53	2.0E-02	0.52	2.3E-03	0.84	7.1E-04	1.16	9.0E-05	Lgals3bp	lectin, galactoside-binding, soluble, 3 binding protein
ENSMUSG00000050199	-0.11	7.4E-01	-0.37	1.0E-01	-0.34	2.6E-01	-0.53	1.2E-02	-0.47	4.8E-02	-0.76	1.0E-02	-0.79	1.5E-02	Lgr4	leucine-rich repeat-containing G protein-coupled receptor 4
ENSMUSG00000038668	-0.10	7.8E-01	0.92	6.0E-02	0.44	6.5E-01	1.02	4.4E-02	1.30	4.8E-02	1.73	5.1E-03	1.77	7.7E-05	Lpar1	lysophosphatidic acid receptor 1
ENSMUSG00000069662	0.22	8.7E-02	0.32	1.6E-01	0.42	5.9E-02	0.44	3.6E-02	0.54	2.8E-02	0.81	3.6E-03	1.41	2.2E-03	Marcks	myristoylated alanine rich protein kinase C substrate
ENSMUSG00000030605	0.16	1.4E-01	0.37	1.2E-01	0.38	2.1E-01	0.59	2.7E-02	0.77	7.6E-03	0.95	2.4E-03	1.63	1.9E-07	Mfge8	milk fat globule-EGF factor 8 protein
ENSMUSG00000068566	0.07	5.0E-01	0.07	7.4E-01	0.15	7.7E-01	0.50	3.4E-02	0.49	4.5E-02	0.47	3.4E-02	0.71	1.4E-02	Myadm	myeloid-associated differentiation marker
ENSMUSG00000030004	0.17	8.7E-01	-0.29	2.9E-01	-0.29	4.8E-01	-1.03	8.9E-05	-1.01	2.5E-04	-1.44	1.3E-06	-1.51	9.0E-06	Nat8	N-acetyltransferase 8 (GCN5-related)
ENSMUSG00000041827	0.25	1.9E-01	0.51	1.9E-01	0.38	1.2E-01	0.69	1.0E-02	0.97	2.6E-02	0.82	6.2E-03	0.99	1.8E-03	Oasl1	2'-5' oligoadenylate synthetase-like 1
ENSMUSG00000029561	0.01	3.4E-01	0.29	3.6E-01	0.65	6.4E-02	0.95	1.8E-04	1.16	4.6E-05	1.40	1.2E-03	1.72	2.7E-06	Oasl2	2'-5' oligoadenylate synthetase-like 2
ENSMUSG00000031072	0.11	6.4E-01	-0.07	8.2E-01	0.31	2.8E-01	0.62	8.8E-03	0.47	3.7E-02	0.78	7.1E-03	0.74	2.4E-02	Oraov1	oral cancer overexpressed 1
ENSMUSG00000030774	0.30	2.7E-01	0.07	8.3E-01	0.74	8.9E-02	0.72	3.8E-02	0.77	3.1E-02	1.15	1.6E-02	2.23	1.2E-08	Pak1	p21 protein (Cdc42/Rac)-activated kinase 1
ENSMUSG00000022844	-0.02	4.2E-01	-0.54	6.3E-02	-0.48	2.7E-01	-0.65	2.2E-02	-0.68	8.0E-03	-1.15	7.7E-04	-1.14	4.7E-03	Pdia5	protein disulfide isomerase associated 5
ENSMUSG00000022090	0.07	9.3E-01	0.66	1.1E-01	0.84	9.4E-02	0.96	3.4E-02	1.35	6.1E-04	1.60	1.2E-03	2.30	1.5E-04	Pdlim2	PDZ and LIM domain 2
ENSMUSG00000056131	0.04	6.2E-01	0.38	1.1E-01	0.13	6.2E-01	0.77	3.4E-04	0.68	2.5E-03	0.73	5.5E-03	0.58	4.5E-02	Pgm3	phosphoglucomutase 3
ENSMUSG00000019461	-0.01	5.8E-01	0.40	7.9E-02	0.46	1.4E-01	0.58	2.5E-02	0.49	1.8E-02	0.83	2.0E-03	1.05	7.3E-04	Plscr3	phospholipid scramblase 3
ENSMUSG00000026748	0.55	6.5E-01	0.73	1.8E-01	1.35	8.2E-02	1.57	4.6E-02	2.39	8.2E-03	2.04	3.2E-03	3.17	5.5E-06	Plxdc2	plexin domain containing 2
ENSMUSG00000024640	-0.35	8.7E-01	0.05	8.6E-01	0.40	5.1E-01	0.93	2.2E-02	0.96	4.9E-02	1.75	4.5E-02	1.80	8.8E-04	Psat1	phosphoserine aminotransferase 1
ENSMUSG00000048271	-0.06	5.4E-01	-0.35	1.9E-01	-0.39	8.8E-02	-0.41	3.8E-02	-0.39	2.3E-02	-0.60	2.5E-02	-0.71	1.8E-02	Rbm33	RNA binding motif protein 33
ENSMUSG00000019539	0.46	1.2E-01	0.65	5.7E-02	0.69	2.8E-01	0.96	5.7E-03	1.13	3.6E-03	0.98	1.2E-03	1.18	2.8E-04	Rcn3	reticulocalbin 3, EF-hand calcium binding domain
ENSMUSG00000019189	0.00	8.5E-01	0.47	1.3E-01	0.39	9.4E-02	0.65	9.2E-03	0.87	2.6E-03	1.24	5.2E-05	1.08	1.1E-03	Rnf145	ring finger protein 145
ENSMUSG00000020641	0.20	1.3E-01	0.18	3.4E-01	0.53	9.6E-02	0.95	2.3E-02	0.88	1.5E-03	0.74	2.1E-03	0.90	5.5E-04	Rsad2	radical S-adenosyl methionine domain containing 2
ENSMUSG00000033355	0.11	4.5E-01	0.05	5.1E-01	0.34	3.0E-01	0.65	1.8E-03	0.79	2.9E-03	0.51	3.3E-02	0.78	1.9E-02	Rtp4	receptor transporter protein 4
ENSMUSG00000040808	0.61	1.6E-01	0.27	9.1E-01	0.86	1.3E-01	1.28	5.1E-03	1.47	3.9E-03	1.97	1.3E-02	1.88	7.5E-04	S100g	S100 calcium binding protein G
ENSMUSG00000030340	-0.33	1.9E-01	-0.47	6.9E-02	-0.49	6.0E-02	-0.46	1.1E-02	-0.74	3.1E-04	-1.53	1.6E-05	-1.73	3.6E-07	Scnn1a	sodium channel, nonvoltage-gated 1 alpha
ENSMUSG00000048163	0.24	2.1E-01	0.33	3.2E-01	0.79	1.3E-01	0.49	4.4E-02	0.84	1.4E-03	0.84	2.6E-03	1.69	1.3E-06	Selplg	selectin, platelet (p-selectin) ligand
ENSMUSG00000037411	-0.77	6.5E-01	0.00	5.3E-01	0.77	1.1E-01	1.54	4.1E-02	1.08	1.8E-03	2.58	2.7E-02	2.20	4.9E-07	Serpine1	serine (or cysteine) peptidase inhibitor, clade E, member 1
ENSMUSG00000001095	-0.13	8.3E-01	-0.82	4.2E-01	-1.12	3.7E-01	-2.92	5.4E-06	-2.78	5.3E-05	-4.87	7.1E-08	-3.25	3.8E-05	Slc13a2	solute carrier family 13 (sodium-dependent dicarboxylate transporter), member 2
ENSMUSG00000085747	-0.34	6.4E-01	-1.02	3.7E-01	-1.14	3.3E-01	-2.79	2.1E-03	-1.86	3.4E-02	-5.76	4.1E-05	-3.28	1.2E-02	Slc13a2os	solute carrier family 13 (sodium-dependent dicarboxylate transporter), member 2, opposite strand
ENSMUSG00000018566	0.73	2.3E-01	1.15	1.6E-01	1.14	6.0E-02	3.09	2.1E-02	3.62	3.3E-03	2.47	3.6E-05	2.40	1.7E-04	Slc2a4	solute carrier family 2 (facilitated glucose transporter), member 4
ENSMUSG00000026614	-0.03	6.5E-01	0.06	8.3E-01	0.00	6.1E-01	-0.64	2.0E-02	-0.69	3.9E-03	-0.99	4.3E-05	-1.08	3.2E-04	Slc30a10	solute carrier family 30, member 10
ENSMUSG00000030089	0.55	2.0E-01	0.58	5.4E-02	1.19	1.0E-01	1.35	2.5E-02	1.82	3.3E-02	1.40	1.0E-02	1.79	6.6E-05	Slc41a3	solute carrier family 41, member 3
ENSMUSG00000026435	-0.13	5.3E-01	-0.22	2.8E-01	-0.27	3.4E-01	-1.00	2.8E-04	-0.75	6.0E-03	-1.07	2.5E-03	-0.81	1.3E-02	Slc45a3	solute carrier family 45, member 3
ENSMUSG00000021007	0.56	5.2E-02	0.53	5.7E-02	0.63	1.2E-01	0.75	4.1E-02	0.53	4.3E-02	1.24	1.4E-03	0.78	4.7E-02	Spata7	spermatogenesis associated 7
ENSMUSG00000022364	0.17	9.8E-01	1.09	9.6E-02	1.50	9.2E-02	0.98	3.0E-03	1.90	1.7E-06	2.10	1.3E-06	2.41	1.7E-10	Tbc1d31	TBC1 domain family, member 31

ENSMUSG00000035493	0.07	4.4E-01	0.26	1.7E-01	0.28	2.8E-01	0.44	2.5E-02	0.59	1.2E-03	0.75	2.0E-03	1.03	2.2E-04	Tgfbi	transforming growth factor, beta induced
ENSMUSG00000024799	0.01	8.0E-01	0.09	1.0E00	-0.09	5.5E-01	-0.67	3.1E-04	-0.42	2.3E-02	-0.91	4.2E-04	-0.78	1.3E-02	Tm7sf2	transmembrane 7 superfamily member 2
ENSMUSG00000066258	0.04	8.7E-01	0.38	8.2E-02	0.18	8.8E-01	0.78	6.3E-04	0.71	3.9E-02	0.85	1.9E-03	0.68	3.2E-02	Trim12a	tripartite motif-containing 12A
ENSMUSG00000057596	-0.14	7.8E-01	0.30	2.6E-01	-0.01	5.7E-01	0.66	1.5E-04	0.70	9.2E-03	0.63	8.4E-03	0.49	3.8E-02	Trim30d	tripartite motif-containing 30D
ENSMUSG00000056144	-0.14	1.0E00	0.18	2.3E-01	0.17	6.2E-01	0.64	1.1E-04	0.64	5.3E-03	0.80	9.5E-04	0.71	3.5E-03	Trim34a	tripartite motif-containing 34A
ENSMUSG00000001763	-0.13	5.7E-01	-0.35	9.4E-02	-0.47	1.3E-01	-0.65	4.7E-03	-1.01	1.2E-05	-1.72	3.7E-08	-1.48	2.6E-06	Tspan33	tetraspanin 33
ENSMUSG00000054630	0.01	5.4E-01	-0.18	3.3E-01	-0.28	2.9E-01	-0.53	1.3E-02	-0.76	5.7E-05	-0.40	1.6E-02	-0.98	3.8E-04	Ugt2b5	UDP glucuronosyltransferase 2 family, polypeptide B5
ENSMUSG00000027962	0.36	8.6E-02	0.46	1.5E-01	1.22	1.2E-01	0.90	3.1E-03	0.96	3.8E-02	1.56	9.1E-05	2.19	2.0E-06	Vcam1	vascular cell adhesion molecule 1
ENSMUSG00000031016	0.62	5.9E-01	1.08	1.0E-01	1.29	5.6E-02	2.22	4.1E-02	2.53	4.9E-04	2.70	3.0E-05	2.46	1.0E-04	Wee1	WEE 1 homolog 1 (S. pombe)
ENSMUSG00000057842	-0.17	4.0E-01	-0.33	1.1E-01	-0.36	1.1E-01	-0.55	1.1E-02	-0.28	2.0E-02	-0.47	4.2E-02	-0.74	3.6E-02	Zfp595	zinc finger protein 595
