# Supplementary material for: Altered hepatic lipid metabolism in mice lacking both the melanocortin type 4 receptor and low density lipoprotein receptor
Source: PLoS One. 2017 Feb 16;12(2):e0172000. doi: 10.1371/journal.pone.0172000 (PMC5313158; doi:10.1371/journal.pone.0172000)
Supplement: S7 Table — 50 genes whose expression correlates with hepatic TAG levels in different diet-induced non-alcoholic fatty liver disease (NAFLD) mouse models [47] are compared with the results of the differential expression analysis in our mouse strains. + p value < 0.05, — p > 0.05. (PDF) [file pone.0172000.s010.pdf]

S7 Table.Genes expressed in liver known to correlate with hepatic triacylglycerol (TAG) levels.

	regular chow			semisynthetic diet					
**Ensembl Gene ID**	**Ldlr$^{-/-}$**	**Mc4r^{mut}**	**Mc4r^{mut}; Ldlr$^{-/-}$**	**wt**	**Ldlr$^{-/-}$**	**Mc4r^{mut}**	**Mc4r^{mut}; Ldlr$^{-/-}$**	**name**	**description**
ENSMUSG00000002944	+	+	+	+	+	+	+	Cd36	CD36 antigen
ENSMUSG00000002992	+	+	+	+	+	+	+	Apoc2	apolipoprotein C-II
ENSMUSG00000012187	-	+	+	+	+	+	+	Mogat1	monoacylglycerol O-acyltransferase 1
ENSMUSG00000017723	-	+	+	+	+	+	+	Wfdc2	WAP four-disulfide core domain 2
ENSMUSG00000020123	-	+	+	+	+	+	+	Avpr1a	arginine vasopressin receptor 1A
ENSMUSG00000030086	-	+	+	+	+	+	+	Chchd6	coiled-coil-helix-coiled-coil-helix domain containing 6
ENSMUSG00000031434	-	+	+	+	+	+	+	Morc4	microrchidia 4
ENSMUSG00000032231	-	+	+	+	+	+	+	Anxa2	annexin A2
ENSMUSG00000042041	+	+	+	+	+	+	+	2010003K1	RIKEN cDNA 2010003K11 gene
ENSMUSG00000051579	+	+	+	+	+	+	+	Tceal8	transcription elongation factor A (SII)-like 8
ENSMUSG00000054263	-	+	+	+	+	+	+	Lifr	leukemia inhibitory factor receptor
ENSMUSG00000020102	-	-	+	+	+	+	+	Slc16a7	solute carrier family 16 (monocarboxylic acid transporters), member 7
ENSMUSG00000020122	-	+	+	-	+	+	+	Egfr	epidermal growth factor receptor
ENSMUSG00000032802	-	+	+	-	+	+	+	Srxn1	sulfiredoxin 1 homolog (S. cerevisiae)
ENSMUSG00000040268	-	+	+	+	-	+	+	Plekha1	pleckstrin homology domain containing, family A (phosphoinositide binding specific) member 1
ENSMUSG00000041773	-	+	+	+	-	+	+	Enc1	ectodermal-neural cortex 1
ENSMUSG00000002831	-	+	+	-	-	+	+	Plin4	perilipin 4
ENSMUSG00000005054	-	-	+	+	+	-	+	Cstb	cystatin B
ENSMUSG00000023057	-	+	+	+	+	-	-	Fabp2	fatty acid binding protein 2, intestinal
ENSMUSG00000028672	-	+	+	+	+	-	-	Hmgcl	3-hydroxy-3-methylglutaryl-Coenzyme A lyase
ENSMUSG00000046324	-	+	+	-	-	+	+	Ermp1	endoplasmic reticulum metallopeptidase 1
ENSMUSG00000057103	-	+	-	-	+	+	+	Nat8f1	N-acetyltransferase 8 (GCN5-related) family member 1
ENSMUSG00000041959	+	+	+	+	-	-	-	S100a10	S100 calcium binding protein A10 (calpactin)
ENSMUSG00000052684	-	-	-	+	-	+	+	Jun	jun proto-oncogene
ENSMUSG00000055172	-	+	+	-	-	-	+	C1ra	complement component 1, r subcomponent A

	regular chow			semisynthetic diet					
**Ensembl Gene ID**	**Ldlr$^{-/-}$**	**Mc4r^{mut}**	**Mc4r^{mut}; Ldlr$^{-/-}$**	**wt**	**Ldlr$^{-/-}$**	**Mc4r^{mut}**	**Mc4r^{mut}; Ldlr$^{-/-}$**	**name**	**description**
ENSMUSG00000057236	-	-	+	-	-	+	+	Rbbp4	retinoblastoma binding protein 4
ENSMUSG00000072115	-	-	-	-	+	+	+	Ang	angiogenin, ribonuclease, RNase A family, 5
ENSMUSG00000021236	-	+	-	-	-	+	-	Entpd5	ectonucleoside triphosphate diphosphohydrolase 5
ENSMUSG00000027984	-	+	-	-	-	+	-	Hadh	hydroxyacyl-Coenzyme A dehydrogenase
ENSMUSG00000030555	-	-	+	-	+	-	-	Ttc23	tetratricopeptide repeat domain 23
ENSMUSG00000031443	-	-	-	-	-	+	+	F7	coagulation factor VII
ENSMUSG00000047797	-	-	-	-	-	+	+	Gjb1	gap junction protein, beta 1
ENSMUSG00000060002	-	+	+	-	-	-	-	Chpt1	choline phosphotransferase 1
ENSMUSG00000021134	-	-	-	-	-	+	-	Srsf5	serine/arginine-rich splicing factor 5
ENSMUSG00000027792	-	-	+	-	-	-	-	Bche	butyrylcholinesterase
ENSMUSG00000028557	-	-	+	-	-	-	-	Rnf11	ring finger protein 11
ENSMUSG00000038729	-	-	-	-	-	-	+	Akap2	A kinase (PRKA) anchor protein 2
ENSMUSG00000019873	-	-	-	-	-	-	-	Reep3	receptor accessory protein 3
ENSMUSG00000021996	-	-	-	-	-	-	-	Esd	esterase D/formylglutathione hydrolase
ENSMUSG00000024683	-	-	-	-	-	-	-	Mrpl16	mitochondrial ribosomal protein L16
ENSMUSG00000025159	-	-	-	-	-	-	-	Mms19	MMS19 (MET18 S. cerevisiae)
ENSMUSG00000027774	-	-	-	-	-	-	-	Gfm1	G elongation factor, mitochondrial 1
ENSMUSG00000028719	-	-	-	-	-	-	-	Cmpk1	cytidine monophosphate (UMP-CMP) kinase 1
ENSMUSG00000029310	-	-	-	-	-	-	-	Nudt9	nudix (nucleoside diphosphate linked moiety X)-type motif 9
ENSMUSG00000029462	-	-	-	-	-	-	-	Vps29	VPS29 retromer complex component
ENSMUSG00000030894	-	-	-	-	-	-	-	Tpp1	tripeptidyl peptidase I
ENSMUSG00000031357	-	-	-	-	-	-	-	Syap1	synapse associated protein 1
ENSMUSG00000034371	-	-	-	-	-	-	-	Tkfc	triokinase, FMN cyclase
ENSMUSG00000036309	-	-	-	-	-	-	-	Skp1a	S-phase kinase-associated protein 1A
ENSMUSG00000037236	-	-	-	-	-	-	-	Matr3	matrin 3

+ = p-value < 0.05 in differential expression analysis
- = p-value > 0.05 in differential expression analysis
