# Supplementary material for: Altered hepatic lipid metabolism in mice lacking both the melanocortin type 4 receptor and low density lipoprotein receptor
Source: PLoS One. 2017 Feb 16;12(2):e0172000. doi: 10.1371/journal.pone.0172000 (PMC5313158; doi:10.1371/journal.pone.0172000)
Supplement: S1 Methods — (PDF) [file pone.0172000.s011.pdf]

Supplementary Methods

Altered hepatic lipid metabolism in mice lacking both the melanocortin type 4 receptor and low density lipoprotein receptor

Vera Lede[1¶], Andrej Meusel[2¶], Antje Garten[3], Yulia Popkova[2], Melanie Penke[3], Christin Franke[4], Albert Ricken[5], Angela Schulz[1], Wieland Kiess[3], Daniel Huster[2], Torsten Schöneberg[1*], and Jürgen Schiller[2*]

[1]Molecular Biochemistry, Rudolf-Schönheimer-Institute of Biochemistry, University of Leipzig, Leipzig, Germany

[2]Institute of Medical Physics and Biophysics, University of Leipzig, Leipzig, Germany

[3]Hospital for Children & Adolescents, Department of Women and Child Health, Center for Pediatric Research Leipzig, University of Leipzig, Leipzig, Germany

[4]Heart and Vascular Center, Bad Bevensen, Germany

[5]Institute of Anatomy, Medical Faculty, University of Leipzig, Leipzig, Germany

*To whom correspondence should be addressed:

Email: juergen.schiller@medizin.uni-leipzig.de

Email: schoberg@medizin.uni-leipzig.de

[¶] Both authors contributed equally

Mice and knockout models

Using the chemical random mutagenesis technique with the germ line supermutagen N-ethyl-N-nitrosourea, a mouse model for mutant G protein-coupled receptors was generated by Ingenium Pharmaceuticals AG, Martinsried, Germany. Functional in vitro analysis of the mouse *Mc4r* containing the mutation Ile^{194}Phe revealed a partial loss of receptor function (~40-fold reduced agonist potency) (1). At the *in vivo* level, this mutant causes the full obese phenotype as observed in a mouse strain containing a *Mc4r* mutation (Tyr^{302}Cys) with a complete loss-of-function in in vitro assays. We used the Ile^{194}Phe mouse strain (for simplicity, we refer to the strain as $Mc4r^{mut}$) to closely mimic the receptor dysfunction most frequently found in humans (1). Mice were bred and maintained under specific-pathogen-free conditions at the centralized animal care facility, where lights were automatically controlled (12 h light/12 h dark). Mice were fed with a standard chow (composition given in S1 Table). All animal experiments were conducted in accordance with the European Directive 2010/63/EU on the protection of animals used for scientific purposes and were performed with permission of the Animal Care and Use Committee (ACUC #TVV 43/07) and the Government of the State of Saxony, Germany.

The initial Ile194Phe C3HeB/FeJ mouse strain was crossed into the C57/Bl6N (B6) background over more than 12 generations. Breeding was performed with heterozygous breeding pairs. Genotyping of the littermates was performed by PCR followed by BspHI (New England Biolabs, Frankfurt, Germany) restriction analysis. The following primers and PCR conditions were used: 5'-taccctgttaaacagtacggatac-3' (sense) and 5'-gaacatggaaatgaggcagatca-3' (antisense) creating a BspHI-site in $Mc4r^{+/+}$ sequence, conditions: 94 °C 3 min; 35 cycles of 94 °C 30 sec, 58 °C 30 sec and 72 °C 1 min. Products were digested with BspHI and fragments were separated in a 3 % agarose gel.

$Mc4r^{mut}$ mice were crossed onto a homozygous B6.$Ldlr^{-/-}$ background (The Jackson Laboratory, Bar Harbor, Maine, stock no. 002207) to generate double-deficient mice. These ($Mc4r^{mut}$;$Ldlr^{-/-}$) and respective controls ($Ldlr^{-/-}$) of both genders were weaned at 3 weeks of age and fed standard chow or semisynthetic diet until they were euthanized at 180 days of age. For euthanasia, mice were deeply narcotized by i.p. injection with a 100 µl mixture of 100 mg/kg body weight ketamine and 5 mg/kg body weight xylazine. Blood was collected post-euthanasia by heart puncture into syringes containing EDTA and the circulatory system was flushed with PBS (20 mL). Livers were also isolated from all animals

groups and frozen until further analysis could be performed. For morphological evaluation, livers were fixed in 4 % paraformaldehyde.

Morphological assessment

The right medial liver lobe was removed *in toto*, embedded in paraffin wax and sectioned 7 µm thick. Sections for routine histopathology were dewaxed, haematoxylin- and eosin (HE)-stained and covered with a glass coverslip using Histokitt (Carl Roth, Karlsruhe, Germany).

Sections for specific immunohistochemistry were selectively probed using anti-lipid droplet-associated perilipin 2 (Progen, Heidelberg, Germany) antibodies and an anti-leukocyte common differentiation antigen CD45 antibody (Clone 30-F11, BD Biosciences, Heidelberg, Germany). In brief, before staining antigenicity of the tissue was retrieved by heating the hydrated sections in sodium citrate buffer (pH 6, 5 min, 95 °C), and endogenous peroxidase activity was quenched by immersing the sections into a solution containing 3 % H_2O_2 and 10 % methanol for 30 min at room temperature. Incubations with the primary antibodies was performed at 4 °C overnight using the primary antibodies at a 1:50 (CD45 antibody) and a 1:500 (perilipin antibody) dilution, respectively. Binding of the primary antibodies was visualized with the avidin-biotin complex-technique using a species matched secondary antibody (VECTASTAIN Elite ABC Kit, Biozol, Eching, Germany). Immunohistochemical stained sections were counterstained with Mayer's haematoxylin before their embedding in Histokitt. HE and immunostained sections were evaluated under a microscope (Axioplan 2, Zeiss, Jena, Germany), photographed with a ProgRes C3 digital camera (Jenoptik, Jena, Germany) and documented with a digital recording system (ProgRes CapturePro 2.8.8, Jenoptik).

RNA sequencing of liver transcripts

Total RNA from liver was extracted by using the RNeasy Micro Kit™ (Qiagen, Hilden, Germany) as described in the manufacturer's instructions. The quantity of the RNA was measured using a spectrophotometer (NanoDrop 1000 (Thermo Scientific)) and RNA quality of all samples was examined on an Agilent 2100 bioanalyzer using the RNA 6000 Nano Chip (Agilent Technologies, Santa Clara, CA). We only included RNA samples with an RNA Integrity Number (RIN) value above 8.

Indexed cDNA libraries were generated using TruSeq RNA Sample Preparation Kits v2 (Illumina, San Diego, CA, USA) according to the manufacturer's protocol, constructing libraries with an average size

of 300 bp as evaluated on the Agilent 2100 bioanalyzer with DNA 1000 Chips. Libraries were sequenced on Illumina HiScanSQ Sequencing System, performing ten biological replicates for each genotype. 101-bp raw paired-end reads were generated on eight flow cell lanes (Core Unit “DNA Technologies”, University of Leipzig). Briefly, after quantification of the libraries using the Library Quantification Kit - Illumina/Universal (KAPABiosystems) according to the instructions of the manufacturer, products were used for cluster generation. Library DNA at a concentration of 10 pM was clustered using an Illumina cBot according to the PE_Amp_Lin_Block_Hybv8.0 protocol of the manufacturer. Sequencing was performed using version 3 chemistry and the version 3 flow cell according to the manufacturer’s instructions. Median cluster density was usually about 600,000 clusters per mm^2 or 80-100 million raw clusters per lane.

After intensities call, raw reads were separated according to library indexes allowing up to one mismatch in the index sequence, but requiring that all bases have a quality score above 15 (PHRED-scale). After assigning reads to samples we used an in-house-sequencing pipeline for adapter trimming and quality control. Reads were mapped to the reference mouse genome (July 2007 NCBI37/mm9) with Ensembl v66 annotations using Tophat 2.0.6. (2), which aligns reads using Bowtie2 (version 2.1.0). Reads which did not map uniquely to a genome position were excluded. The transcription level for each gene was obtained by intersecting mapping results with gene annotations using BEDTools IntersectBed (3). Using DESeq software package (4), differential expression of wt and knockout genes was examined. Only genes that were expressed at least in 10 animals were included for analyses. If not otherwise stated, differentially expressed genes with a p-value < 0.05 were considered as statistically significant.

Protein extraction and Western blot analyses

Approximately 10 mg liver tissue was lysed in modified RIPA buffer containing 50 mM Tris/HCl, pH 7.4; 1 % NP-40; 0.25 % sodium deoxycholate; Roche complete protease inhibitor cocktail; 1 mM EDTA; 1 mM sodium orthovanadate, 1 mM sodium fluoride, 5 mM nicotinamide, 5 µM Trichostatin A. Protein amount was determined using BCA protein assay (Pierce, Thermo Scientific). Proteins were separated by SDS-PAGE and semi-dry transferred to nitrocellulose membranes. Next, membranes were blocked in 5 % non-fat dry milk in TBS buffer containing 0.1 % Tween 20 (TBS-T). NAMPT monoclonal antibody (Clone OMNI 379, Cayman Chemical, dilution 1:1000) and NADK antibody (H-300, Santa Cruz, dilution 1:1000) were used in 5 % non-fat dry milk in T-TBS and the membrane was incubated overnight at

4°C. After washing with TBS-T, the membrane was incubated with goat anti rabbit horse radish peroxidase-conjugated antibody (DAKO) at room temperature for 1 h. Afterwards detection of proteins was carried out using Luminata Classico Western HRP Substrate (Merck Millipore). GAPDH immunoblotting was performed for normalization.

NAMPT ^{enzyme} assay

For determination of NAMPT activity 10 mg of liver tissue was lysed in 100 µl NAMPT enzyme assay buffer (0.1 M sodium phosphate, pH 7.4) and protein amount was determined using BCA protein assay. 30 µg of protein was added to the reaction buffer and incubated at 37 °C for 1 h. Afterwards the assay using radiolabeled ^{14}C-nicotinamide was performed as described before (5).

^{1}H HR MAS NMR Spectroscopy

For liver fat quantification, untreated liver tissue was transferred into a 4 mm zirconia magic angle spinning (MAS) rotor (Bruker, Rheinstetten, Germany) containing a spherical volume of 15 µl. NMR experiments were carried out on a Bruker Avance III 600 MHz NMR spectrometer, operating at a resonance frequency of 600.13 MHz using a 4-mm double resonance probe. ^{1}H NMR spectra were recorded under MAS conditions at a rotational frequency of 9 kHz and a temperature of 30 °C. To record NMR spectra for quantitative analysis, simple single $\pi/2$ pulse experiments were performed using a pulse length of 4 µs and a repetition time of 15 s to allow for complete relaxation. In addition, ^{1}H NMR spectra were recorded using water presaturation applying a low power pulse of 0.1 mW on the water resonance. A typical ^{1}H HR MAS NMR spectrum of a liver sample and the assignments of most prominent signals is shown as supplementary material (S1 Figure). Although the investigation of intact tissues by ^{1}H HR MAS NMR is accompanied by reduced resolution in comparison to solution state NMR, this approach has the significant advantage that all extraction losses are avoided. Acquired free induction decays were Fourier-transformed and phase corrected using TOPSPIN software. NMR signals were analyzed applying a mixed Lorentzian-Gaussian lineshape deconvolution for each resolved peak. As parameter for hepatic fat content (HFC), the sum of the peak integrals from 0.88 ppm to 2.8 ppm of the fatty acyl chains were calculated relative to the integral of the water signal (4.7 ppm). Signals between 0.88 ppm and 2.8 ppm are well characterized and correspond almost exclusively to lipids (6). Ratios calculated by this procedure were defined as HFC. Though not

representing the actual tissue fat content, this value serves as an approximation for the lipid accumulation in the liver as shown before (7, 8). The ratio of triacylglycerols (TAGs) as main storage lipids and the phospholipids (PL) as membrane lipids was determined by comparison of the peak integrals of the chain methyl- and choline (PC) signals. The choline group contains 9 protons and gives rise to a prominent signal at 3.2 ppm. The dominant choline source in cells is phosphatidylcholine (PC) bearing 6 fatty acyl chain methyl protons. TAGs have 9 methyl protons but no choline head group, while PLs other than PC contain 6 chain methyl protons. Thus, on the basis of these peak integrals, the contribution of PC relative to the total methyl signal and the approximate proportion of TAG and other PL in the sample can be calculated. The degree of acyl chain saturation of liver lipids was estimated by calculating the ratio of the methyl peak integral at 0.88 ppm and the integral of the signal at 2.8 ppm representing allylic protons occurring in polyunsaturated fatty acids (PUFAs) (9). The amount of monounsaturated fatty acids (MUFAs) was determined on the basis of the signal at 2.0 ppm representing protons contiguous to olefinic double bonds (10).

Lipid extraction and MALDI-TOF mass spectrometry

Frozen liver tissue was transferred into chloroform/methanol (2:1, v/v) and vortexed for at least 1 minute. Afterwards, the tissue extract was sonicated on ice for 1 min and shaken for 2 h at room temperature and 90 rpm. After shaking, 600 µl 0.9 % NaCl solution was added and samples were additionally vortexed for 1 min. Phase separation was achieved by 10 minutes of centrifugation at 2,500 rpm. The organic phase was transferred into a new sample tube and evaporated by vacuum centrifugation. The pellet was solubilized in 50 µl $CHCl_3$ and the solution was immediately used for mass spectrometric analysis or stored at -20 °C until further analysis.

Aliquots of 10 µl lipid extract were mixed with 10 µl 2,5-dihydroxybenzoic acid matrix (11) dissolved in isopropanol/acetonitrile (60:40 v/v containing 15 mM sodium acetate). A volume of 1 µl of this solution was transferred onto a MTP 384 aluminum coated target plate. After crystallization, MALDI-TOF mass spectra were recorded by using an Autoflex I mass spectrometer (Bruker Daltonics, Bremen, Germany). Ions were generated by averaging 100 shots from a N2 laser emitting at 337 nm at a frequency of 5 Hz. Spectra were recorded in the reflector mode using delayed ion extraction and gated matrix suppression. Mass spectra were recorded exclusively in the positive ion modus because the PC an TAG contents were of particular interest. Mass spectra were analyzed using FlexAnalysis

software version 2.2 (Bruker Daltonics). After peak assignment, PC and TAG fatty acyl compositions were determined by calculating the ratio of the intensity of a dedicated lipid species and the total intensities of all signals detected.

Supplementary References

1. Grosse J, Tarnow P, Römpler H, Schneider B, Sedlmeier R, Huffstadt U, Korthaus D, et al. N-ethyl-N-nitrosourea-based generation of mouse models for mutant G protein-coupled receptors. Physiol Genomics 2006;26:209-217.
2. Langmead B, Trapnell C, Pop M, Salzberg SL. Ultrafast and memory-efficient alignment of short DNA sequences to the human genome. Genome Biol 2009;10:R25.
3. Quinlan AR, Hall IM. BEDTools: a flexible suite of utilities for comparing genomic features. Bioinformatics 2010;26:841-842.
4. Anders S, Huber W. Differential expression analysis for sequence count data. Genome Biol 2010;11:R106.
5. Garten A, Petzold S, Barnikol-Oettler A, Korner A, Thasler WE, Kratzsch J, Kiess W, et al. Nicotinamide phosphoribosyltransferase (NAMPT/PBEF/visfatin) is constitutively released from human hepatocytes. Biochem Biophys Res Commun 2010;391:376-381.
6. Engel KM, Schrock K, Teupser D, Holdt LM, Tonjes A, Kern M, Dietrich K, et al. Reduced food intake and body weight in mice deficient for the G protein-coupled receptor GPR82. PLoS One 2011;6:e29400.
7. Martinez-Granados B, Monleon D, Martinez-Bisbal MC, Rodrigo JM, del Olmo J, Lluch P, Ferrandez A, et al. Metabolite identification in human liver needle biopsies by high-resolution magic angle spinning ^{1}H NMR spectroscopy. NMR Biomed 2006;19:90-100.
8. Springer F, Machann J, Claussen CD, Schick F, Schwenzer NF. Liver fat content determined by magnetic resonance imaging and spectroscopy. World J Gastroenterol 2010;16:1560-1566.
9. Huster D, Arnold K, Gawrisch K. Influence of docosahexaenoic acid and cholesterol on lateral lipid organization in phospholipid mixtures. Biochemistry 1998;37:17299-17308.
10. Popkova Y, Meusel A, Breitfeld J, Schleinitz D, Hirrlinger J, Dannenberger D, Kovacs P, et al. Nutrition-dependent changes of mouse adipose tissue compositions monitored by NMR, MS, and chromatographic methods. Anal Bioanal Chem 2015.
11. Schiller J, Arnhold J, Benard S, Muller M, Reichl S, Arnold K. Lipid analysis by matrix-assisted laser desorption and ionization mass spectrometry: A methodological approach. Anal Biochem 1999;267:46-56.
12. Hui ST, Parks BW, Org E, Norheim F, Che N, Pan C, Castellani LW, et al. The genetic architecture of NAFLD among inbred strains of mice. Elife 2015;4:e05607.
